# Enhancing Adhesion Properties of Commodity Polymers through Thiol-Catechol Connectivities: A Case Study on Polymerizing Polystyrene-Telechelics via Thiol-Quinone Michael-Polyaddition

**DOI:** 10.1021/acsmacrolett.4c00069

**Published:** 2024-03-28

**Authors:** Carolin M. Schröter, Lukas D. Bangert, Hans G. Börner

**Affiliations:** Department of Chemistry, Laboratory for Organic Synthesis of Functional Systems, Humboldt-Universität zu Berlin, Unter den Linden 6, 10099 Berlin, Germany

## Abstract

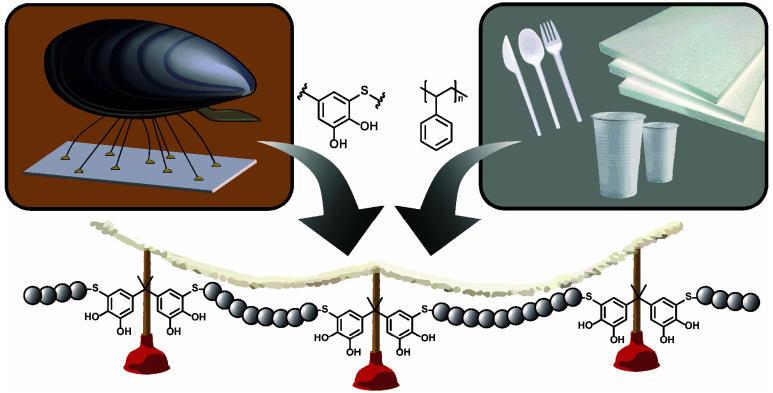

Segmented block copolymers with adhesive functionality bridges in between are synthesized through the combination of controlled radical polymerization (CRP) and thiol-quinone Michael-polyaddition. CRP provides a set of α,ω-dithiol polystyrenes (PS), which react as telechelics with a low molecular weight bisquinone, resulting in thiol-catechol connectivities (TCCs). By introducing as little as 3 mol % of TCC functionalities, the bonding of the polymer on dry and wet aluminum surfaces is significantly improved while keeping the integrity of the PS segments undisturbed to constitute favorable bulk properties. This improvement is evidenced by reaching up to 3.8 MPa adhesive strength, representing a 600% increase compared to nonfunctional PS.

Glues based on commodity monomers that can be cost-effectively synthesized via free-radical polymerization, e.g., from acrylates, acrylamides, or styrenes, offer tunability through copolymerization and give rise to successful adhesives based on styrene-acrylate- (SA), acrylonitrile-styrene-acrylate- (ASA), or styrene-butadiene- (SBS) copolymers.^1^ However, improving their bonding properties still holds promise for increased performance, therefore, broadening the applicability. Inspiration from biomaterials,^2^ such as the adhesive system of marine mussels,^3^ led to the utilization of 3,4-dihydroxyphenylalanine (DOPA)^4^ residues in adhesives. Catechol moieties have been shown to enhance both surface adhesion and material bulk cohesion in various synthetic polymers, leading to the development of a rich and diverse family of adhesives.^5,6^

The pioneering work of Wilker et al. enhanced adhesion of polystyrene (PS) on aluminum from 0.6 to ∼3 MPa by incorporating 3,4-dihydroxy styrene with an optimum of 33 mol %.^6^ However, the copolymer structure disturbs the PS chain–chain interactions, which could considerably enforce the bulk cohesion. It was found that 33 mol % functional monomers decreased the glass transition temperature (*T*_g_) from 106 to 62 °C.^6^

Macromolecular engineering^7^ suggests reducing this effect by utilizing a different chain architecture. Segmented copolymers^8^ with well-defined PS blocks connected by adhesive functionalities ideally maintain PS interactions, while improving adhesion.^9^

Recently, the thiol-quinone Michael-polyaddition route^10^ has been introduced as a clean variant of the thiol-Michael “Click” chemistry,^11,12^ yielding thiol-catechol connectivities (TCCs)^10^ as potent adhesive functionalities. This strategy proved to be versatile, leading to artificial mussel glue proteins or fully synthetic adhesive TCC-polymers.^10,11,13^ Peptide-based building blocks such as minimal tripeptides,^14^ peptides encoding for pH-induced β-sheet formation to activate cohesion,^15^ or ion-stimulated self-assembly for reversible responsiveness have been reported.^16^ The required quinones could be generated enzymatically or chemically with sodium periodate to oxidize the DOPA residues in situ. The latter was expanded to softwood lignin as multiphenol,^17^ where demethylation and oxidation could generate a quinone-rich lignin that reacts with multithiols and advent a new class of green TCC adhesives.

Moreover, fully synthetic analogues derived from commodity monomers such as bisquinones (AA) were used. The oxidation of bisphenol A (BPA) to bisquinone A (BQA) through a scalable reaction using 2-iodoxybenzoic acid (IBX) results in a high yield.^13,18^ BQA cleanly reacts with various small molecule dithiols (BB) in solution, generating a library of 40 different adhesive TCC-polymers.^19^ As the AA/BB quinone-thiol Michael-polyaddition proceeds under various conditions, the expansion to macromolecular dithiols gives access to segmented functional polymers.

Here, we expand the approach to TCC-polymer synthesis by utilizing a set of telechelic dithiol polymers. These were obtained by controlled radical polymerization (CRP) to react as α,ω-functional macromonomers with low molecular weight BQA, giving segmented TCC-polymers. PS was chosen as a cost-effective commodity polymer with suitable mechanical strength and stability under wet conditions.^20^ The resulting TCC-PS adhesives were analyzed for their performance at gluing various substrates.

The telechelic PS polymers were synthesized by reversible addition–fragmentation chain-transfer (RAFT) polymerization.^21^ A bifunctional chain transfer agent (CTA) was employed based on S-1-dodecyl-S′-(α,α′-dimethyl-α′′-acetic acid) trithiocarbonate^22,23^ to modulate the symmetric, bidirectional growth of styrene and yield three well-defined precursors, DiCTA-PS_1.7k_, DiCTA-PS_3.6k_, and DiCTA-PS_6.3k_. Size exclusion chromatography (SEC) confirmed low dispersities of *Đ* < 1.1. Average molecular weights of *M*_n_ = 1700, 3600, and 6300 g/mol correspond well with ^1^H NMR spectroscopy (Table S1), suggesting high end-group fidelity. Subsequently, aminolysis of DiCTA-PS_*x*_ transformed the trithiocarbonate groups into thiols, giving the desired telechelic Dithiol-PS_*x*_ (cf. SI).^21,24,25^ Quantitative cleavage for the entire set was confirmed by ^1^H NMR spectroscopy with the complete disappearance of CTA H_3_C-CH_2_– resonances and by a slight shift of the elution trace toward lower molecular weights in SEC. Consistent with the literature, a minor shoulder occurred at twice the peak molecular weight^26^ that likely results from thiol coupling. Nevertheless, the dispersities of all products remained low at *Đ* < 1.2 and dimerized chains can still be considered telechelic having two terminal thiol end groups. MALDI-ToF-MS analysis confirmed the structure of Dithiol-PS_1.7k_ and Dithiol-PS_3.6k_ by showing one dominating homologues row assigned with <1 Da accuracy to the expected structure and no DiCTA-PS_*x*_ signals.

To demonstrate the addition reaction also taking place with higher molecular weight dithiols and to facilitate analysis, a model reaction was performed (cf. SI). Dithiol-PS_1.7k_ was capped with 35 equiv of BQA, the remaining quinones at the BQA-capped end groups were subsequently quenched with 4-*tert*-butylbenzyl mercaptan as a ^1^H NMR probe, and the product was precipitated in methanol. MALDI-ToF-MS confirmed the introduction of two bisTCC structures (Figure S26) and ^1^H NMR analysis suggested a high end-group fidelity, revealing a stochiometric ratio of 2.1 *tert*-butyl end-caps to 4.0 solitary methyl groups of the central CTA group per 14 aromatic styrene segments (Figure S24). These calculations agreed well with SEC measurements noticing a shift of the *M*_p,app_ according to the increased mass while retaining a dispersity of *Đ* < 1.2. Similar results were found for Dithiol-PS_3.6k_ in the model reaction (cf. SI).

After conducting the promising model study, a polyaddition reaction was performed using Dithiol-PS_*x*_ and BQA in DMF, with conditions optimized to the highest molecular weights (Figure 1). The optimal ratio of functionalities was screened by changing the proportion of Dithiol-PS_*x*_ to BQA from 1.0:1.0 to 1.0:0.6 (molar T:Q ratio, thiol:quinone; Figure S41). The TCC-polymers achieved the highest molecular weight with 0.7–0.8 equiv of BQA, regardless of the Dithiol-PS_*x*_ (Table S4). A slight excess of the thiol component seems to be favorable, perhaps due to a combination of factors including minor disulfide formation, shielded end groups of the macromonomers, or TCC moieties that undergo a reoxidation to quinones and then form a second thiol connection. Due to the rapid Michael-type reaction, the presence of cyclic species could be envisioned^27^ and may also contribute to the thiol excess (Figure S34). MALDI-ToF-MS strongly suggests cyclic species that can be clearly differentiated from linear chains by missing end groups (Figure S34). Such ring formation has been observed with small monomers in earlier studies.^13^

**Figure 1:**
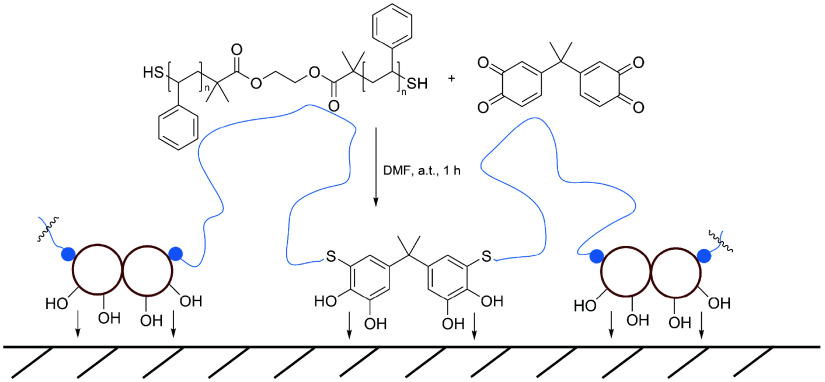
Synthesis of a segmented copolymer by AA+BB thiol-quinone polyaddition of telechelic Dithiol-PS with bisquinone A, reacting to thiol-catechol connectivities (TCCs) that might enhance adhesion at different substrate interfaces, while the PS segments contribute to cohesion.

The solution polymerization of Dithiol-PS_1.7k_ with BQA proved to be robust and could be performed at temperatures between 20 and 80 °C, without dramatic effects on the product molecular weight (Figure S42). Interestingly, despite higher molecular weight of the telechelic, the Ruggli–Ziegler principle was evident.^13,28^ Typical for polyaddition reactions, an increased monomer concentration resulted in higher molecular weight products, forming the largest TCC-polymers at *c*(BQA) = 5.00 g/L (Figure S43).

Under optimized conditions with a T/Q feed ratio of 1.0:0.7, *c*(BQA) = 5.00 g/L and ambient temperature in DMF, kinetic studies of the three Dithiol-PS_*x*_ telechelics with BQA were conducted. As anticipated, the reactions proceeded at a slower rate compared to smaller dithiols.^13^ UV–vis spectroscopy confirmed the disappearance of the BQA-characteristic absorption band at 380 nm within up to 24 h (Figure S40). As unreacted species mainly dominate the *M*_n_ of a polyaddition product at low conversion and for glues, cohesion and performance is strongly affected by higher molecular weight fractions, the maximum detectable molecular weight (*M*_max_) was used to follow the polyaddition over time.

Interestingly, SEC analysis revealed that TCC-polymers form rapidly with a molecular weight buildup leveling off within 30–60 min and only a slight increase up to 24 h (Figure 2a). The final *M*_max_ after 24 h allowed for the calculation of the apparent degree of polymerization (DP_max_) based on the average molecular weight of BQA and Dithiol-PS_*x*_ to reach ∼40, ∼70, and ∼100 for TCC-PS_1.7k_, TCC-PS_3.6k_, and TCC-PS_6.3k_, respectively (Figure 2b). As anticipated, larger PS telechelic polymerize to overall higher masses, but interestingly, also reach higher DP_max_ values.

**Figure 2:**
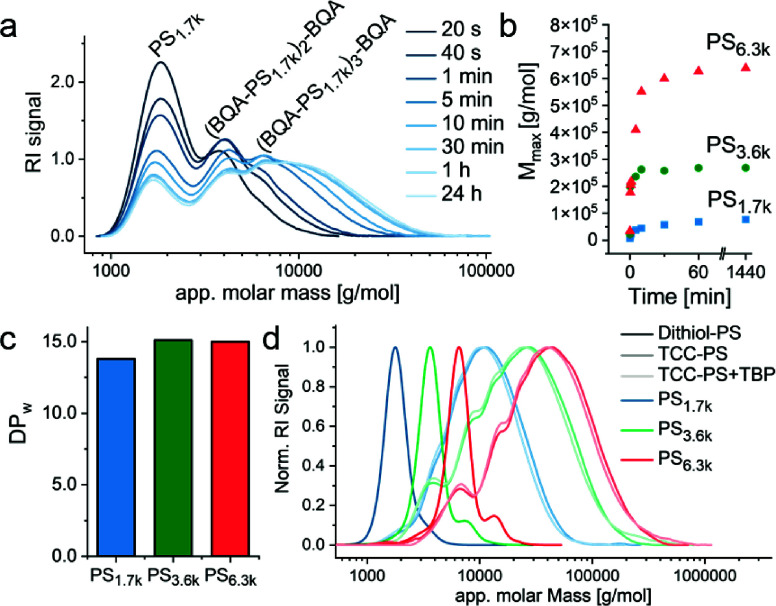
SEC measurements of the polymerization of Dithiol-PS telechelics with BQA show TCC-PS with a broad dispersity (a) and species in the high molecular weight range within 30–60 min (b). The precipitated polymers have a DP_w_ of 14–15 (c), and disulfide effects can be neglected (d).

For further analysis, the reactions were quenched with ethanethiol to react residual quinones to TCC functionalities before precipitation of the TCC-polymers in methanol. TCC-PS_1.7k_, TCC-PS_3.6k_, and TCC-PS_6.3k_ were isolated with yields of 65%, 88%, and 96%, respectively, for which SEC analysis gives *M*_w,app_ values of 14700 g/mol (*Đ* = 1.61), 33700 g/mol (*Đ* = 2.42), and 55800 g/mol (*Đ* = 2.43). TCC-PS_1.7k_ underwent considerable fractionation during purification, confirmed by a decrease in the dispersity and reduced yields compared to the other TCC-polymers. Taking the SEC data into account, a DP_w,app_ of around 14 was calculated for TCC-PS_1.7k_ and 15 for both TCC-PS_3.6k_ and TCC-PS_6.3k_ (Figure 2c). Comparing these results to literature for macromonomeric step-growth polymerization, values achieved are within the comparable range for DP_*n*_ of 8–10.^24,29^ To exclude contributions of disulfide formation for polymer growth as a potential alternative reaction, TCC-polymer solutions were incubated with tributyl phosphine (TBP) as reducing agent. Only marginal decreases in molecular weight were evident in SEC, excluding disulfide formation and indirectly supporting the growth via TCC formation (Figure 2d).

The presence of the important catechol groups in the TCC-PS_1.7k_ product was confirmed qualitatively through a colorimetric FeCl_3_ test, which showed the typical greenish color of the Fe^3+^ complex (Figure S38).^30^ This was consistent with IR spectroscopy evidencing an absorption band at ν = 1366 cm^–1^ characteristic to phenolic OH. For ^1^H NMR detection of the catechols, a chemical transformation with methyl iodide resulted in methoxy derivatives with proton resonances occurring at 3.69–3.99 ppm (Figure S37). ^31^P NMR measurements enabled quantification of phenolic hydroxyl groups by comparing the resonance intensity of phosphitylated catechols against that of an internal standard (Figure S31). 14, 7, and 4 wt % inbuild BQAs were found for TCC-PS_1.7k_, TCC-PS_3.6k_, and TCC-PS_6.3k_, respectively (corresponding to 13, 6, and 3 mol % TCCs per total aromatic units). This corresponds well with theoretical 13, 7, and 4 wt % BQA that could be expected to be incorporated, based on the *M*_n_ of the telechelic PS macromonomers. The amount of catechol derivatives is also well within the range of ∼10 mol % reported as suitable to bond aluminum.^31^ MALDI-ToF-MS analysis confirmed the chemical structure of TCC-PS_3.6k_ by showing one dominating homologue row that is well assignable (Figure S34).

The segmented PS polymers with adhesive TCC functionalities were thus successfully synthesized. While the molecular weight distributions exhibit appropriately high dispersities and, most importantly, sufficient fractions of the high molecular weight regime at 10^5^–10^6^ g/mol, adhesive tests had to elucidate applicability and adhesive performance. As anticipated, the TCC-PS_*x*_ adhesives show *T*_g_s that gradually approach 106 °C for pure PS as *T*_g_ increases as the TCC content decreases, from TCC-PS_1.7k_ with *T*_g_ = 86 °C, to TCC-PS_3.6k_ with *T*_g_ = 89 °C to TCC-PS_6.3k_ reaching *T*_g_ = 92 °C.

Lap shear tests were conducted on aluminum specimens, where the application of the TCC-PS_*x*_ polymers followed hotmelt-like protocols.^19^ The polymers were dissolved in acetone and 25 μL of a 2.5% solution were applied to one specimen. The solvent was allowed to evaporate before the second specimen was placed on top with a 5 × 20 mm overlap. After fixation with foldback clips, curing required 130 °C for 24 h to optimize the bonding interfaces. The shear strength did not increase with longer curing times, and sharp fracture patterns suggested the absence of softening solvents (Figure S48).

At first glance, it may seem counterintuitive that the adhesive forces were not increasing with TCC concentration but rather corresponded to the length of the undisturbed PS segments. Thus, the bonding improved consistently from TCC-PS_1.7k_ over TCC-PS_3.6k_ to TCC-PS_6.3k_, giving shear strengths of 1.47 ± 0.19 MPa, 2.07 ± 0.12 MPa and ultimately 3.77 ± 0.56 MPa. However, the importance of TCC-functionalities for the bond performance of PS was evident in a control experiment. Using pure PS that had a similar molecular weight distribution to TCC-PS_6.3k_ (*M*_n_ = 23000 g/mol, *Đ* = 2.12), but no TCC functionalities yielded a marginal shear strength of 0.63 ± 0.14 MPa, only (Figure 3a). Pure PS suffered from clear adhesive failure, as expected from a high *T*_g_, brittle polymer.

**Figure 3:**
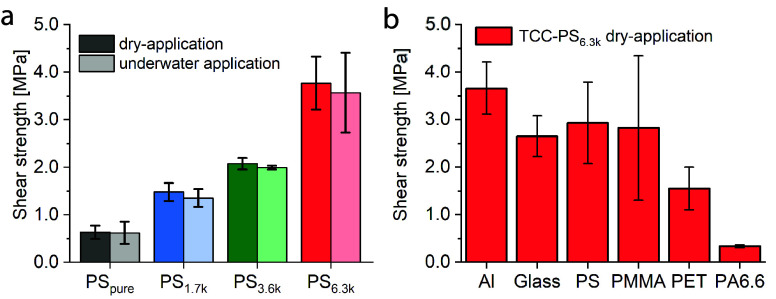
Lap shear tests of the TCC-PS polymers on aluminum resulted in a bonding strength of up to 3.8 MPa, an increase of 600% compared to pure PS (a). The glue was also tested on different materials and showed highest results on chemically similar surfaces (b).

It can be assumed that multiple factors contribute to the bond strength and fracture profiles, as evident by the comparison of TCC-PS_1.7k_ and TCC-PS_6.3k_. Despite containing only 3 mol % TCC functionalities, TCC-PS_6.3k_ achieved the highest bonding strength and exhibited a mixed cohesive failure mode (Figures 4 and S49). Whereas the TCC-rich TCC-PS_1.7k_ reached only 40% of the shear strength of TCC-PS_6.3k_ and failed in a clearly adhesive manner.

**Figure 4:**
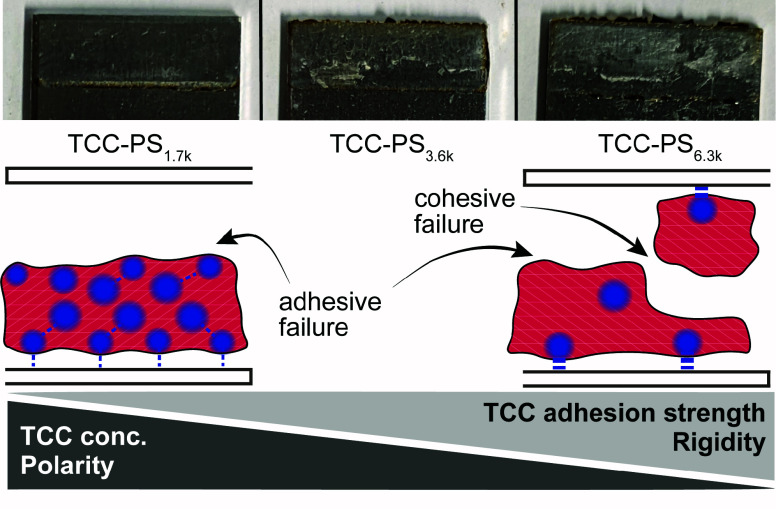
Fracture pattern of bonding on aluminum specimen indicate overlaying factors that contribute to bond strength of the glue. TCC-PS_1.7k_ proved a purely adhesive break, suggesting weaker binding of TCCs. Whereas TCC-PS_6.3k_, with low TCC concentration evidenced mixed cohesive failure that suggests stronger binding of TCCs in the more hydrophobic and rigid environment.

Apparently, the adhesion strength to the substrate is not only defined by the TCC concentration, but also by the embedding polymer matrix, where undisturbed PS-segments build up cohesion by mechanical entanglements^32^ and van der Waals forces, creating glassy hydrophobic regions. Remarkably, even a minor amount of TCC functionalities, as present in TCC-PS_6.3k_, significantly enhances the adhesive interface without drastically affecting the bulk properties, as evidenced by the smallest reduction in *T*_g_ to 92 °C. As a result, the adhesive failure mode of pure PS changed to a mixed cohesive failure, while the bond strength increased dramatically by 600%.

A high concentration of TCC functionalities in TCC-PS_1.7k_ reduces the overall bond performance and leads to adhesive failure. This can be rationalized by the fact, that TCC functionalities increase the polarity and introduce structural defects of the bulk matrix (*T*_g,TCC-PS1.7k_ = 86 °C). Obviously, both changes negatively affect the adhesive interface, where the binding capabilities of the presented TCC functionalities are expected to be reduced in a more polar and less rigid environment that probably causes a lowering of binding enthalpy and increasing entropic penalty.

In addition to bonding aluminum substrates, the applicability of the best performing TCC-PS_6.3k_ adhesive was tested on other materials as well. The application procedure was kept similar, but a lower curing temperature of 60 °C and an increased curing time of 3 days were used due to the sensitivity of some specimens (Figure 3b). The adhesive failed to bond oak wood substrates in the standard application format due to the absorbent surface. To glue such substrates, adhesives with different viscosities must be used. This would therefore require formulation, which is beyond the scope of this study. Glass substrates, however, could be bonded with TCC-PS_6.3k_ adhesives well and achieved a strength of 2.65 ± 0.44 MPa. Among the set of polymer substrates tested, the adhesive strength was highest for bonding at 2.93 ± 0.86 MPa, followed closely by poly(methyl methacrylate) (PMMA) at 2.83 ± 1.52 MPa and poly(ethylene terephthalate) (PET) at 1.55 ± 0.45 MPa. Polyamide 6.6 (PA6.6) exhibited the weakest results, with an adhesive strength of 0.34 ± 0.03 MPa.

These observations align rather well with the variations in the Hildebrand solubility parameters, which suggest improved compatibility between glues and substrates having similar polarity (δ_PS_ = 22.5 MPa^1/2^, δ_PMMA_ = 22.7 MPa^1/2^, δ_PET_ = 21.9 MPa^1/2^, δ_PA6.6_ = 23.4 MPa^1/2^).^33^ Hence, on the moderately less polar PET (Δ(δ_PS_-δ_PET_) ≈ 0.6), the glue showed better results than on the more polar PA6.6 (Δ(δ_PA6.6_-δ_PS_) ≈ 0.9), where the glue was not forming strong bonds. However, surface and glue compatibility are also affected by the surface energy of the substrates.^6^ Adhesives can wet and spread more easily on high-energy surfaces like aluminum or glass, resulting in a homogeneous adhesive film and adhesive interfaces, which contribute to bond strength.^34^ Most plastics exhibit lower surface energy, making them more challenging to glue.^35^

Inspired by mussel adhesives, the robustness of the glue toward water-wetted aluminum substrates was studied, using protocols adapted from Wilker and co-workers.^36^ The TCC-PS_*x*_ glues were dissolved in chloroform, applied under water onto aluminum specimens, and covered with a second specimen. The system was kept under water at 25 °C for 7 days and conditioned at ambient temperature for 3 days prior to lap shear testing. The bonding strength of the adhesive applied on wet surfaces was not significantly reduced compared to that of the application under dry conditions (Figure 3a). The most promising candidate TCC-PS_6.3k_ reached with 3.57 ± 0.84 MPa 95% of the strength achieved in the dry experiment. TCC-PS_1.7k_ and TCC-PS_3.6k_ resulted in 1.35 ± 0.19 and 1.99 ± 0.04 MPa, respectively, recovering 92–96% of the dry adhesive strength. As anticipated, pure PS suffered also under the given conditions from the lack of TCC functionalities and achieved an adhesive strength of 0.62 ± 0.23 MPa.

In summary, three telechelic polystyrene (PS) polymers with varying molecular weights were synthesized by using RAFT polymerization and subsequent thiol deprotection. The α,ω-dithiol PS segments reacted cleanly with bisquinone A (BQA) via a thiol-quinone Michael-polyaddition to generate thiol-catechol connectivities (TCCs) as potent adhesive functionalities. The resulting segmented block copolymers reached molecular weights of *M*_w,app_ = 14700–55800 g/mol and dispersities of *Đ* = 1.6–2.4.

These copolymers were then applied to aluminum specimens under dry conditions. The introduction of adhesive TCC functionalities at concentrations of 3–13 mol % dramatically improved the adhesive performance from 0.6 MPa of nonfunctional PS to up to 3.8 MPa. Additionally, the glue maintained its strength when applied underwater to aluminum surfaces. The adhesive TCC-PS_6.3k_ with PS block lengths of *M*_n_ = 6300 and 3.3 mol % TCCs performed best, showing compatibility with a range of other substrates, including PS (2.9 MPa), PMMA (2.8 MPa), glass (2.7 MPa), and PET (1.6 MPa), but failing at PA6.6 (0.3 MPa) and oak wood (no adhesion). The results followed well the variations in the Hildebrand solubility parameters. While PS was used to demonstrate compatibility with the polyaddition mechanism, RAFT offers means for the controlled synthesis of various telechelic commodity polymers to improve adhesive performance for expanding applications.

## Supporting Information Available

Experimental procedures and analytical data (PDF)
