# Supplementary material for: Enhancing Adhesion Properties of Commodity Polymers through Thiol-Catechol Connectivities: A Case Study on Polymerizing Polystyrene-Telechelics via Thiol-Quinone Michael-Polyaddition
Source: ACS Macro Lett. 2024 Mar 28;13(4):440–5. doi: 10.1021/acsmacrolett.4c00069 (PMC11025132; doi:10.1021/acsmacrolett.4c00069)
Supplement: Supplementary file 1 — mz4c00069_si_001.pdf [file mz4c00069_si_001.pdf]

Supporting Information (SI)

Enhancing adhesion properties of commodity polymers through thiol-catechol connectivities: A case study on polymerizing polystyrene-telechelics via thiol-quinone Michael-polyaddition.

Carolin M. Schröter, Lukas D. Bangert, Hans G. Börner*

Humboldt-Universität zu Berlin, Department of Chemistry, Laboratory for Organic Synthesis of Functional Systems, Unter den Linden 6, 10099 Berlin, Germany.

To whom correspondence should be addressed:

Prof. Dr. Hans G. Börner
Humboldt-Universität zu Berlin
Department of Chemistry
Laboratory for Organic Synthesis of Functional Systems

Brook-Taylor-Str. 2, Room 2.115, D-12489 Berlin, Germany

Phone: +49 (0)30-2093 7348

Fax: +49 (0)30-2093-7500

E-Mail: h.boerner@hu-berlin.de

Table of Contents

Materials and Instruments 3
Materials 3
Instruments 3
Methods 5
Sample Preparation for SEC analysis 5
Lap Shear Test 5
^{31}P NMR Sample Preparation 5
Synthesis 6
Synthesis of 2-iodoxybenzoic acid (IBX)[1] 6
Synthesis of bisquinone A (BQA)[2] 8
Synthesis of *S*-1-dodecyl-*S*‘-(α,α‘-dimethyl-α‘ ‘-acetic acid)trithiocarbonate (DDMAT)[3] 10
Synthesis of the bifunctional RAFT CTA (DiCTA)[4] 12
Synthesis of DiCTA-PS_x polymers 14
DiCTA-$PS_{1.7k}$ polymer 14
DiCTA-$PS_{3.6k}$ polymer 18
DiCTA-$PS_{6.3k}$ polymer 20
Further characterization 21
Synthesis of Dithiol-PS_x polymers[5] 21
Dithiol-$PS_{1.7k}$ polymer 22
Dithiol-$PS_{6.3k}$ polymer 26
Further Dithiol-PS_x characterization 28
Synthesis of TCC-$PS_{1.7k}$/TCC-$PS_{3.6k}$ model systems 28
Characterization TCC-$PS_{1.7k}$ model system 30
Characterization TCC-$PS_{3.6k}$ model system 33
Synthesis of TCC-PS_x polymers[2] 36
TCC-$PS_{1.7k}$ polymer 36
TCC-$PS_{3.6k}$ polymer 38
TCC-$PS_{6.3k}$ polymer 42
Further TCC-PS characterization 44
Total overview over synthesized polymers 47
Reaction analysis 48
Reaction optimization 48
Stochiometric optimization 48
Temperature optimization 50
Concentration optimization 50
SEC polymerization kinetics of each Dithiol-PS_x with BQA 51
SEC – polymerization overview 52
Synthesis of pure polystyrene as reference sample 53
Adhesion tests 54
Shear test results 54
Pure PS 54
Aluminum dry 55
Other materials 57
Aluminum under water 57
References 59

Materials and Instruments

Materials

Aluminum oxide activated (acidic), aluminum oxide activated (basic), butylated hydroxytoluene (BHT, ≥ 99.9 %), chromium(III) acetylacetonate (97 %), 4-dimethylaminopyridine (98%), 1-dodecanethiol (> 97%), methyl iodide (99%), azobisisobutyronitrile (98%), iron(III) chloride (97%), 2-iodobenzoic acid (> 98%), Oxone® (monopersulfate compound), styrene (≥ 99.9%), tributylphosphine (97%), 4-*tert*-butylbenzyl mercaptan (97%), trans-2-[3-(4-tert-butylphenyl)-2-methyl-2-propenylidene]malononitrile (DCTB, > 99.0%) were purchased from *Sigma Aldrich® Chemie GmbH* (Seelze, Germany). 2-Chloro-1,3,2-dioxaphospholane (97%), bisphenol A (> 99%), dibenzoyl peroxide (75%), ethanethiol (> 98%), 1-ethyl-3-(3-dimethylaminopropyl)carbodiimide hydrochloride (EDC-HCl) (98%), hexylamine (99%) were purchased from *TCI* (Tokyo, Japan). Carbon disulphide (99.9%), ethylene glycol (99.8%), *N*-Methyl-*N*,*N*,*N*-trioctylammonium chloride (Aliquat 336) (> 97%) pyridin anhydrous (> 99.5%), silver trifluoaroacetate (AgTFA, 98%) were purchased from *Fisher Scientific GmbH* (Schwerte, Germany). Sodium chloride (> 98%), ammonium solution (≥ 25%) were purchased from *Carl Roth GmbH & Co. KG* (Karlsruhe, Germany). Hydrochloric acid (37%), sodium hydroxide (99%), sodium sulphate (99%) were purchased from *Grüssing GmbH* (Filsum, Germany). *N*-Methyl-2-pyrrolidon (NMP peptide grade, free amines <10 ppm) was purchased from *Iris Biotech GmbH* (Marktredwitz, Germany). Tetrahydrofuran (HPLC grade) and *N*,*N*- Dimethylformamide (HPLC grade) were obtained from *VWR® chemicals* (Dresden, Germany). Deuterated dimethyl sulfoxide (DMSO-d6), deuterated chloroform ($CDCl_3$) and deuterated dichloromethane (CD_2Cl_2) was obtained from *Deutero GmbH* (Kastellaun, Germany).

Milli-Q water was produced using SG LaboStar ® TM 1-UV system from SG water (Hamburg, Germany). As ion exchanger, Evoqua Water Technologies Polisher HP2 module was inserted. Electric conductibility of Milli-Q water was 0.055 µS cm.

For lap shear experiments aluminum plates (5005A, 80 × 25 × 1.5 mm with one-sided bore) were purchased from ROCHOLL GmbH (Eschelbronn, Germany). The test specimens used are made of an aluminum-magnesium alloy and are frequently applied in standard tests.

Instruments

Ultra-performance liquid chromatography with electron spray ionization mass spectrometry (UPLC-ESI-MS) was carried out on an ACUIDITY-UPLC® H-Class CM Core System of Waters GmbH (Eschborn, Germany). Detection was done utilizing an ACUIDITY-UPLC® photo diode array (PDA)-detector (wavelength range 190-500 nm) and an ACUIDITY-UPLC® QDa mass detector with ESI-ionization. For analysis Waters software Empower TM 3 was

used. Separation was conducted with ACUIDITY-UPLC® BEH C18 VanGuard TM precolumn (110 Å, 1.7 µm, 5 × 21 mm ID) and an ACUIDITY-UPLC® BEH C18-column (110 Å, 1.7 µm, 5×21 mm ID) from Waters. As mobile phase, mixtures of solvent A (Milli-Q water with 0.1% FA, v/v) and solvent B (acetonitrile 0.1%FA, v/v) were used with 0.5 mL min -1 flow rates.

Gel permeation chromatography (GPC) was carried out on an Eco-SEC-System with UV and RI-detection (HLC-8320 GPC) from Tosoh (Griesheim, Germany). As solvent tetrahydrofuran (THF, HiPerSolv CHROMANORM ® for HPLC) from VWR® Chemicals (Dresden, Germany) was used and SDV columns (1000 Å 5 µm, 100000 Å 5 µm and 1000000 Å 5 µm) from PSS (Mainz, Germany) were applied.

Matrix-Assisted Laser Desorption/Ionization Time-of-Flight Mass Spectrometry (MALDI-TOF-MS): The coupling products were characterized by MALDI-ToF-MS performed on an Autoflex III Smartbeam system (Bruker Daltonik GmbH, Bremen) equipped with a Smartbeam laser (355 nm, 200 Hz working frequency). Detection of signals was performed with a Time-of-Flight detector and a voltage of 20 kV. Spectra were evaluated by using the software FlexControl 1.3. As matrix DCTB (20 mg/mL) and AgTFA (2 mg/mL) in THF was used and mixed with the sample (5 mg/mL) in a ratio of 10:1:4.

Nuclear magnetic resonance spectroscopy (NMR) was performed on an Avance II 500 spectrometer (*Bruker BioSpin GmbH*, Rheinstetten, Germany).

Fourier transform infrared spectroscopy (FT-IR) was carried out on a Vertex 70v FT-IR spectrometer (*Bruker Optik GmbH*, Ettlingen, Germany).

UV-visible spectroscopy (UV/vis) measurements were performed on a Spectrometer UV-2501PC from Shimadzu , Germany. The recorded spectra ranged from 200 to 800 nm. For UV/Vis sample preparation, BQA was solved in DMF at a concentration of 0.04 mg/mL.

"Differential Scanning Calorimetry (DSC) measurements were conducted on an EXSTAR DSC 7020 (*SII Nanotechnology Inc., Tokyo, Japan*) with the software “Muse Measurement” and “Muse Standard Analysis”, version 9.3 (*Hitachi High-Tech Science Corp., Tokyo, Japan*).

All glue mixtures were homogenized with a SpeedMixer DAC 150 SP (*Hauschild, Germany*).

All specimens were cleaned with ozone for 15 min using UVC-1014 (*NanoBioAnalytics, Bürgel, Germany*) prior to every shear test.

Shear tests have been carried out with a Texture Analyzer Ta.XT.plus100C (*Stable Micro Systems, Godalming, United Kingdom*) with a 100 kg force cell.

Scanning Electron Microscopy (SEM) was carried out using a Tabletop Microsope TM-1000 (*Hitachi, Tokyo, Japan*).

Methods

Sample Preparation for SEC analysis

The dried polymer solid was dissolved in THF and filtered through 0.2 µm PTFE filter. 1.5 mL of the filtrated solution were transferred into glass vials and mixed with 7.5 µL of BHT-standard (50 mg/mL solution in THF).

For kinetic studies, the reaction was quenched with *n*butyl acrylate addition.

For disulfide measurements, 1 drop of tributyl phosphine was added and mixture was stirred 24 h prior to injection.

Lap Shear Test

Force-distance-curves were measured in a shear arrangement with a velocity of 0.05 mm/s. The adhesion strength was calculated by normalizing the maximum force of the force-distance-curve to the area of the adhesive overlap of the test specimen.

^{31}P NMR Sample Preparation

For the ^{31}P-NMR measurement, the polymer was derivatized with 2-chloro-2-oxo-1,3,2-dioxaphospholane (CDP) using $Cr(acac)_3$ and Ph_3PO as internal standard in pyridine:$CDCl_3$-1.6:1 v/v% at ambient temperature for 15 min.

As reference, Ph_3PO (c = 1.2 mol/L, n = 0.01 mmol per measurement) was set at δ = 26.84 ppm. Phenolic hydroxy group signals can be seen between δ = 133.7 – 127.7 ppm.

For TCC-$PS_{1.7k}$: M_n(TCC-$PS_{1.7k}$) = 9100 g/mol, n(TCC-$PS_{1.7k}$)/tube = 0.00465 mmol,
integral phenolic OH groups/tube = 9.37 → n(ph. OH)/tube = 0.0937 mmol,
n(ph.OH)/polymer chain = n(ph.OH)/tube / n(TCC-$PS_{1.7k}$)/tube = 20.30 1/chain,
1 ph. OH ≙ 0.5 catechol moieties ≙ 0.25 BQA blocks
→ in average 5.1 BQA incorporated per TCC-$PS_{1.7k}$ chain.

Synthesis

Synthesis of 2-iodoxybenzoic acid (IBX)[1]

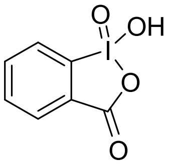

20.0 g 2-Iodobenzoic acid (0.081 mol, 1.0 eq.) were added to a solution of 73.5 g Oxone (0.120 mol, 1.5 eq.) in 260 mL deionized water in a 500 mL flask. The reaction mixture was warmed to 73 °C for 3 h and subsequently stirred in an ice bath at 5 °C for 30 min for precipitation. The mixture was filtered and the solid washed six times with 50 mL of water and two times with 50 mL of acetone. A white solid with a yield of 82.0 % was obtained.

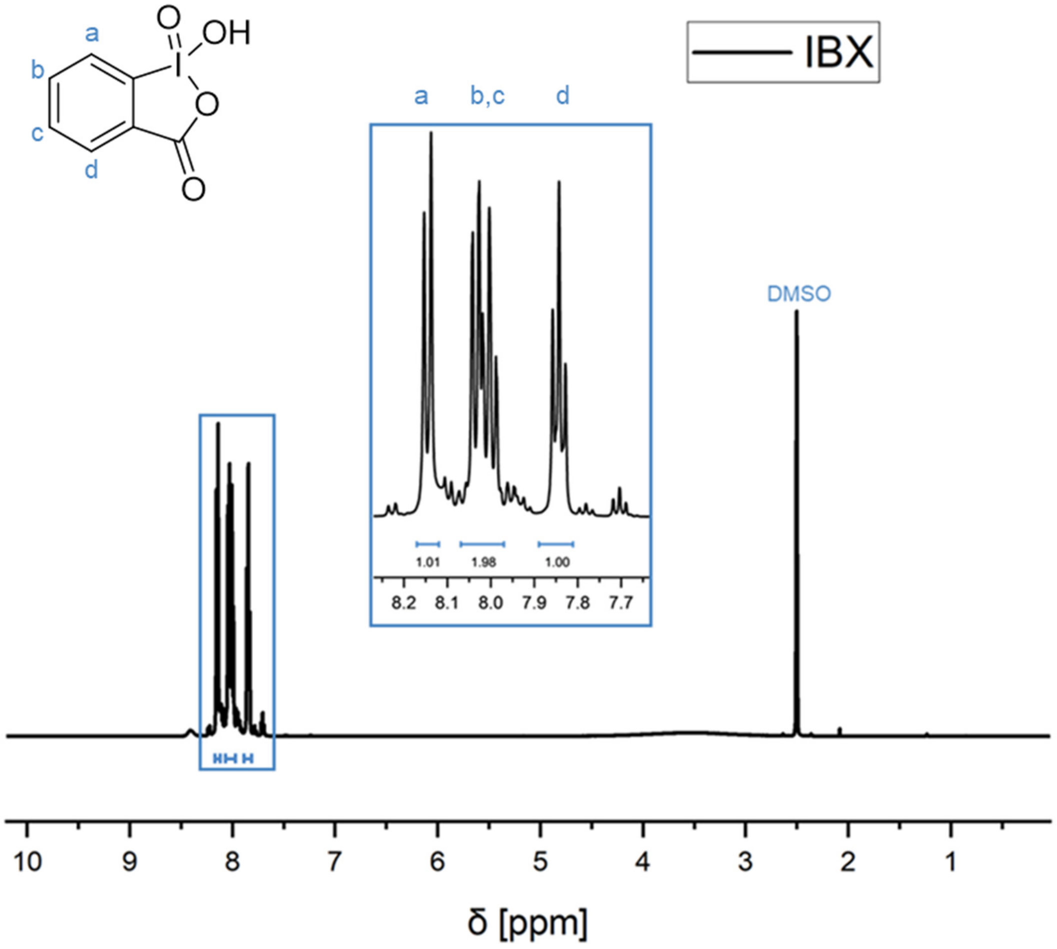

Figure S1. ^{1}H-NMR of 2-iodoxybenzoic acid (IBX).

^{1}H-NMR: (500 MHz, DMSO-d6, δ in ppm): 8.14 (d, J = 8.15 Hz, 1H), 8.04 – 7.99 (m, 2 H), 7.84 (t, J = 7.84 Hz, 1H).

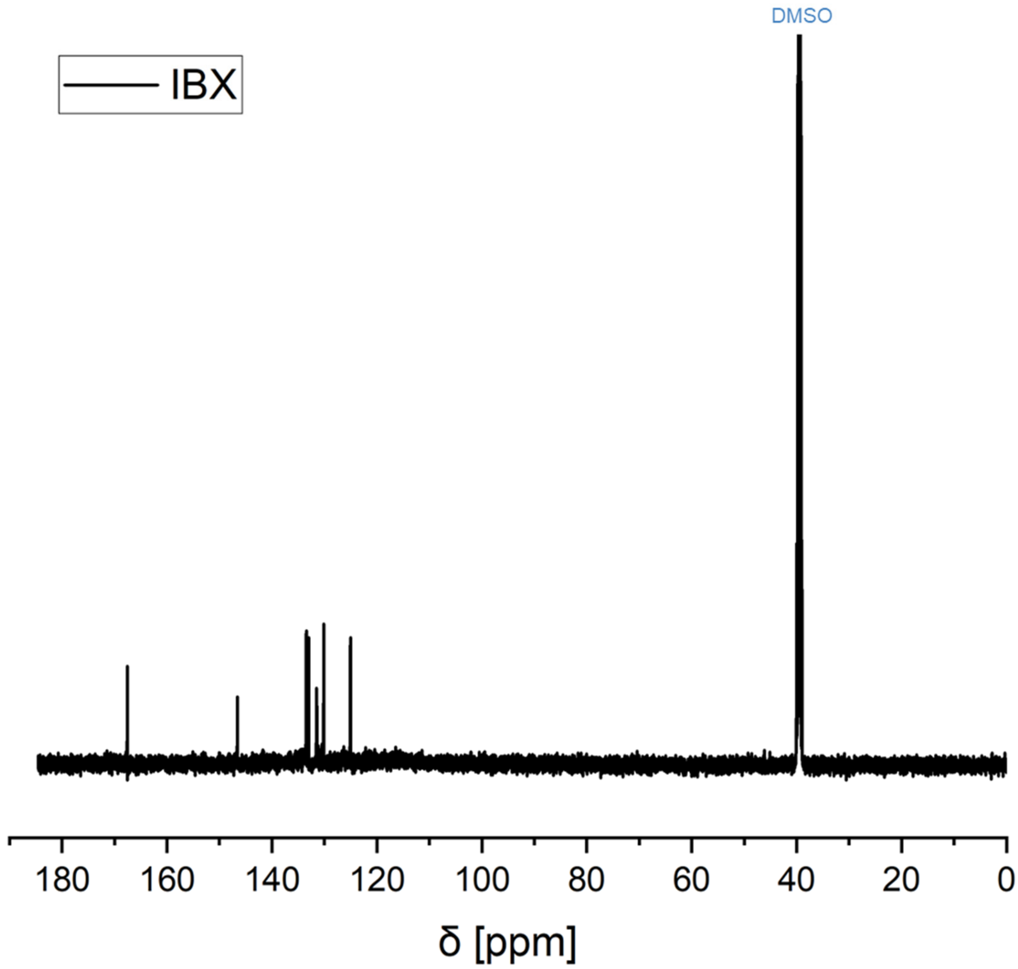

Figure S2. ^{13}C NMR of 2-iodoxybenzoic acid (IBX).

^{13}C-NMR: (500 MHz, DMSO-d6, δ in ppm): 167.6, 146.6, 133.5, 133.0, 131.5, 130.1, 125.0.

Synthesis of bisquinone A (BQA)[2]

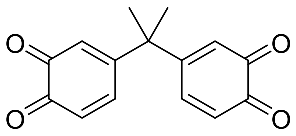

To 18.9 g IBX (0.067 mol, 3.0 eq) in a 500 mL flask was added a solution of 5.0 g bisphenol A (0.022 mol, 1.0 eq) in 200 mL methanol. The mixture was stirred for 15 min at ambient temperature at which the solution turned red. After stirring at 0 °C for further 10 min, a solid precipitated. The mixture was filtered, the solid was washed with cold methanol and subsequently redissolved in 750 mL of chloroform. The mixture was filtered again, and the mother liquid was evaporated under reduced pressure to obtain the crystalline red product with a yield of 89.0 %.

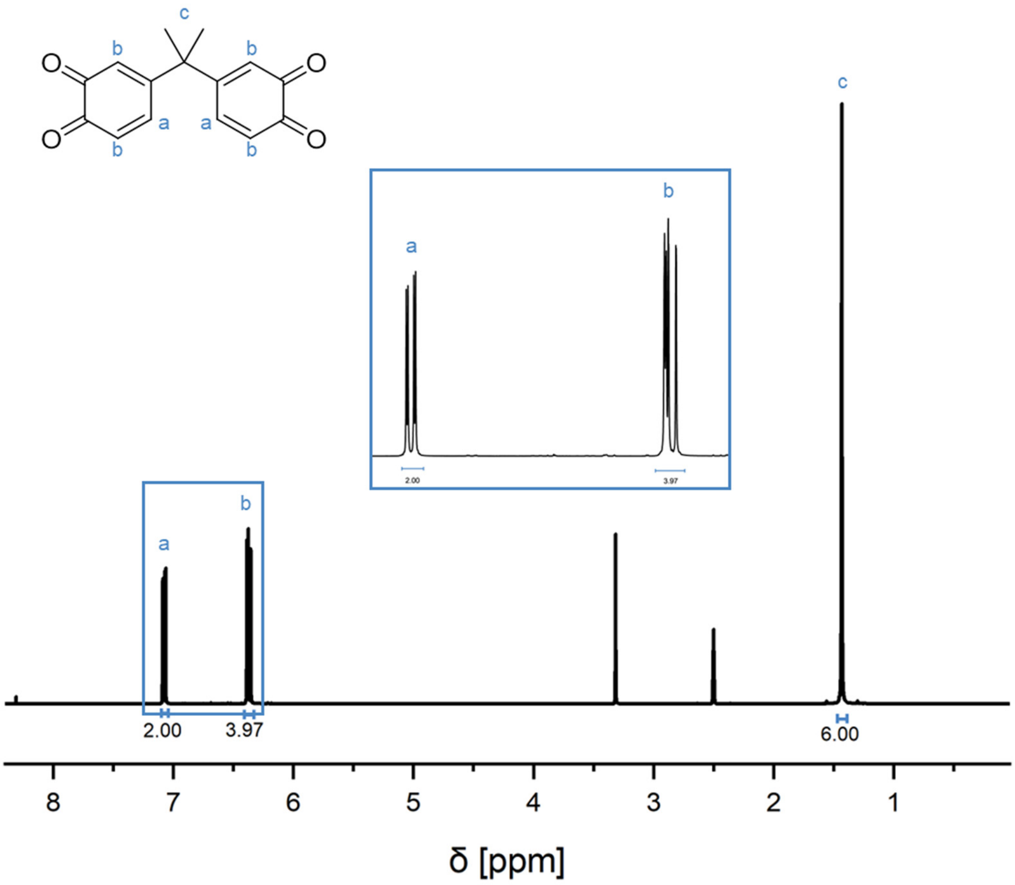

Figure S3. ^{1}H-NMR of bisquinone A (BQA).

^{1}H-NMR: (500 MHz, DMSO-d6, δ in ppm): 7.06 (dd, J = 10.4, 2.3 Hz, 2H), 6.38 – 6.35 (m, 4H), 1.43 (s, 6H).

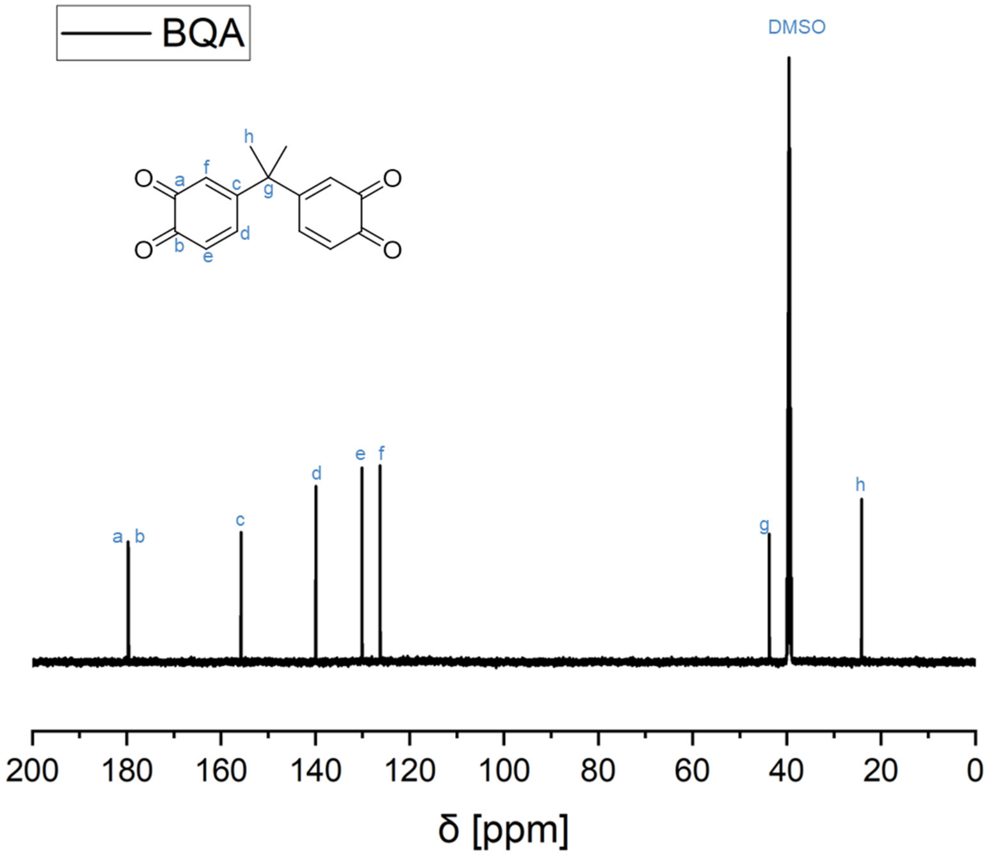

Figure S4. ^{13}C-NMR of bisquinone A (BQA).

^{13}C-NMR: (500 MHz, DMSO-d6, δ in ppm): 179.7, 179.6, 155.8, 139.9, 130.1, 126.3, 43.7, 24.2.

FTIR [cm^{-1}]: 3300, 2978, 1684, 1664, 1639, 1622, 1566, 1468, 1406, 1371, 1302, 1286, 1236, 1180, 1149, 1139, 1128, 1040, 914, 876, 816, 764, 743, 727, 683, 646, 615, 590.

Synthesis of *S*-1-dodecyl-*S*'-(α,α'-dimethyl-α''-acetic acid)trithiocarbonate (DDMAT)[3]

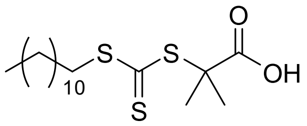

The RAFT chain transfer agent was synthesized according to Lai *et al.*. 80.76 g 1-Dodecanethiol (0.40 mol, 1.0 eq.), 200 mL acetone (192.4 g, 3.31 mol, x eq.) and 6.49 g Aliquat 336 (tricaprylylmethylammonium chloride, 0.016, 0.04 eq.) were added under stirring to a 2 L flask in an ice bath. Next, 33.54 g of 50 wt% sodium hydroxide solution (0.42 mol, x eq) was added over 20 min. After stirring for another 15 min, 30.42 g carbon disulfide (0.40 mol, 1.0 eq) in 40.36 g acetone (0.69 mol) was added over 10 min. The color turned from white to orange. After 10 min, 71.25 g chloroform (0.60 moll, 1.5 eq) was added in one part and subsequently, 160 g of 50 wt% sodium hydroxide solution (2 mol, x eq) was added dropwise over 30 min. The mixture was stirred overnight. The next day, 600 mL of water and 100 mL of concentrated HCl were added. Argon was purged through the mixture under stirring to evaporate off the acetone. The solid was filtered off and stirred in 1 L of 2-propanol. The mixture was filtered again and the liquid phase was evaporated under reduced pressure. The resulting solid was recrystallized from hexane to obtain yellow crystals in 60.6 % yield.

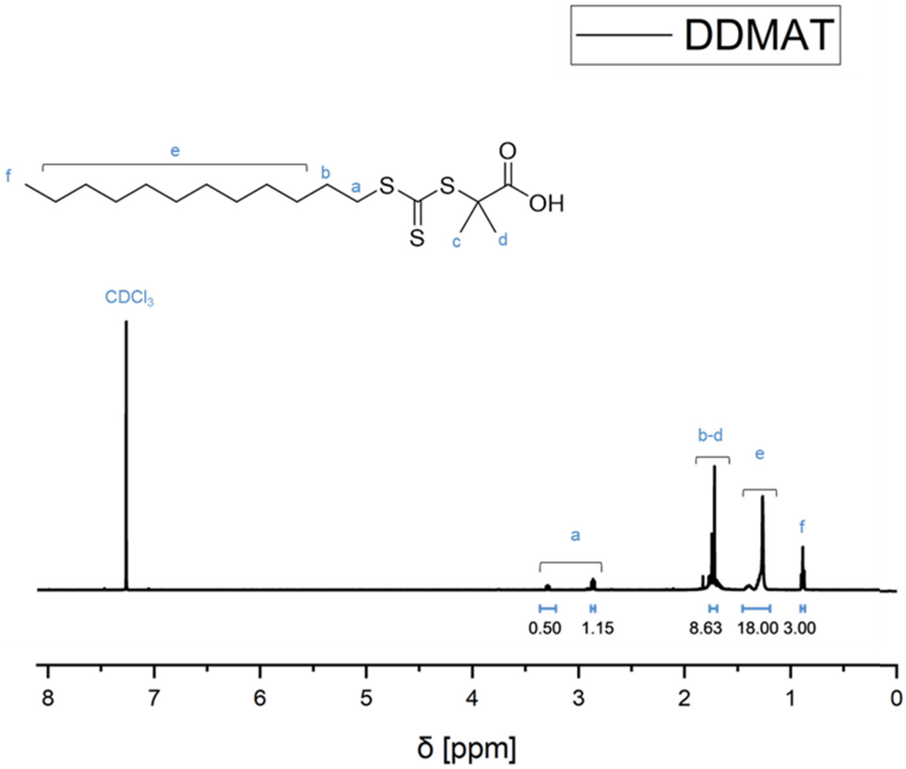

Figure S5. ^{1}H-NMR of S-1-dodecyl-S'-(α,α'-dimethyl-α''-acetic acid)trithiocarbonate (DDMAT).

^{1}H-NMR: (500 MHz, $CDCl_3$, δ in ppm): 3.29 (t, 1H), 2.86 (t, 1H), 1.80 – 1.64 (m, 8H), 1.42 – 1.26 (m, 18H), 0.88 (t, 3H).

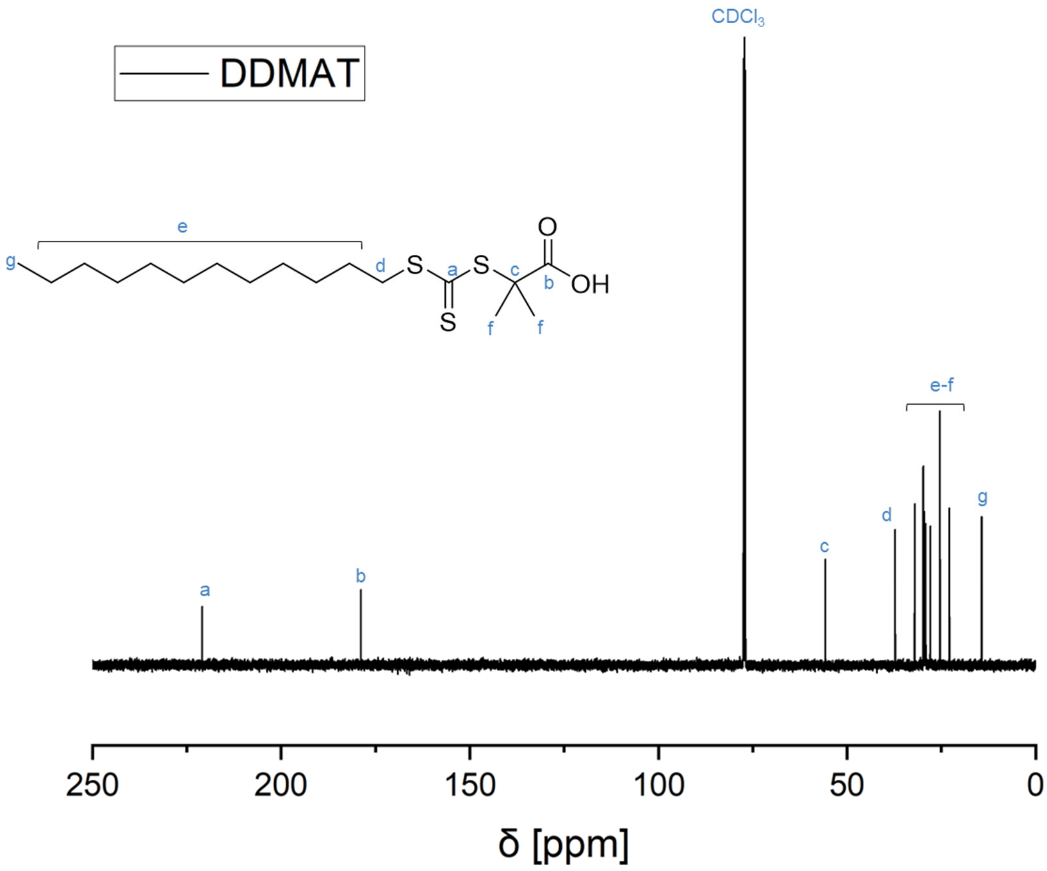

Figure S6. ^{13}C-NMR of S-1-dodecyl-S'-(α,α'-dimethyl-α''-acetic acid)trithiocarbonate (DDMAT).

^{13}C-NMR: (500 MHz, CDCl3, δ in ppm): 220.9, 178.8, 55.7, 37.2, 32.1, 29.8, 29.7, 29.6, 29.5, 29.3, 29.1, 27.9, 14.3.

FTIR [cm^{-1}]: 2956.7, 2918.1, 2850.6, 2651.9, 2542.0, 1699.2, 1458.1, 1436.9, 1413.7, 1380.9, 1367.4, 1282.6, 1168.8, 1128.2, 1064.6, 948.9, 914.2, 813.9, 721.3, 694.3, 470.6.

Synthesis of the bifunctional RAFT CTA (DiCTA)[4]

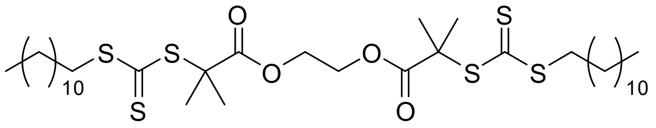

850 mg Ethylene glycol (13.7 mmol, 1.0 eq.), 838 mg 4-dimethylaminopyridine (DMAP, 6.86 mmol, 0.5 eq) and 5.65 g *N*-(3-dimethylaminopropyl)-*N′*-ethylcarbodiimide hydrochloride (EDC·HCl, 27.4 mmol, 2.0 eq) were solved in 500 mL dichloromethane. After stirring, 10.0 g DDMAT (27.4 mmol, 2.0 eq) were added. The solution was stirred over night at ambient temperature. The organic phase was washed twice with 1 M HCl, 5 wt% sodium hydroxide solution and saturated sodium chloride solution. After drying it over sodium sulphate, the solution was evaporated under reduced pressure and purified via silica column chromatography using cyclohexane and dichloromethane (v/v = 1:1). A yellow solid was obtained with a yield of 49.4 %.

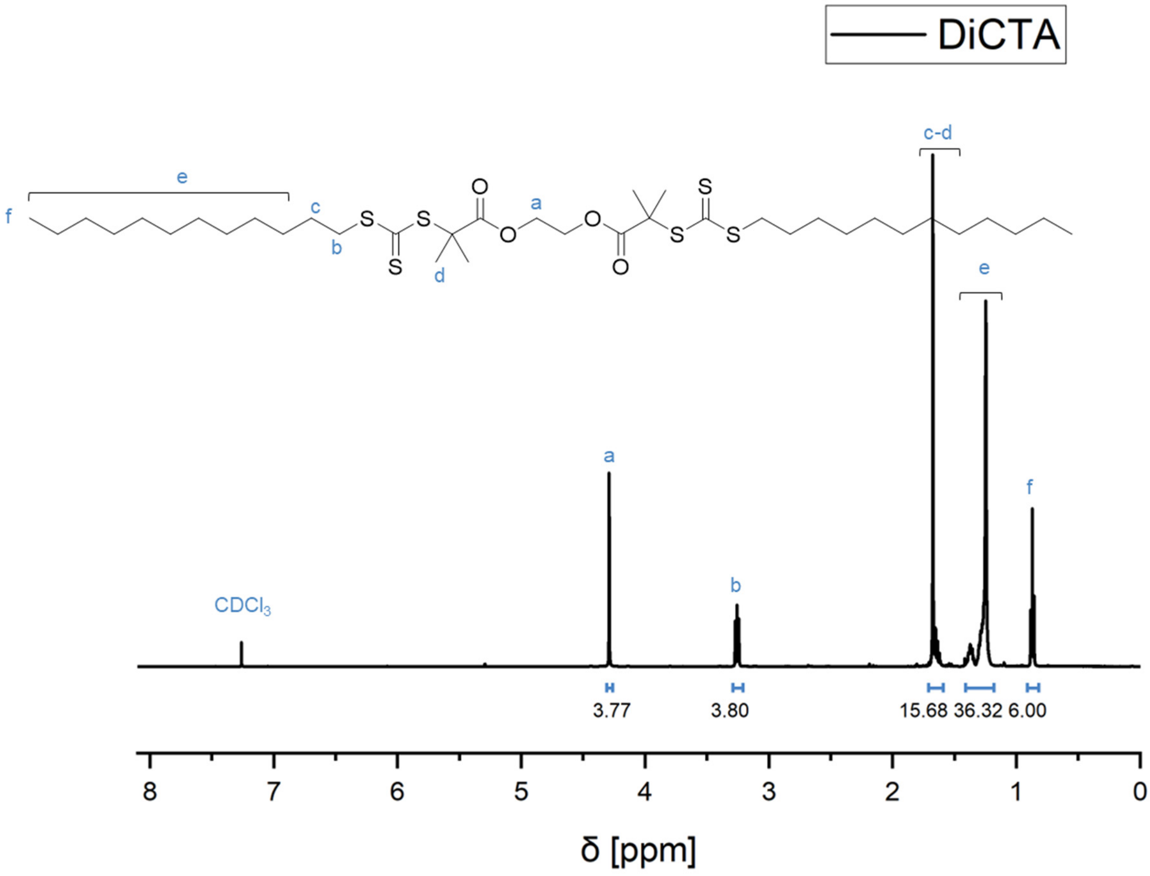

Figure S7. ^{1}H-NMR of bifunctional RAFT CTA (DiCTA).

^{1}H-NMR: (500 MHz, CDCl3, δ in ppm): 4.29 (s, 4H), 3.26 (t, 4H), 1.67 – 1.62 (m, 16H), 1.41 – 1.25 (m, 36H), 0.87 (t, 6H).

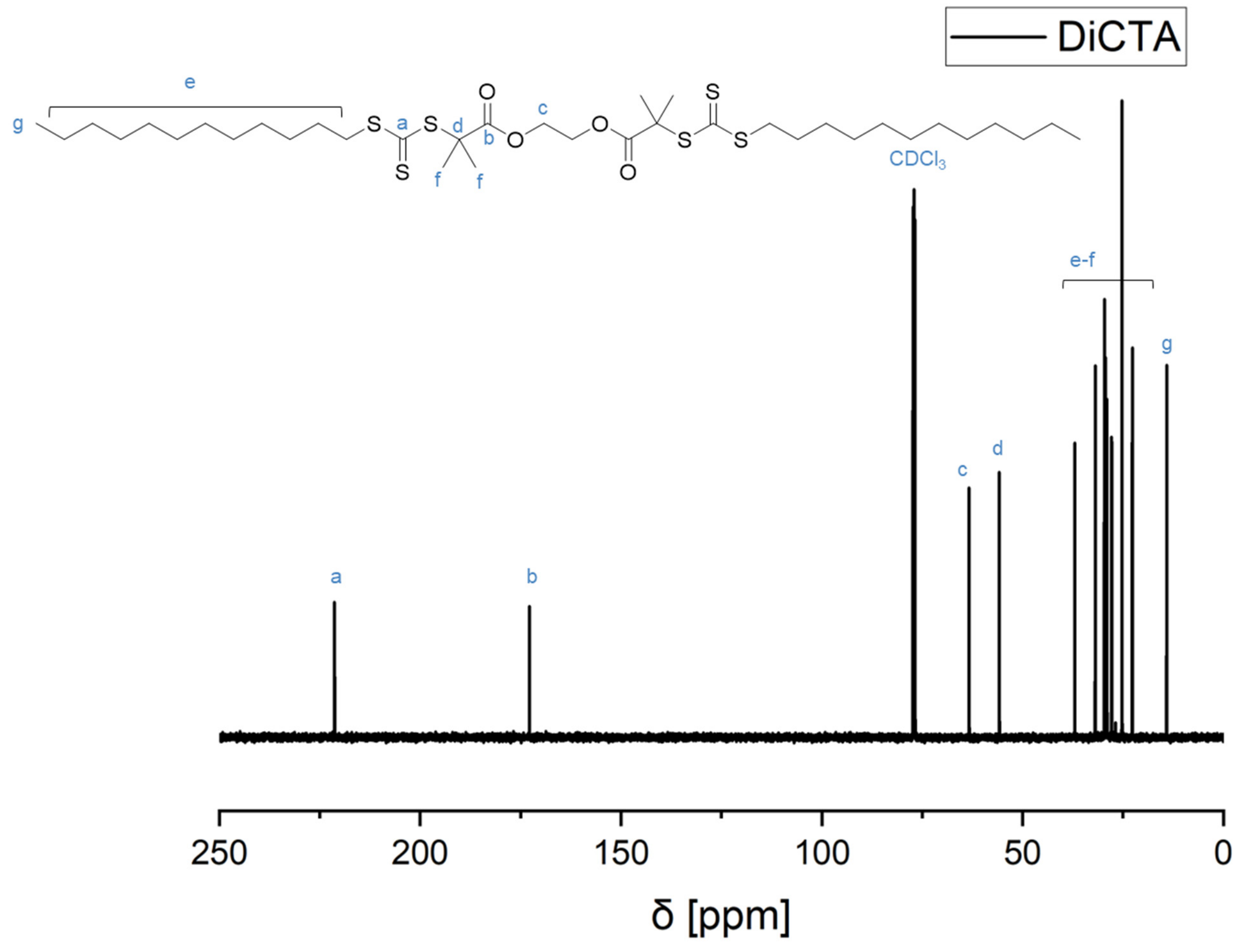

Figure S8. ^{13}C-NMR of bifunctional RAFT CTA (DiCTA).

^{13}C-NMR: (500 MHz, CDCl3, δ in ppm): 221.3, 172.8, 63.3, 55.8, 37.0, 31.9, 29.7, 29.7, 29.6, 29.5, 29.4, 29.1, 29.0, 27.9, 26.9, 25.3, 22.7, 14.2.

FTIR [cm^{-1}]: 2954.7, 2920.0, 2850.6, 1724.2, 1699.2, 1652.9, 1558.4, 1541.0, 1521.7, 1508.2, 1458.1, 1436.9,1271.0, 1145.6, 1124.4, 1066.6, 819.7, 623.0, 453.2.

Synthesis of DiCTA-PS_x polymers

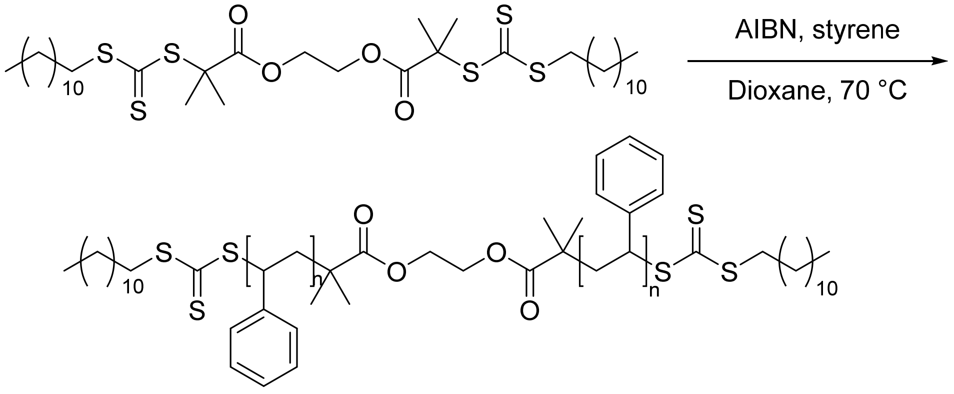

DiCTA-$PS_{1.7k}$ polymer

25.0 g styrene (240 mmol) was destabilized over basic aluminum oxide and dissolved in 50% dioxane. 3.63 g of bifunctional RAFT CTA (DiCTA) (4,80 mmol) and 78.8 mg of azobisisobutyronitrile (AIBN) (0.480 mmol) were added. The mixture was degassed with argon for 1 h in an ice bath at 5 °C. The reaction was started by stirring the mixture in an oil bath at 70 °C. The polymerization was stopped after 7 h by flooding the sample with oxygen. The polymer mixture was evaporated under reduced pressure, redissolved with tetrahydrofuran and precipitated in cold methanol.

The ratio of monomer : DiCTA : AIBN was 500 : 1 : 0.1

M_n = 2040 g/mol (SEC),

^{1}H-NMR ratio of CTA Z-group : CTA R-group : styrene = 1 : 1.2 : 13.9 → $M_{n,NMR}$ = 2203 g/mol

M_w = 2180 g/mol

M_p = 2220 g/mol

$Đ$ = 1.07

T_g = -5.8 °C

UV/Vis: λ_{max}(DMF)/nm 310.

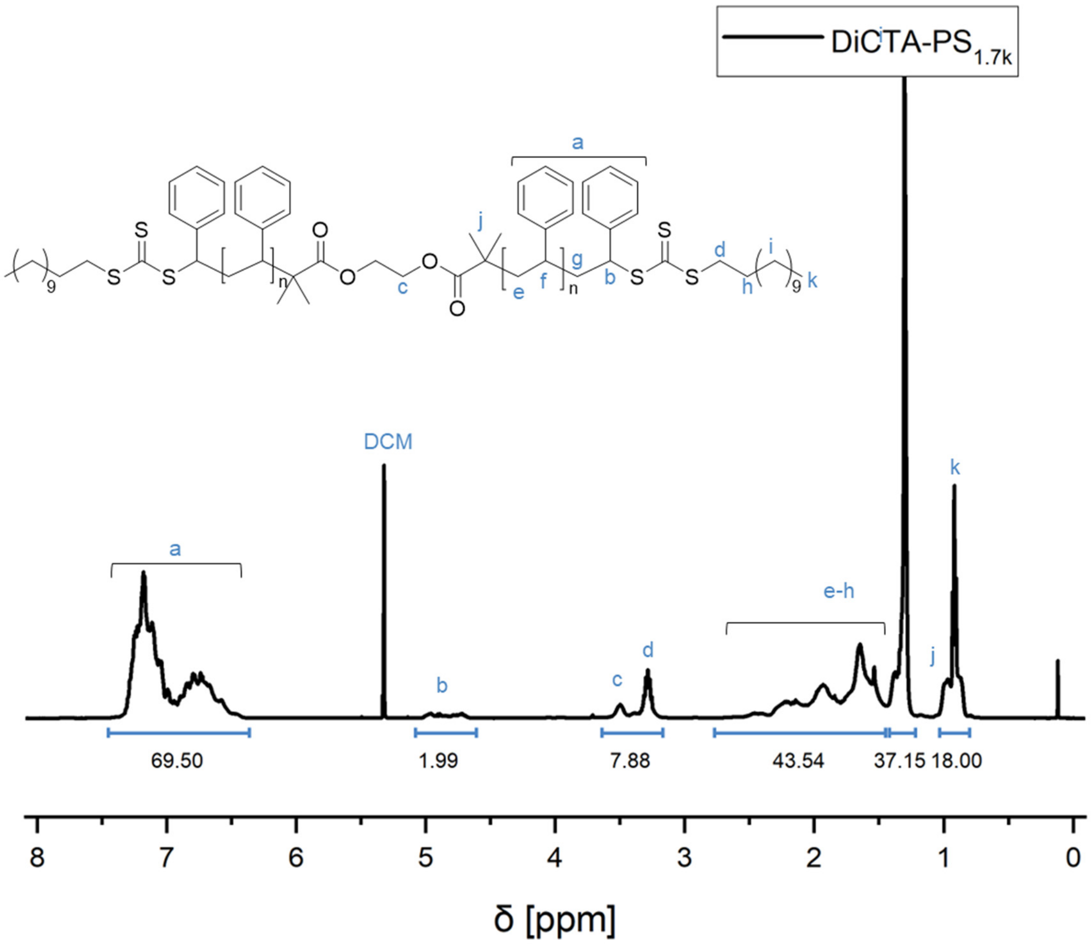

Figure S9. ^{1}H-NMR of DiCTA-PS$_{1.7k}$.

^{1}H-NMR: (500 MHz, CD_2Cl_2, δ in ppm): 7.28-6.45 (m, 70H, Ar-H), 4.99-4.68 (m, 2H), 3.50 (b, 2H), 3.28 (m, 4H), 2.69-1.46 (m, 44H), 1.38-1.27 (m, 36H), 0.99-0.87 (m, 18H).

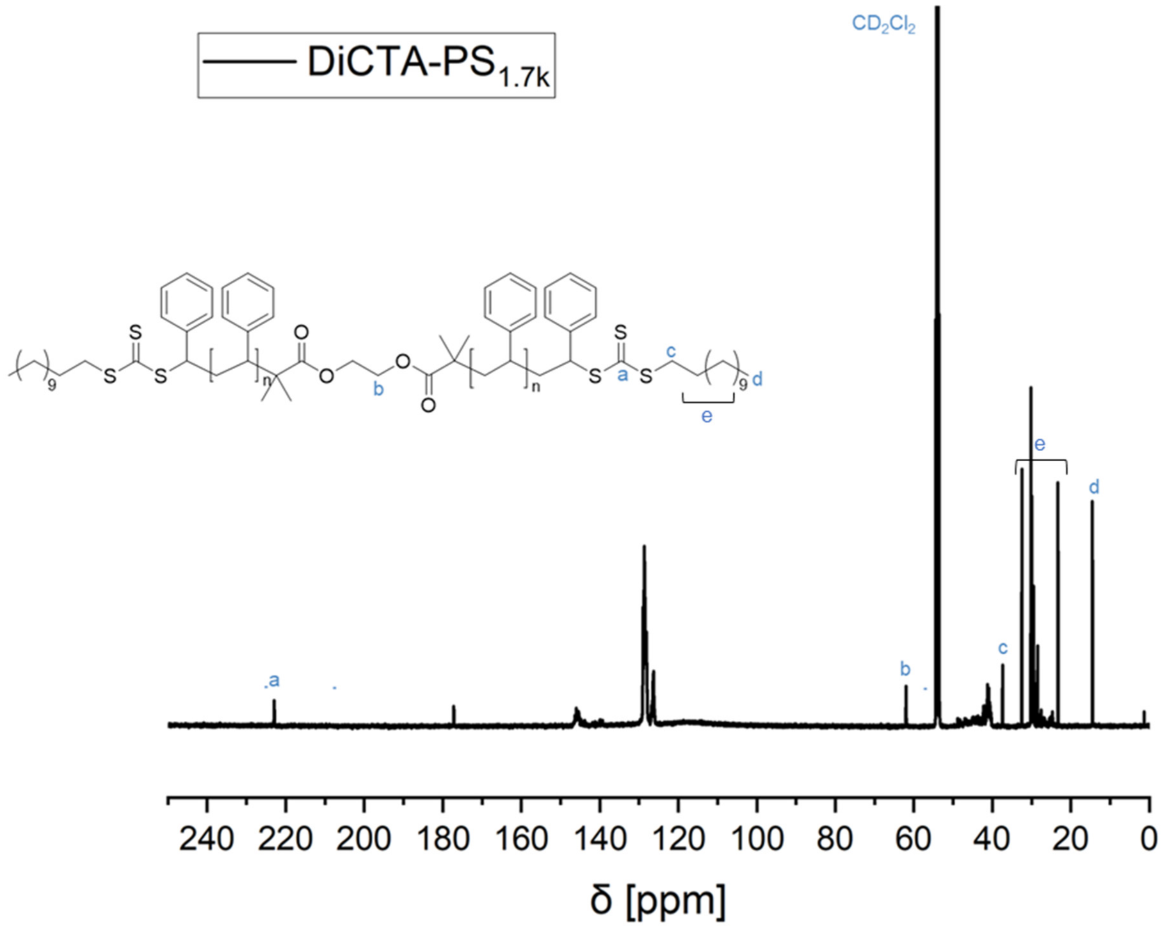

Figure S10. *^{13}C-NMR of DiCTA-PS$_{1.7k}$.*

^{13}C-NMR: (500 MHz, CD_2Cl_2, δ in ppm): 222.9, 177.2, 145.8, 128.7, 62.0, 48.9-40.9, 37.4, 32.2, 30.2-23.3, 14.5.

1: 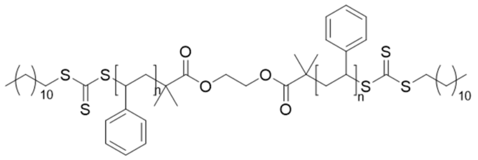

2: 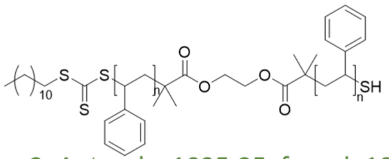

a: n=1, Ag^+, calc: 1071.36, found: 1071.75 m/z
b: n=4, Ag^+, calc: 1697.87, found: 1697.87 m/z
c: n=6, Ag^+, calc: 2112.99, found: 2113.96 m/z
d: n=8, Ag^+, calc: 2533.23, found: 2530.65 m/z

e: n=2, Ag^+, calc: 1035.35, found: 1036.70 m/z
f: n=4, Ag^+, calc: 1452.61, found: 1453.09 m/z
g: n=6, Ag^+, calc: 1868.86, found: 1870.00 m/z
h: n=8, Ag^+, calc: 2285.11, found: 2286.25 m/z

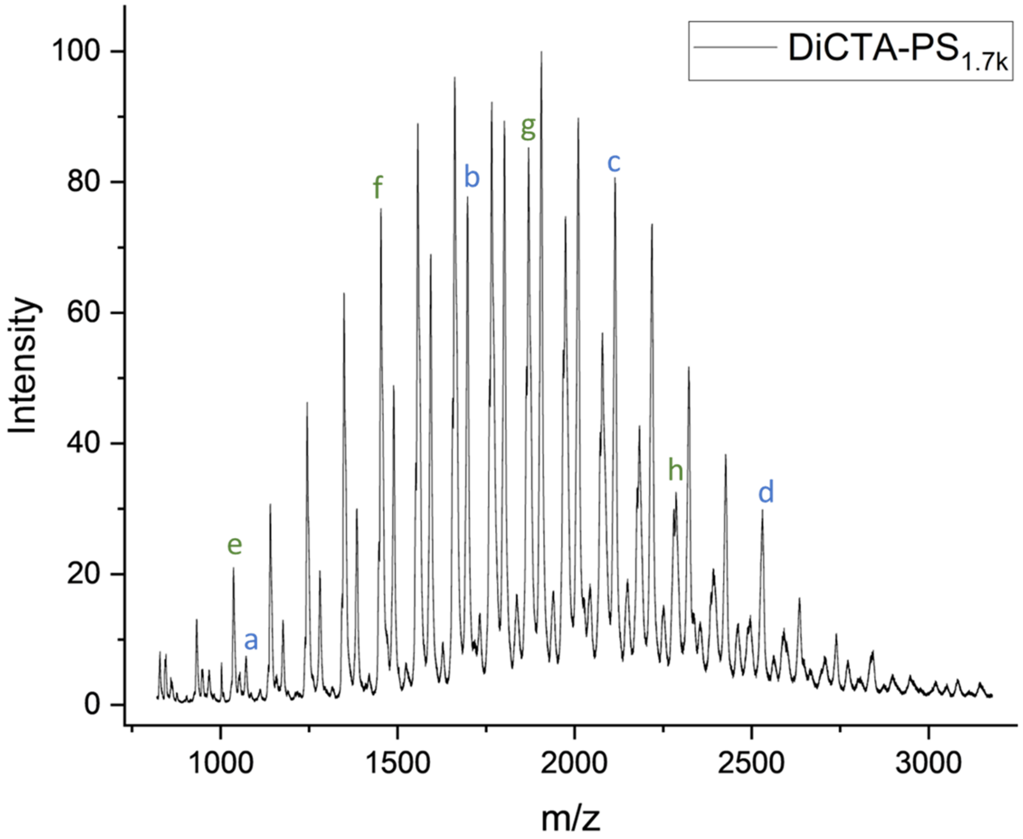

Figure S11. MALDI-ToF-MS spectrum of DiCTA-PS$_{1.7k}$ measured in linear positive mode with AgTFA. During MALDI-ToF-MS measurements, the laser power leads to fragmentations of the colored polymer.

MALDI-ToF-MS:

Structure 1: *m/z* 1071.75 (Ag^+, 8%), 1176.37 (13), 1280.45 (21), 1385.17 (30), 1488.81 (49), 1593.65 (69), 1697.87 (78), 1801.86 (89), 1906.27 (100), 2010.53 (90), 2113.96 (81), 2218.99 (74), 2322.43 (52), 2426.6 (38), 2530.65 (30), 2635.3 (16), 2739.45 (11), 2843.33 (8).

Structure 2: *m/z* 828.79 (Ag^+, 8%), 932.67 (13), 1036.7 (21), 1140.65 (31), 1244.75 (46), 1348.90 (63), 1453.09 (76), 1557.24 (89), 1661.63 (96), 1766.12 (92), 1870.00 (85), 1974.44 (75), 2078.8 (57), 2183.51 (43), 2286.25 (33), 2391.7 (21), 2496.56 (14), 2600.26 (9), 2705.21 (8), 2807.94 (4).

DiCTA-PS3.6k polymer

25.0 g styrene (240 mmol) was destabilized over basic aluminum oxide and dissolved in 50% dioxane. 3.63 g of bifunctional DiCTA agent (4.80 mmol) and 78.8 mg of AIBN (0.480 mmol) were added. The mixture was degassed with argon for 1 h in an ice bath at 5 °C. The reaction was started by stirring the mixture in an oil bath at 70 °C. The polymerization was stopped after 48 h by flooding the sample with oxygen. The polymer mixture was evaporated under reduced pressure, redissolved with tetrahydrofuran and precipitated in cold methanol.

The ratio of monomer : DiCTA : AIBN was 500 : 1 : 0.1

M_n = 3740 g/mol (SEC)

^{1}H-NMR ratio of CTA Z-group : CTA R-group : styrene = 1 : 1 : 33.8 → $M_{n,NMR}$ = 4277 g/mol

M_w = 4040 g/mol

M_p = 4100 g/mol

$Đ$ = 1.08

T_g = 35.2 °C

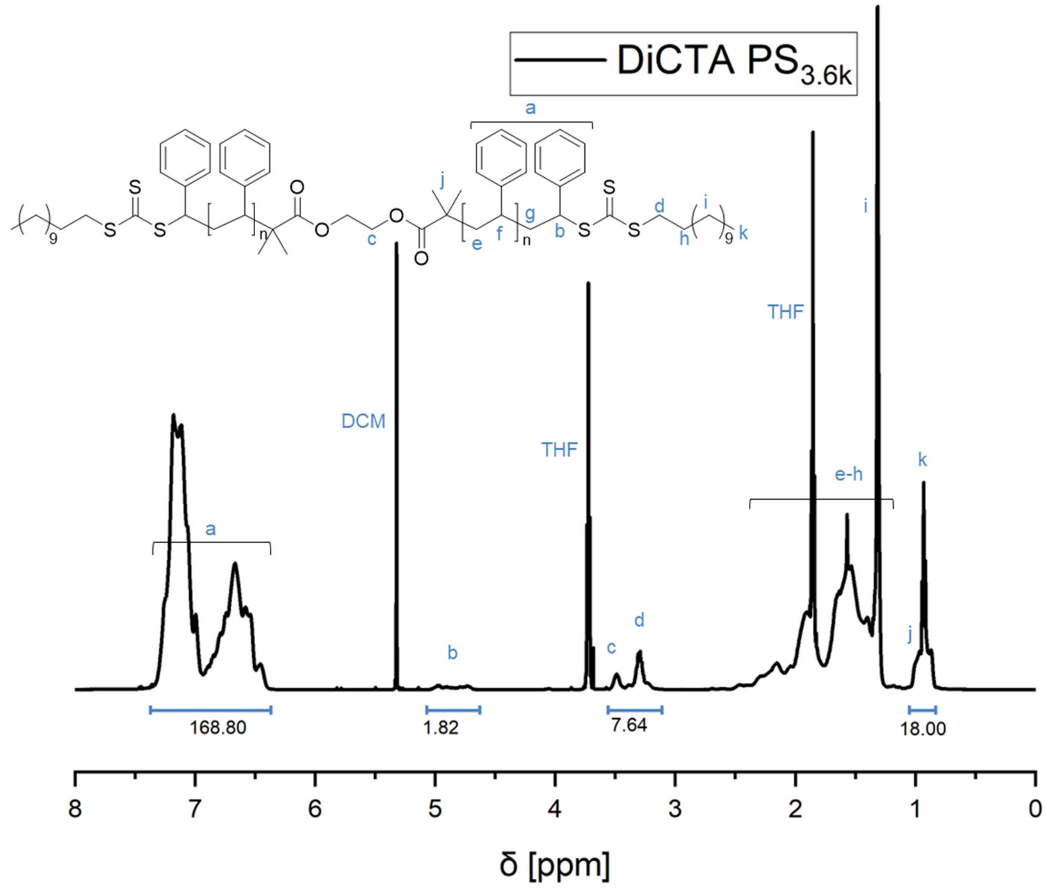

Figure S12. ^{1}H-NMR spectrum of DiCTA-PS$_{3.6k}$

^{1}H-NMR: (500 MHz, CD_2Cl_2, δ in ppm): 7.22-6.47 (m, 169 H, Ar-H), 4.97-4.68 (m, 2H), 3.43 (b, 2H), 3.27 (m, 4H), 2.35-1.28 (m), 0.97-0.84 (m, 18 H).

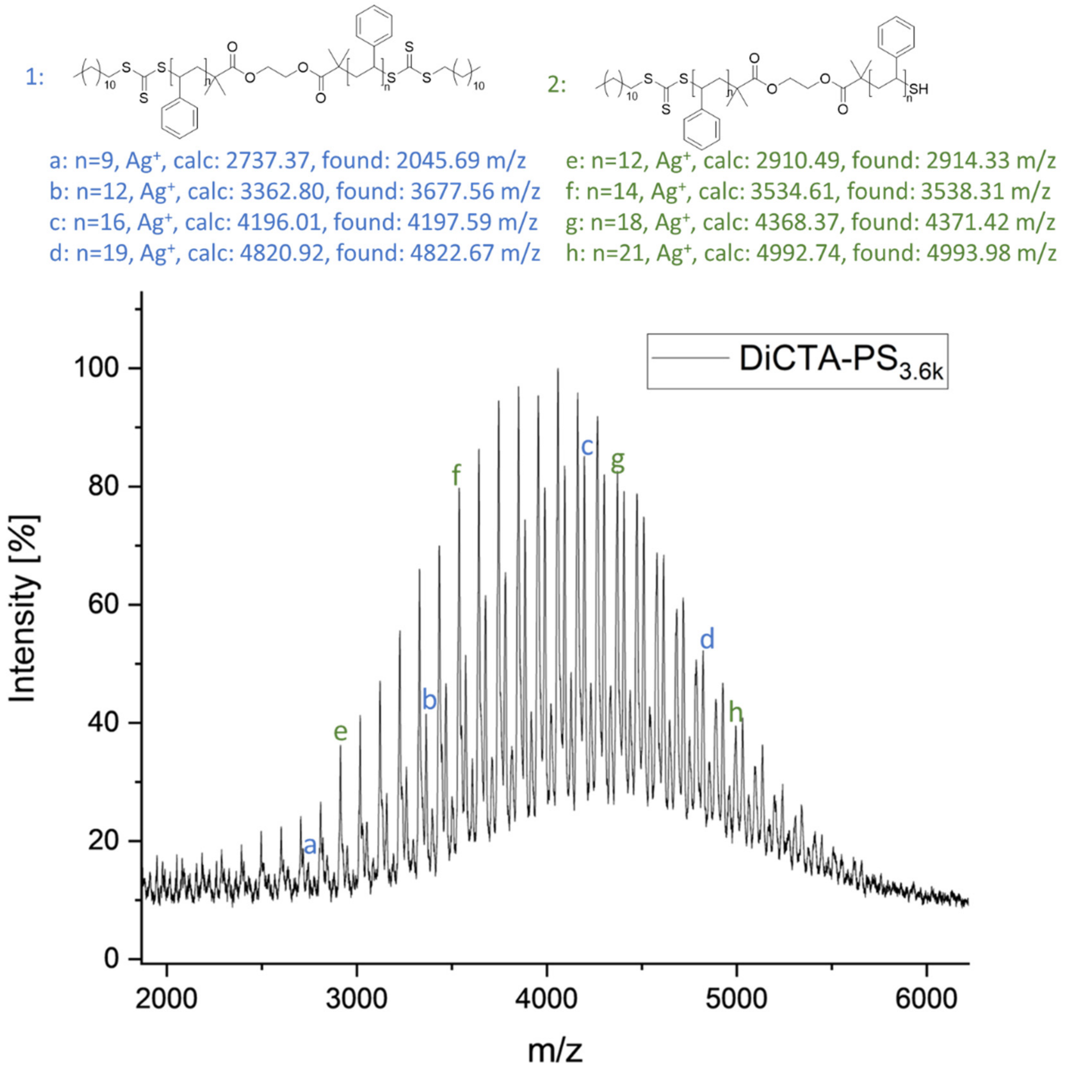

Figure S13. MALDI-ToF-MS spectrum of DiCTA-PS$_{3.6k}$ measured in linear positive mode with AgTFA. During MALDI-ToF-MS measurements, the laser power leads to fragmentations of the colored polymer. Due to the broad signals in the measurement spectra, for some peaks a deviation between calculated m/z and measured peak maximum occurs. The homologous row can be assigned to polystyrene.

MALDI-ToF-MS:

Structure 1: *m/z* 2637.95 (Ag^+, 15%), 2745.69 (16), 2843.04 (18), 2949.4 (19), 3051.36 (23), 3157.48 (28), 3261.05 (33), 3364.63 (41), 3469.27 (47), 3572.37 (51), 3677.56 (62), 3782.23 (65), 3885.97 (74), 3989.31 (78), 4093.71 (83), 4197.59 (85), 4302.47 (82), 4406.42 (79), 4510 (75), 4615.12 (68), 4717.5 (62), 4822.67 (52), 4926.64 (47), 5030.71 (41), 5134.84 (36), 5241.78 (30), 5340.00 (26),

Structure 2: *m/z* 2288.86 (Ag^+, 19%), 2392.75 (19), 2496.57 (22), 2601.87 (22), 2704.88 (24), 2809.64 (27), 2914.33 (36), 3017.79 (41), 3122.26 (47), 3226.89 (56), 3329.92 (66), 3434.02 (70), 3538.31 (80), 3642.71 (86), 3746.59 (95), 3850.74 (97), 3955.12 (95), 4059.07 (100), 4162.82 (96), 4267.26 (92), 4267.26 (83), 4474.27 (79), 4579.95 (69), 4685.22 (59), 4786.37 (51), 4889.62 (44), 4993.98 (40), 5098.41 (33), 5205.67 (27), 5310.19 (24), 5412.22 (22),

DiCTA-PS$_{6.3k}$ polymer

25.0 g styrene (240 mmol) was destabilized over basic aluminum oxide and dissolved in 50% dioxane. 1.81 g of bifunctional RAFT CTA (DiCTA) (2.37 mmol) and 39.0 mg of AIBN (0.237 mmol) were added. The mixture was degassed with argon for 1 h in an ice bath at 5 °C. The reaction was started by stirring the mixture in an oil bath at 70 °C. The polymerization was stopped after 48 h by flooding the sample with oxygen. The polymer mixture was evaporated under reduced pressure, redissolved with tetrahydrofuran and precipitated in cold methanol.

The ratio of monomer : DiCTA : AIBN was 1000 : 1 : 0.1

M_n = 6510 g/mol (SEC)

^{1}H-NMR ratio of CTA Z-group : CTA R-group : styrene = 1 : 1.1 : 57.8 → $M_{n,NMR}$ = 6745 g/mol

M_w = 7100 g/mol

M_p = 7270 g/mol

$Đ$ = 1.09

T_g = 51.3 °C

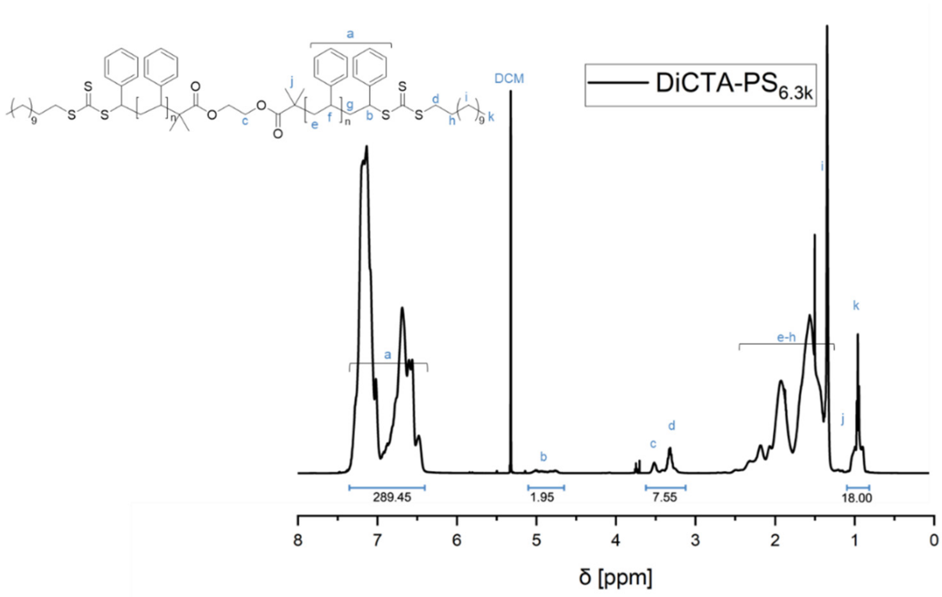

Figure S14. ^{1}H-NMR spectrum of DiCTA-PS$_{6.3k}$.

^{1}H-NMR: (500 MHz, CD_2Cl_2, δ in ppm): 7.23-6.42 (m, 289H, Ar-H), 4.93-4.64 (m, 2H), 3.45 (b, 2H), 3.26 (m, 4H), 2.28-1.28 (m), 0.94-0.843(m, 18H).

FTIR [cm^{-1}]: 3744, 3732, 3705, 3684, 3659, 3624, 3607, 3593, 3082, 3063, 3028, 2974, 2924, 2853, 2384, 2363, 2351, 2339, 2328, 2307, 2295, 1730, 1603, 1493, 1452, 1391, 1300, 1279, 1257, 1182, 1153, 1126, 1067, 1032, 908, 866, 812, 758, 696, 663, 619, 461.

Raman [cm^{-1}]: 3055, 2999, 2974, 2908, 2852, 1646, 1632, 1602, 1585, 1447, 1302, 1199, 1183, 1155, 1065, 1031, 1002, 908, 793, 759, 618, 516.

Further characterization

SEC

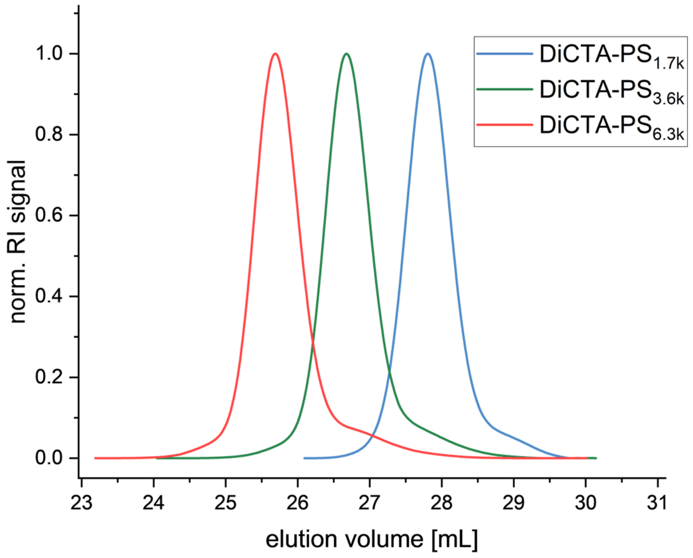

Figure S15. SEC spectra of all three DiCTA-PS$_x$.

Overview apparent molecular weight measured via SEC and NMR

Table S1: Comparison of molecular weight measured via SEC and NMR. The calculated deviation lies well within the SEC measurement error.

	Polymer	$M_{n,SEC}$ [g/mol]	$M_{n,NMR}$ [g/mol]
DiCTA-PS	$PS_{1.7k}$	2040	2200
	$PS_{3.6k}$	3740	4270
	$PS_{6.3k}$	6510	6740

Synthesis of Dithiol-PS$_x$ polymers[5]

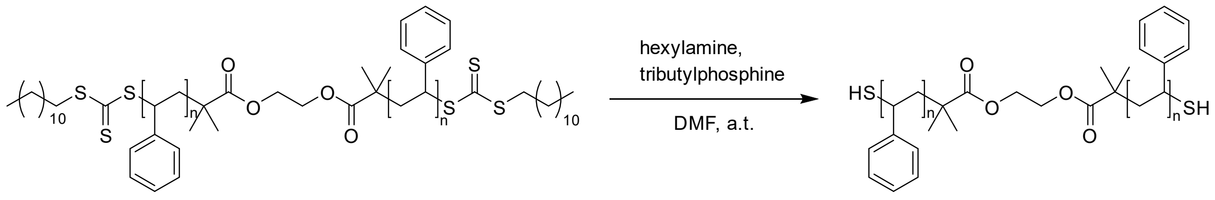

1.0 eq. of RAFT polymer was dissolved in *N*,*N*-dimethylformamide. 60 eq. of hexylamine and 2.0 eq. of tributyl phosphine were added. The mixture was stirred for 30 min at ambient temperature. The color changed from yellow to colorless. Afterwards, it was filtered over aluminum oxide (acidic) and precipitated in pure methanol and dried under reduced pressure. A white powder was obtained with a yield of 82-89 %.

Dithiol-PS$_{1.7k}$ polymer

M_n = 1740 g/mol

M_w = 1880 g/mol

M_p = 1770 g/mol

$Đ$ = 1.08

T_g = 40.6 °C

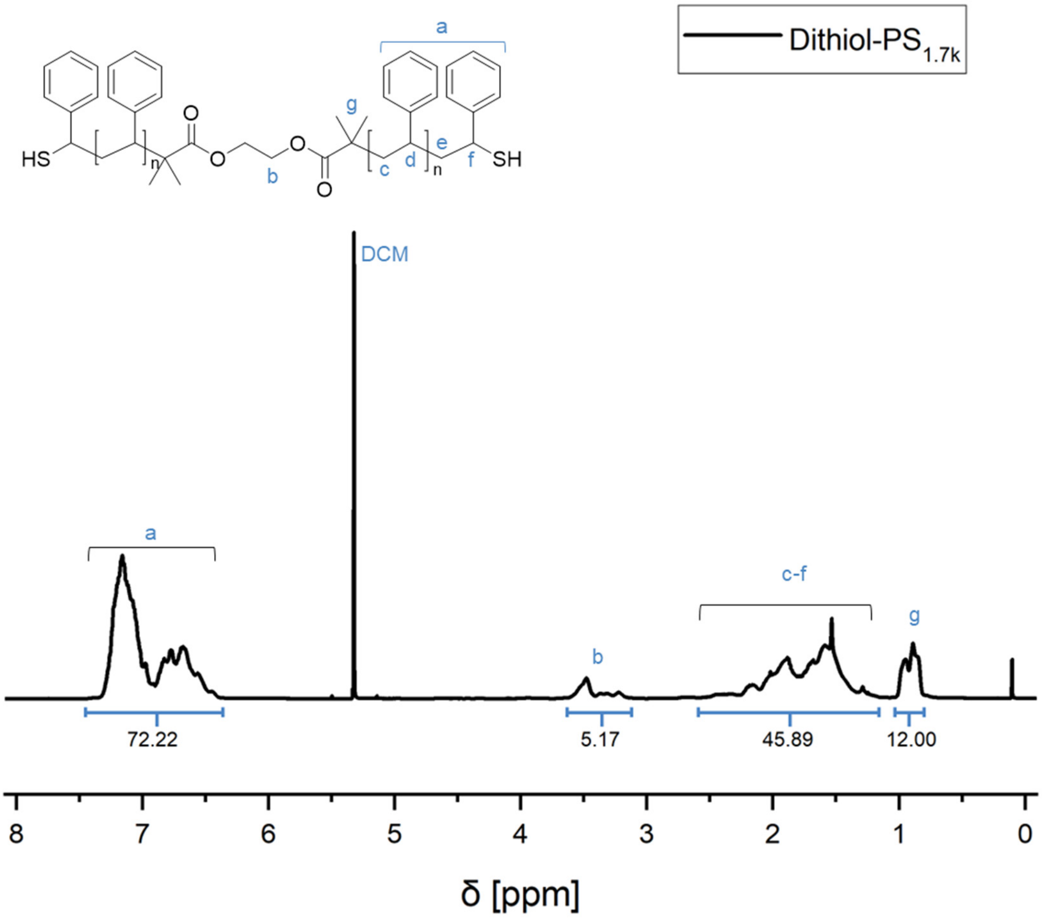

Figure S16. ^{1}H-NMR spectrum of Dithiol-PS$_{1.7k}$. CTA chain signals at 0.92 ppm (-CH_3), 1.3 ppm (-CH_2-) and 3.1-3.3 ppm (-S(C=S)-S-CH_2-) disappear after aminolysis.

^{1}H-NMR: (500 MHz, CD_2Cl_2, δ in ppm): 7.26-6.45 (m, 70H, Ar-H), 3.59-3.24 (m, 4H, O-C-H), 2.73 – 1.18 (m, 42H, backbone), 0.95-0.85 (m, 12H, -CH3).

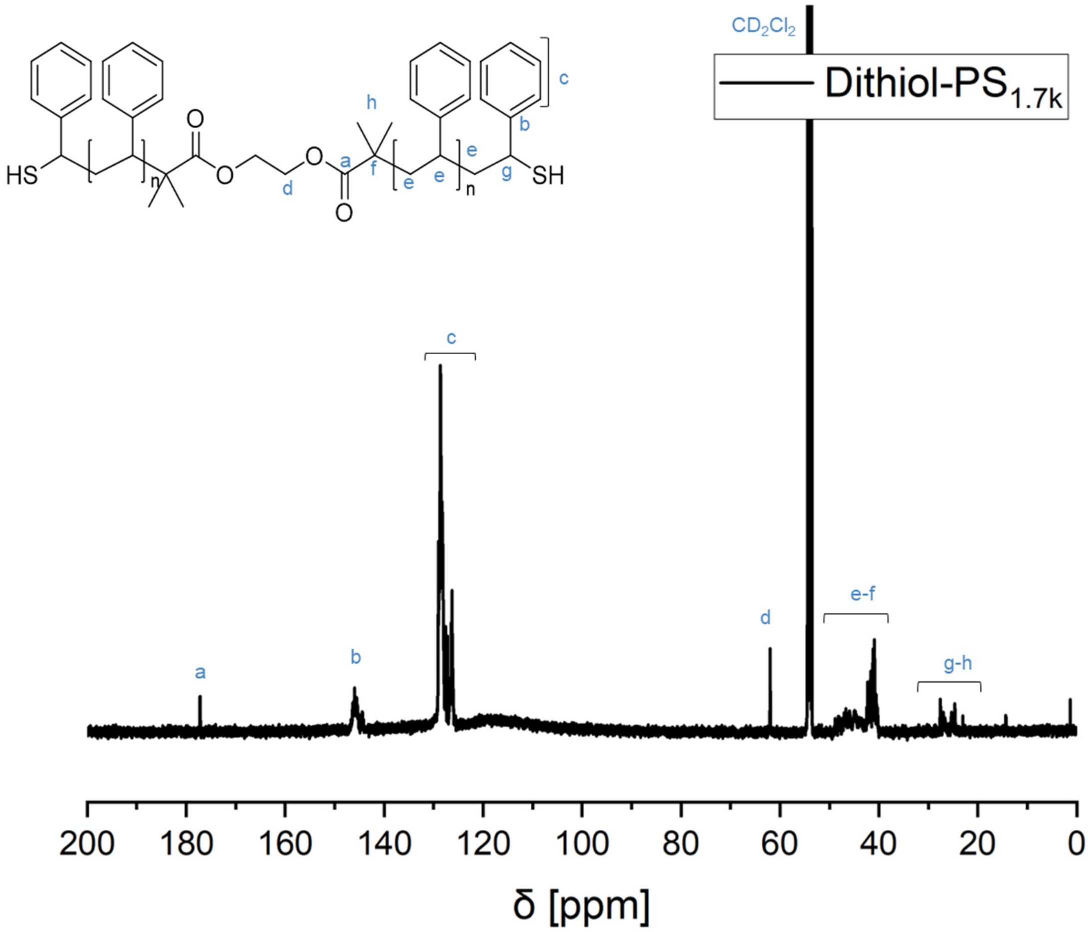

Figure S17. ^{13}C-NMR spectrum of Dithiol-PS$_{1.7k}$. a disappearance of the CTA alkyl chain signals at 23 - 37 ppm and the trithiocarbonate carbon signal at 222 pm indicate a quantitative cleavage.

^{13}C-NMR: (500 MHz, CD_2Cl_2, δ in ppm): 177.2, 146.0, 129.0-126.3, 62.0, 48.8-40.3, 27.0-23.0.

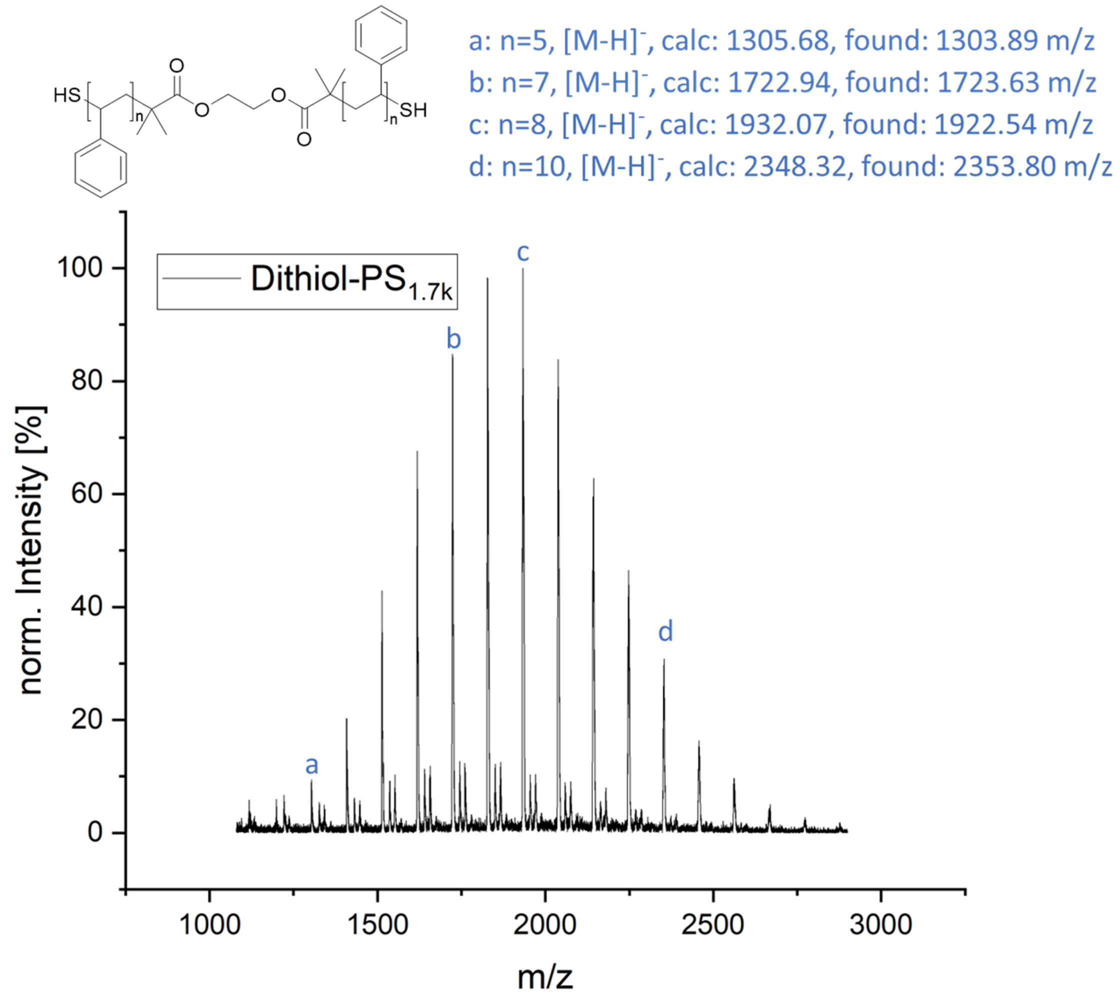

Figure S18. MALDI-ToF-MS spectrum of Dithiol-PS$_{1.7k}$ measured in linear negative mode. No traces of DiCTA-PS$_{1.7k}$ were found. However, small traces of an incomplete aminolysis product with a single sided dithiocarbamate as intermediate were found. In NMR spectroscopy the intermediate was not detected and also appeared to have no impact on any subsequent reactions. It is thus likely that the peak ratios in the MALDI-ToF-MS spectrum are not directly indicative of the molar ratios of the species.

MALDI-ToF-MS: *m/z* 1303.89 (neg, 9.4%), 1408.6 (20.3),1514.2 (42.9), 1618.93 (67.6), 1723.63 (84.8), 1828.14 (98.3), 1933.54 (100.0), 2037.97 (83.8), 2144.52 (62.8), 2248.17 (46.5), 2353.8 (30.8), 2457.44 (16.4), 2562.3 (9.6), 2669.77 (5.1).

Dithiol-PS$_{3.6k}$ polymer

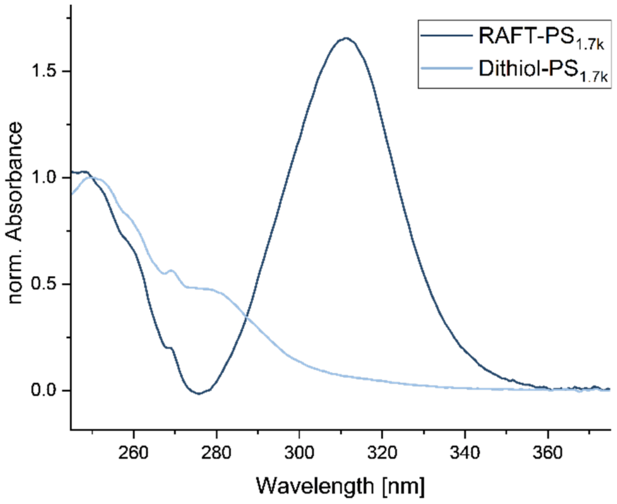

Figure S19. UV/Vis spectra of DiCTA-PS$_{1.7k}$ and Dithiol-PS$_{1.7k}$. After aminolysis, the trithiocarbonate peak from 340 - 480 nm disappears, along with a discoloration of the yellow polymer.

M_n = 3640 g/mol; M_w = 4150 g/mol; M_p = 3680 g/mol; $Đ$ = 1.14

T_g = 75.8 °C

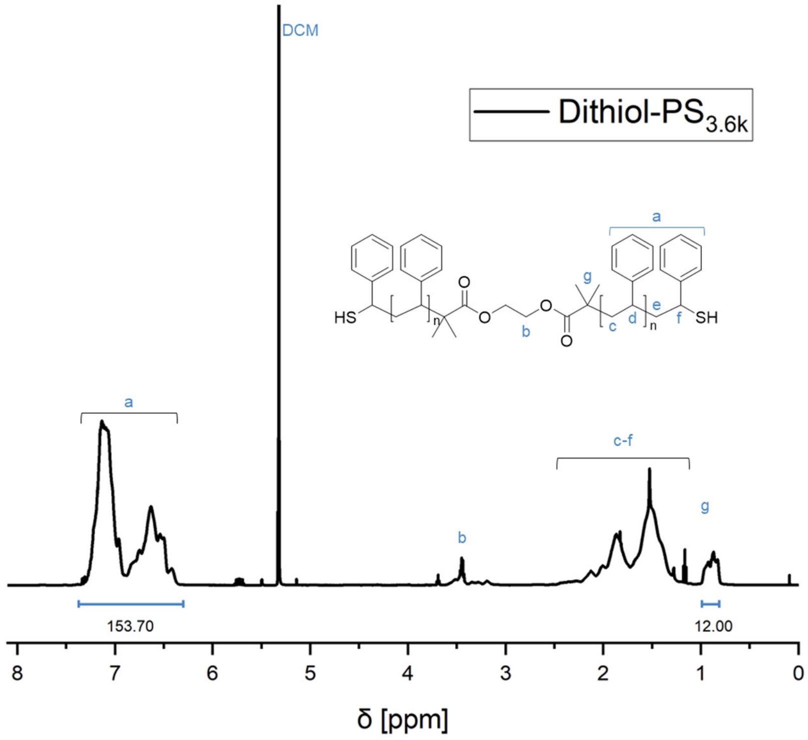

Figure S20. ^{1}H-NMR spectrum of Dithiol-PS$_{3.6k}$. CTA chain signals at 0.92 ppm (-CH_3), 1.3 ppm (-CH_2-) and 3.1-3.3 ppm (-S(C=S)-S-CH_2-) disappear after aminolysis.

^{1}H-NMR: (500 MHz, CD_2Cl_2, δ in ppm): 7.28-6.33 (m, 169H, Ar-H), 3.59-3.14 (m, 4H, O-C-H), 2.46-1.31 (m, backbone), 0.95-0.83 (m, 12H, -CH3).

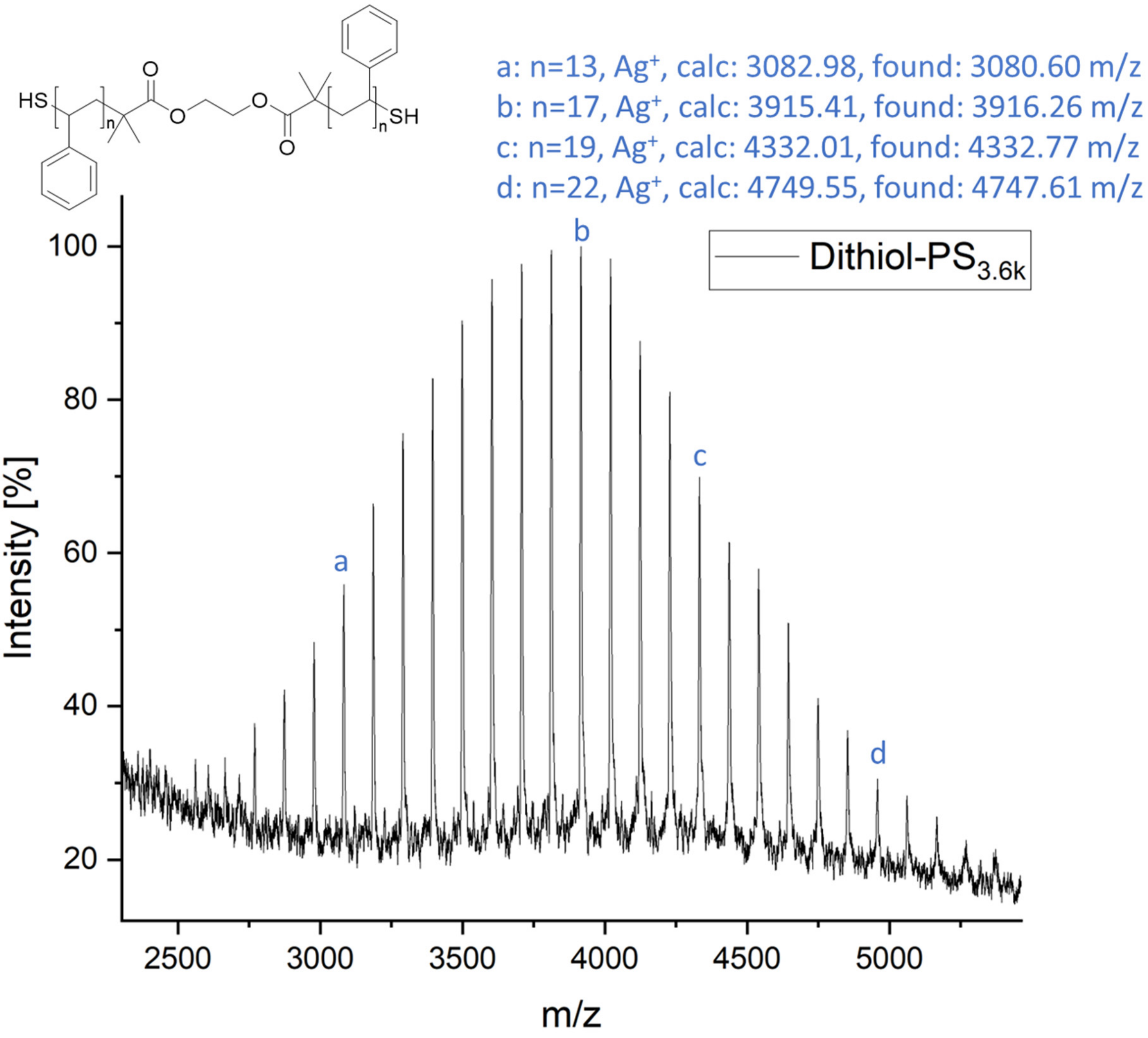

Figure S21. MALDI-ToF-MS spectrum of Dithiol-PS$_{3.6k}$ measured with AgTFA in linear positive mode.

MALDI-ToF-MS: *m/z* 2769.61 (Ag^+, 37.7%), 2874.08 (42.2), 2978.39 (48.4), 3082.98 (55.9), 3186.4 (66.4), 3290.99 (75.6), 3395.6 (82.8), 3499.02 (90.3), 3603.71 (95.7), 3707.61 (97.7), 3812.11 (99.5), 3916.26 (100.0), 4020.61 (98.4), 4124.18 (87.7), 4228.76 (81.1), 4332.77 (69.9), 4437.4 (61.4), 4540.7 (57.9), 4644.86 (50.9), 4749.55 (41.0), 4853.07 (36.8), 4957.72 (30.5), 5062.11 (28.3), 5166.57 (25.6).

Dithiol-PS$_{6.3k}$ polymer

M_n = 6270 g/mol

M_w = 7210 g/mol

M_p = 6560 g/mol

$Đ$ = 1.15

T_g = 85.7 °C

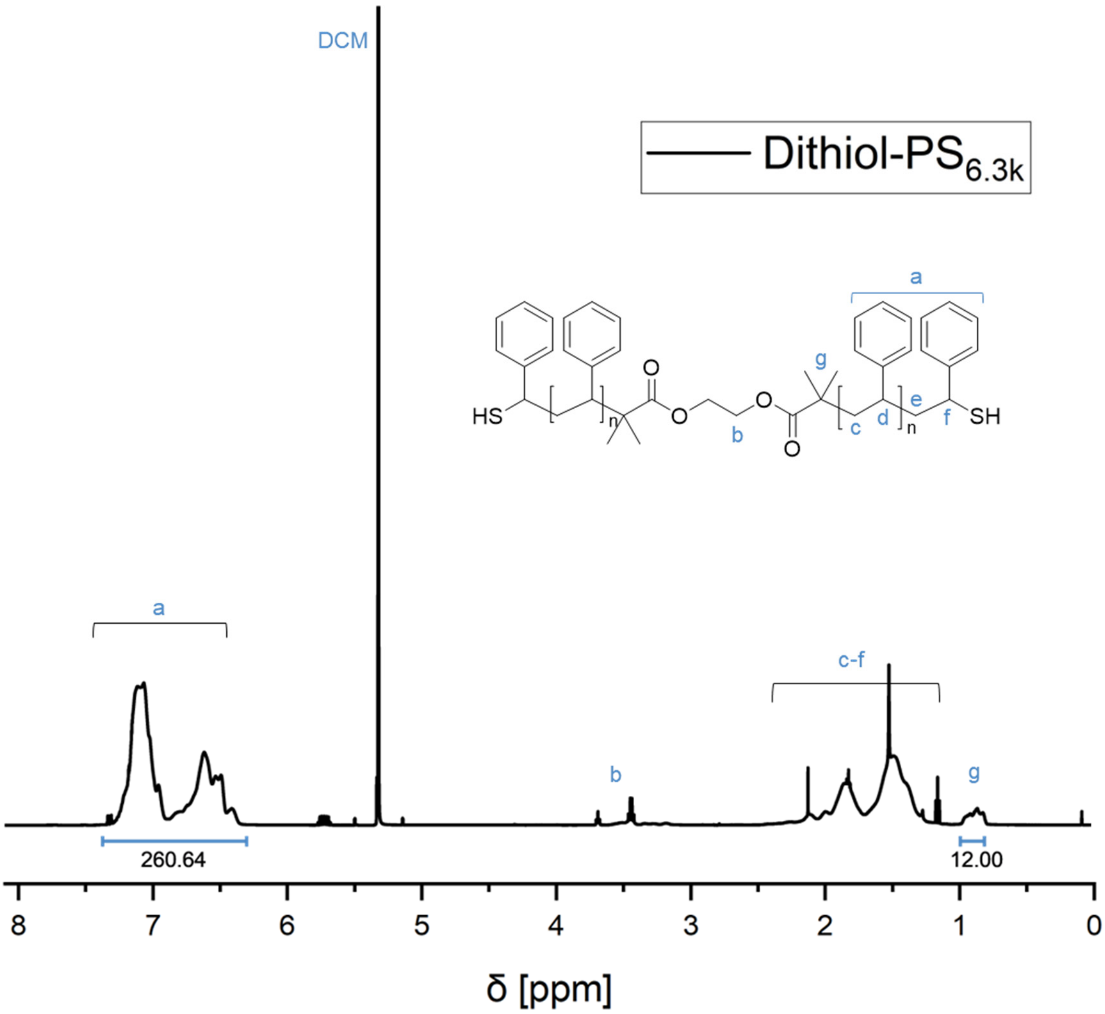

Figure S22. ^{1}H-NMR spectrum of Dithiol-$PS_{6.3k}$. CTA chain signals at 0.92 ppm (-CH_3), 1.3 ppm (-CH_2-) and 3.1-3.3 ppm (-S(C=S)-S-CH_2-) disappear after aminolysis.

^{1}H-NMR: (500 MHz, CD2Cl2, δ in ppm): 7.336-6.43 (m, 289H, Ar-H), 3.61-3.16 (m, 4H, O-C-H), 2.35-1.23 (m, backbone), 0.97-0.84 (m, 12H, -CH3).

MALDI-ToF-MS: Dithiol-$PS_{6.3k}$ could not be detected in MALDI-ToF-MS measurements due to its high molecular weight.

Further Dithiol-PS$_x$ characterization

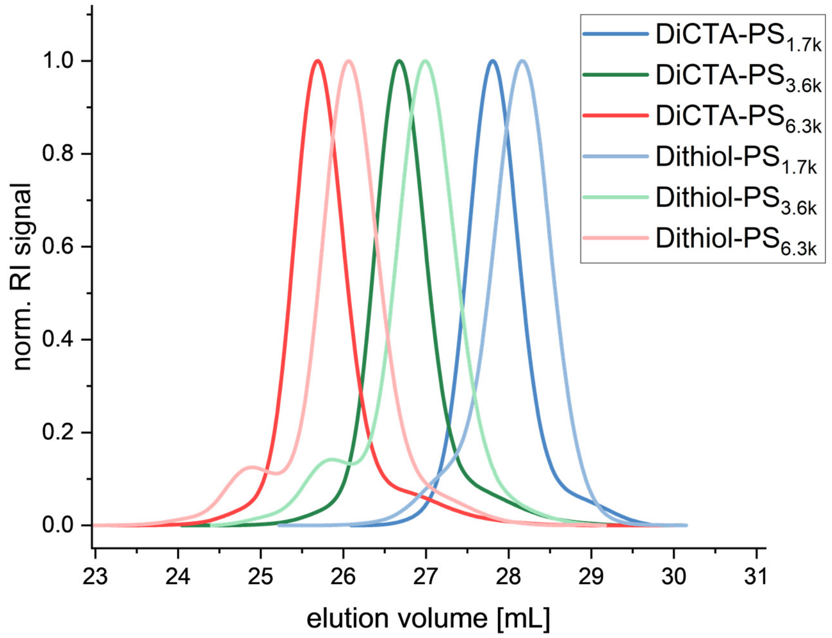

Figure S23. Shift of peak molecular weight measured via THF-SEC. M_p: calc. 489 g/mol, found: Dithiol-PS$_{1.7k}$: 450 g/mol, Dithiol-PS$_{3.6k}$: 400 g/mol, Dithiol-PS$_{6.3k}$: 700 g/mol.

FTIR [cm^{-1}]: 3084, 3063, 3028, 3003, 2978, 2964, 2924, 2876, 2854, 1728, 1603, 1493, 1474, 1452, 1391, 1315, 1279, 1182, 1153, 1124, 1068, 1030, 756, 696.

Raman [cm^{-1}]: 3052, 2999, 2974, 2908, 2854, 2567, 1602, 1582, 1450, 1325, 1199, 1183, 1155, 1068, 1031, 1002, 791, 761, 618, 219.

RAFT group cleavage is indicated by disappearance of S-C-S symmetrical stretching and C=S stretching bands at 516 cm^{-1} and 1065 cm^{-1}, aliphatic C-S stretching vibration stays at 793 cm^{-1} and new S-H stretching vibration signal at 2567 cm^{-1} appears.[6, 7]

Synthesis of TCC-PS$_{1.7k}$/TCC-PS$_{3.6k}$ model systems

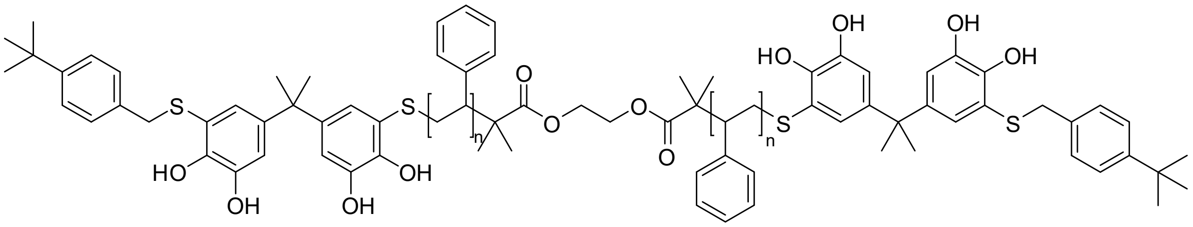

0.011 mmol of TCC-PS (TCC-PS$_{1.7k}$: 100 mg, 1.0 eq; TCC-PS$_{3.6k}$: 153 mg, 1.0 eq) was dissolved in 2 mL DMF and added to a solution of 100 mg BQA (0.391 mmol, 35 eq) in DMF (c = 10 mg/mL) and stirred for 1 h. Subsequently, 198 mg 4-*tert*-Butylbenzyl mercaptan (1.10

mmol, 100 eq) was added until the solution had a light brown color. The mixture was stirred overnight and then precipitated in cold methanol.

This system consisted of a PS dithiol (Dithiol-$PS_{1.7k}$ and Dithiol-$PS_{3.6k}$) with their thiol end groups saturated with an excess of BQA. 4-*tert*-Butylbenzyl mercaptan was added to this system as an NMR probe to react with quinone end groups, as the BQA signals were overlapped by the signals from the polystyrene protons. The distinct *tert*-butyl proton signals at 1.32 ppm as well as the additional aromatic signals at 7.26 and 7.35 ppm of the NMR probe make it possible to calculate integral ratios between the *tert*-butyl group of the probe and the solitary methyl groups of the RAFT CTA core of the PS dithiol block at 0.80 – 1.03 ppm.

They showed an equal ratio of NMR probe to end group of the TCC model polymer and displays a high end group reactivity of the TCC-PS quinone groups as well as a high end group fidelity of the PS-Dithiol polymer.

Characterization TCC-PS$_{1.7k}$ model system

NMR

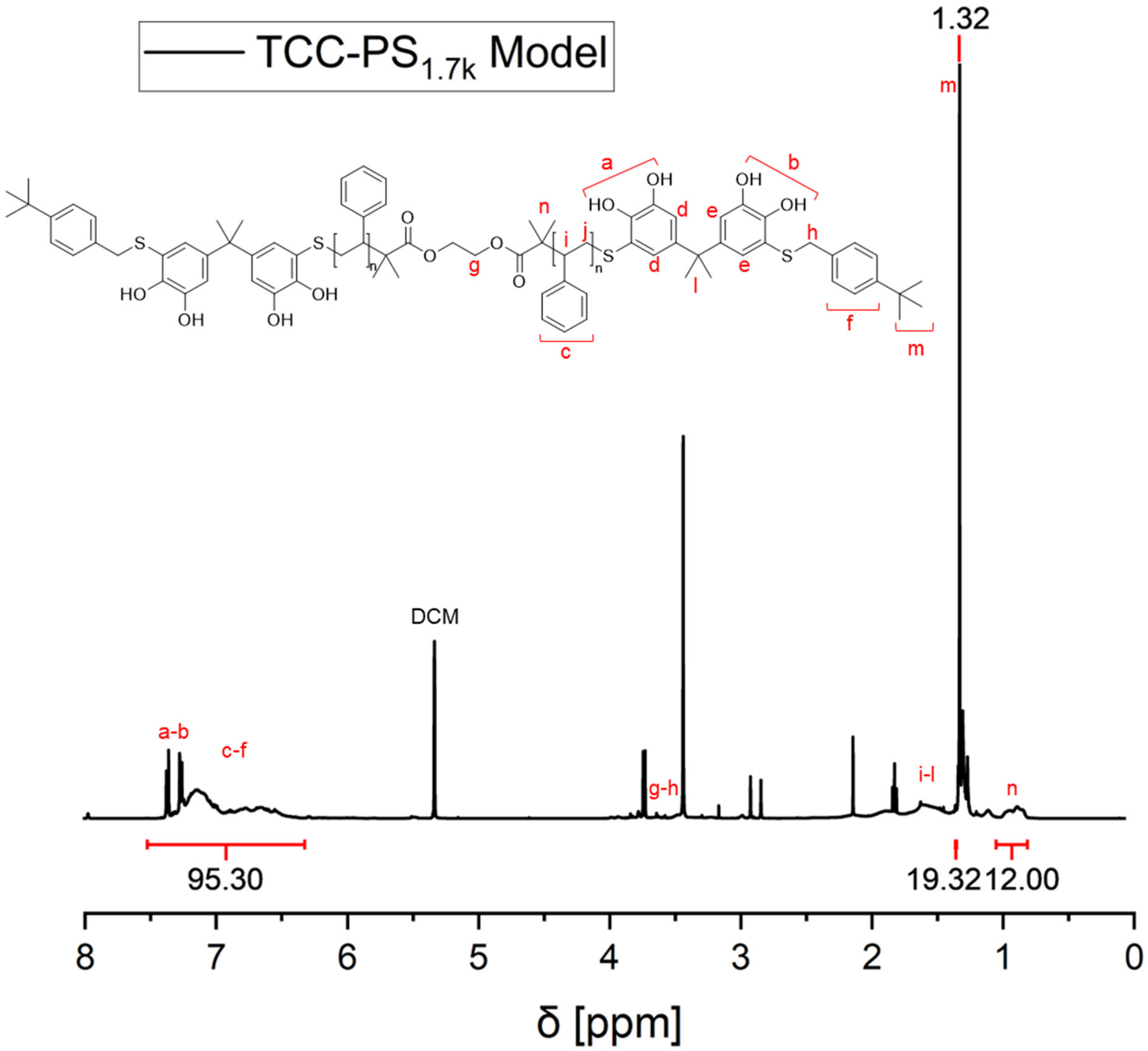

Figure S24. ^{1}H-NMR spectrum of the TCC-PS$_{1.7k}$ model.

NMR spectroscopy integrals show a proton ratio of methyl groups of the central CTA core : methyl groups of the *tert*-butyl endcaps : aromatic protons of 12 : 19 : 95. Subtraction of 24 aromatic protons coming from 4-*tert*-Butylbenzyl and BQA-incorporation results in 71 aromatic protons assigned to 14.2 styrene units (compared to originally 13.9 styrene units calculated in DiCTA-PS$_{1.7k}$).

SEC

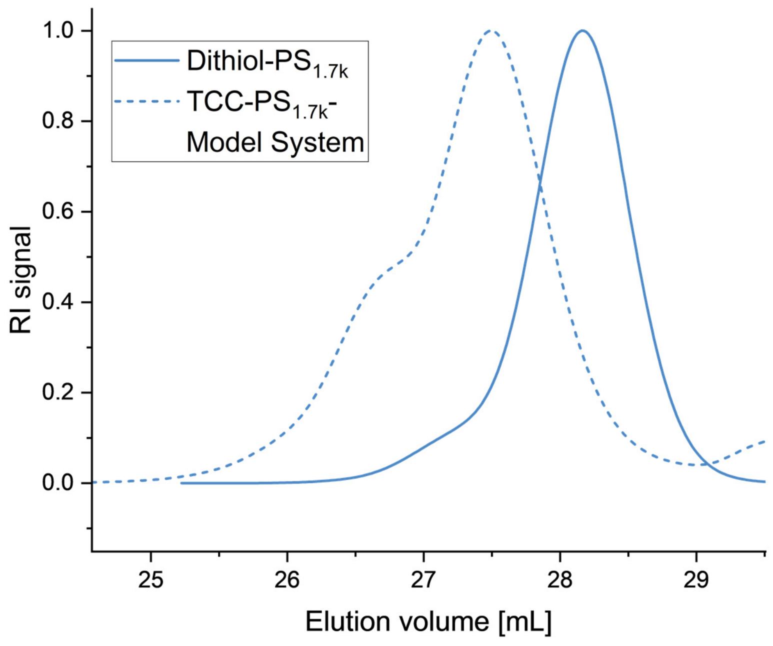

Figure S 25. SEC measurements of the model polymer showed a shift of M_p accordingly to the increased molar mass (calc.: 1.197 g/mol, found: 1080 g/mol) and a dispersity increase to only Đ = 1.13, confirming only a slight number of dimeric species.

MALDI-ToF-MS

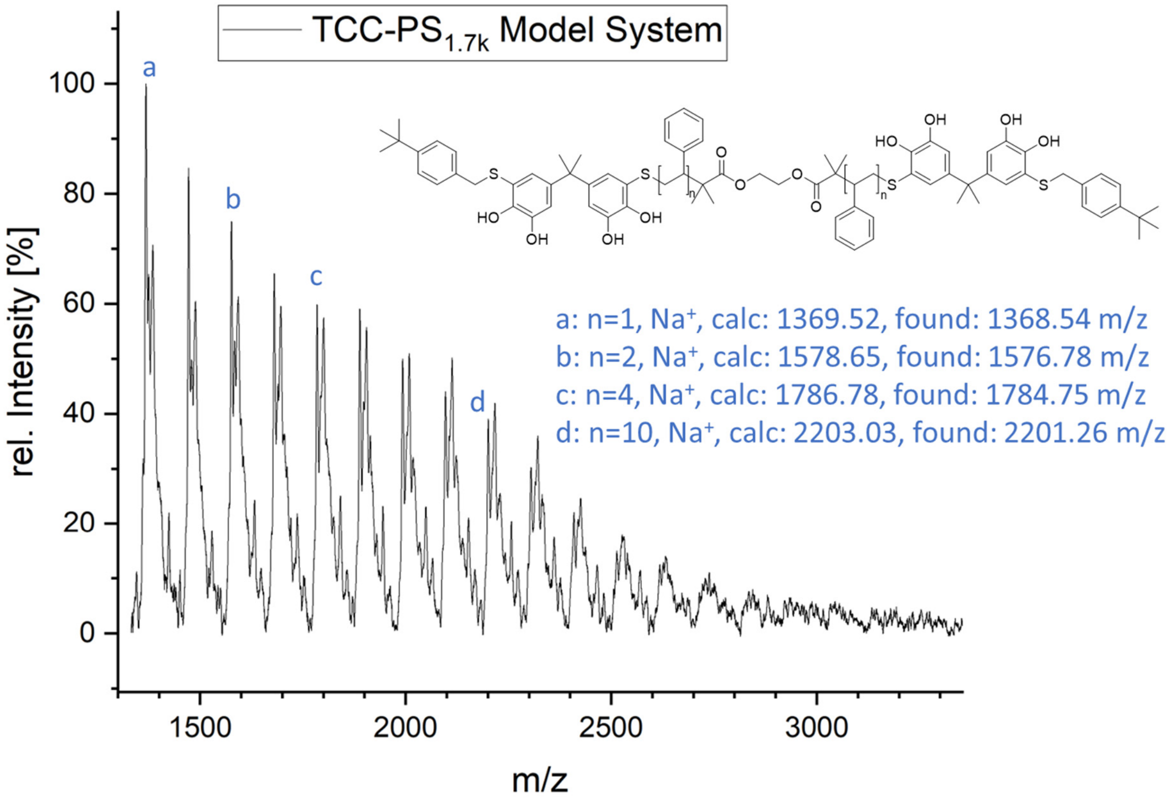

Figure S26. MALDI-ToF-MS spectrum of the TCC-PS$_{1.7k}$ model system. Measured in linear positive mode with NaTFA. The dominating signals can be assigned as Na^+ adduct, a second homologous row can be assigned to K^+ adducts, which may originate from ions present in the environment. Additionally, the yellow colored polymer could negatively influence the desorption process, which might lead to broad signals.

m/z 1368.54 (Na^+, 100%), 1472.45 (85), 1576.78 (75), 1680.8 (66), 1784.75 (60), 1888.74 (59), 1992.93 (50), 2097.32 (44), 2201.26 (39), 2304.98 (30), 2409.7 (22), 2513.9 (15), 2618.61 (13).

m/z 1385.33 (K^+, 70), 1489.13 (60), 1593.28 (61), 1696.67 (60), 1800.9 (57), 1905.15 (56), 2009.36 (51), 2113.29 (50), 2217.4 (42), 2321.96 (36), 2425.43 (25), 2531.17 (18).

Characterization TCC-$PS_{3.6k}$ model system

NMR

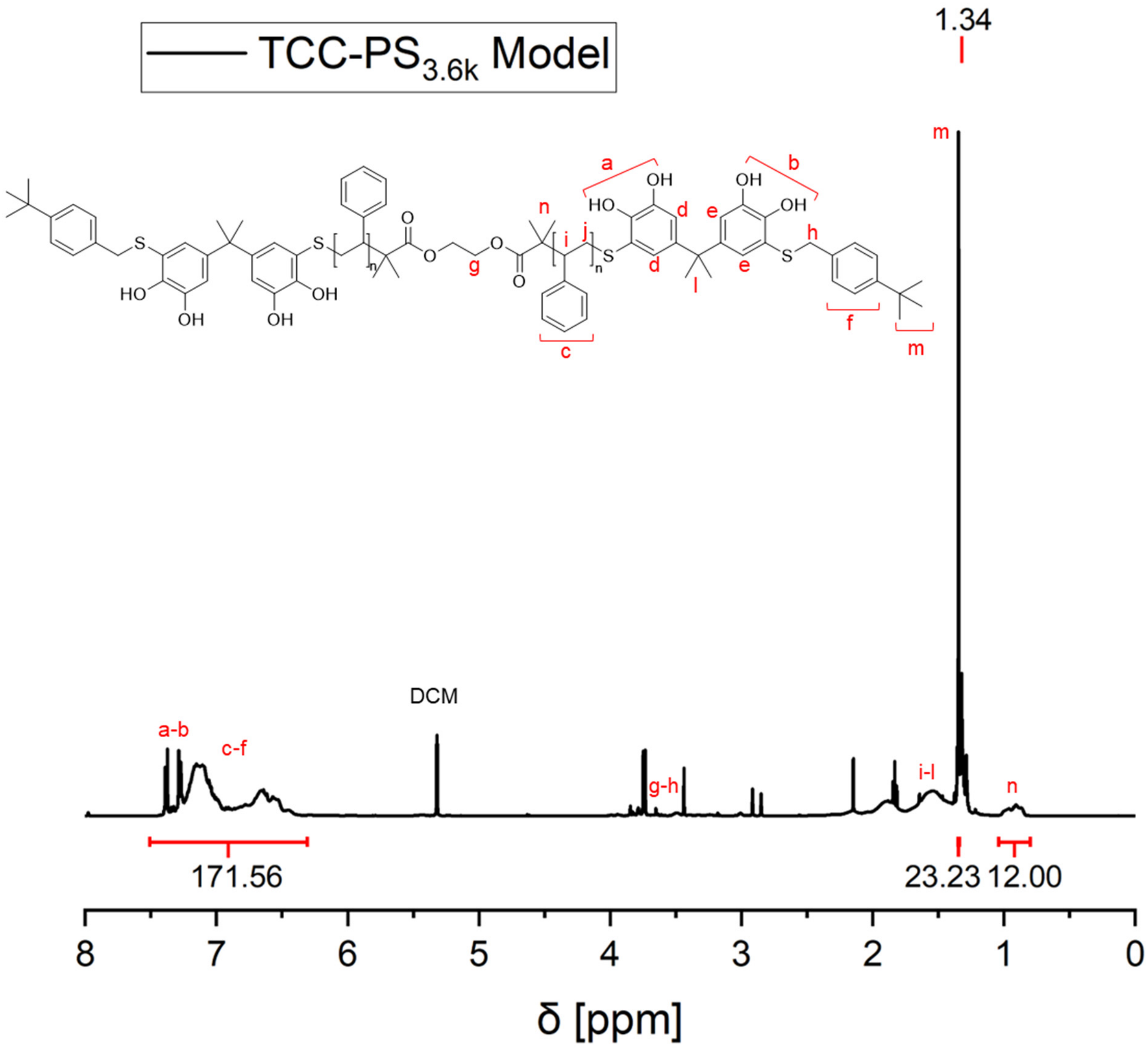

Figure S27. ^{1}H-NMR spectrum of the TCC-$PS_{3.6k}$ model system.

NMR spectroscopy integrals show a proton ratio of methyl groups of the central CTA core : methyl groups of the *tert*-butyl endcaps : aromatic protons of 12 : 23 : 172. Subtraction of 24 aromatic protons coming from 4-*tert*-Butylbenzyl and BQA-incorporation results in 148 aromatic protons assigned to 29.6 styrene units (compared to originally 31.9 styrene units calculated in DiCTA-$PS_{1.7k}$).

SEC

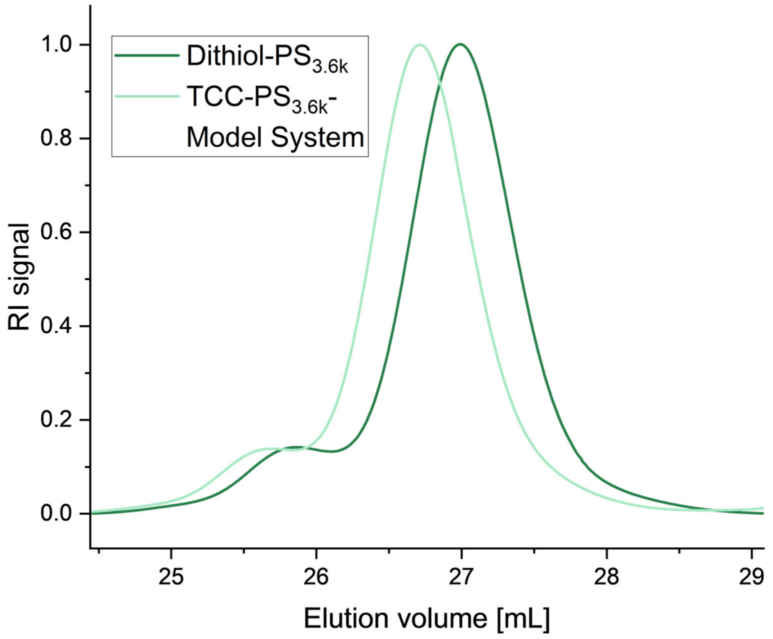

Figure S 28. SEC measurements of the model polymer showed a shift of M_p accordingly to the increased molar mass (calc.: 1.197 g/mol, found: 912 g/mol) while still obtaining a low dispersity of Đ < 1.2, confirming only a slight number of dimeric species.

MALDI-ToF-MS

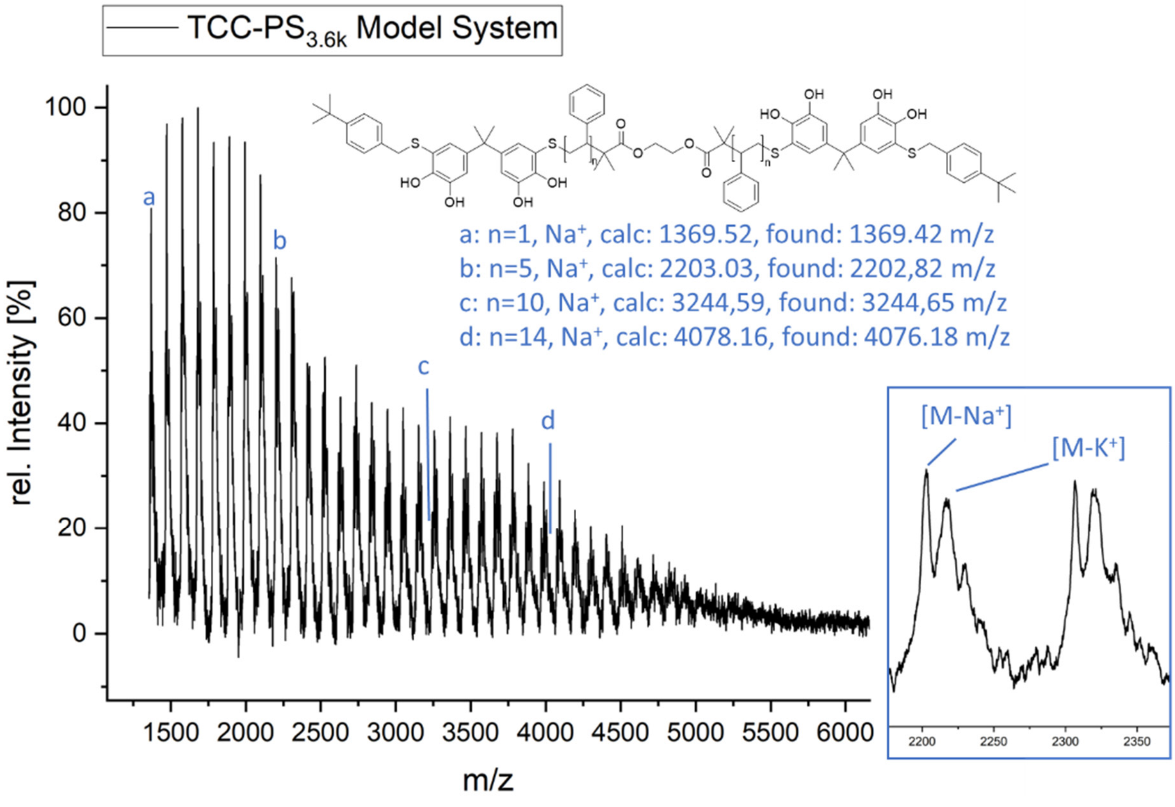

Figure S29. MALDI-ToF-MS spectrum of the TCC-$PS_{3.6k}$ model system. Measured in linear positive mode with NaTFA. The dominating signals can be assigned as Na^+ adduct, a second homologous row can be assigned to K^+

adducts, which may originate from ions present in the environment. Additionally, the yellow colored polymer could negatively influence the desorption process, which might lead to broad signals.

m/z 1369.42 (Na^+, 81%), 1473.54 (97), 1577.72 (98), 1681.78 (100), 1786.57 (93), 1890.61 (94), 1994.63 (93), 2098.19 (87), 2202.82 (71), 2306.58 (68), 2410.88 (51), 2516.06 (46), 2620.57 (34), 2722.74 (39), 2827.38 (32), 2932.21 (28), 3035.53 (26), 3139.59 (25), 3244.59 (23), 3348.3 (22), 3452.52 (30), 3556.08 (21), 3660.02 (27), 3762.82 (21), 3869.15 (21), 3971.84 (20), 4076.18 (20), 4179.42 (17), 4285.53 (14), 4387.9 (13), 4493.73 (12), 4596.29

m/z 1384.62 (K^+, 44%), 1488.76 (54), 1593.09 (57), 1697.65 (63), 1802.32 (62), 1905.77 (60), 2010.64 (65), 2113.51 (68), 2216.73 (62), 2321.04 (65), 2423.78 (51), 2528.28 (53), 2632.07 (45), 2737.21 (51), 2840.6 (44), 2945.15 (43), 3050.82 (43), 3153.26 (37), 3257.4 (39), 3362.42 (41), 3466.86 (39), 3571.49 (38), 3675.65 (38), 3779.56 (39), 3885.22 (32), 3988.43 (29), 4093.61 (29), 4194.59 (23), 4300.88 (20), 4404.07 (19), 4510.1 (20), 4615.45 (14).

Synthesis of TCC-PS_x polymers[2]

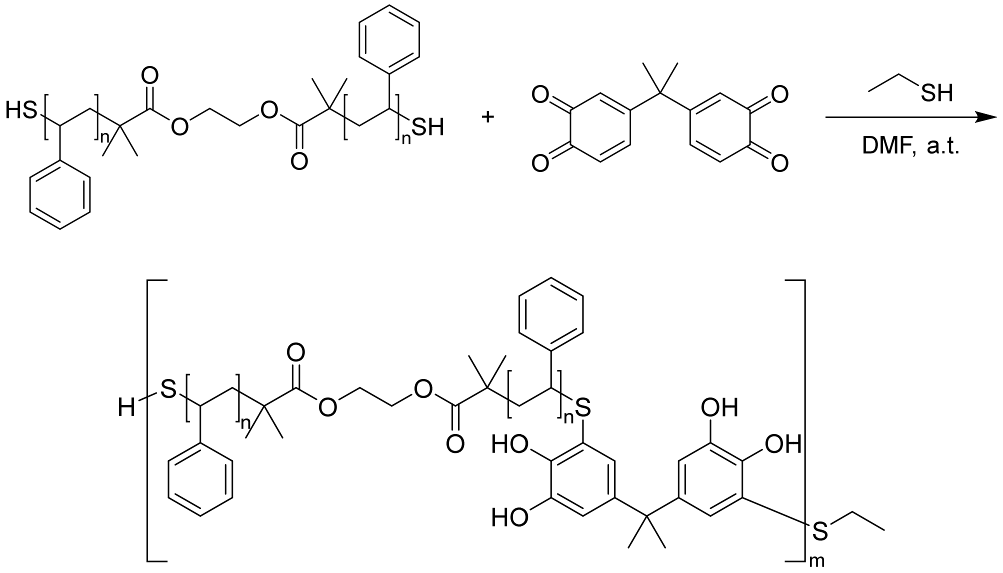

1.0 eq. of dithiol polystyrene was dissolved in *N*,*N*-dimethylformamide (DMF) (c = 0.5 mg/mL). A solution of 0.7 eq. BQA in DMF was added (c(BQA end concentration) = 5 mg/mL) and the mixture was stirred overnight. Next, ethanethiol was added under stirring for 6 h to quench the free quinone end groups. Afterwards, the mixture was precipitated in MeOH/H_2O (v/v = 60/40) to remove small molecular species, unreacted BQA and DMF. The solid was dried under reduced pressure.

For the ^{1}H-NMR measurement, the catechol groups were methylated according to literature: 1.0 eq. TCC polystyrene were solved with 10 eq. K_2CO_3. and 10 eq. MeI in acetone. Then it was stirred under reflux with an NH_3 solution wash bottle connected to the reflux condenser. The mixture was stirred overnight. Afterwards, the product was precipitated twice in cold MeOH and the supernatant was quenched with NH_3 solution. A light brown to white solid was obtained in a yield of 83-91 %.
For the ^{31}P-NMR measurement, the polymer was derivatized with 2-chloro-2-oxo-1,3,2-dioxaphospholane (CDP) using Cr(acac)$_3$ and Ph_3PO as internal standard in pyridine:$CDCl_3$-1.6:1 v/v% at ambient temperature for 15 min. ^{31}P-NMR based calculations are furthermore confirmed by SEC measurements, were the number-average amount of BQA-PS repeating units can be calculated from M_n(TCC-PS) and MW(BQA+Dithiol-PS), resulting in the average amount of BQA and Dithiol-PS units, respectively.

TCC-$PS_{1.7k}$ polymer

M_n = 9,100 g/mol;

M_w = 14,660 g/mol

$Đ$ = 1.61

T_g = 85.5 °C

TGA: max. degradation rate at 436.6 °C, 5 wt% loss at 294.1 °C

M (BQA) = 256 g/mol, M_n ($TCC-PS_{1.7k}$) = 9100 g/mol.

Measured via ^{31}P-NMR: average of 5.1 BQA per chain = 14.3 wt%.

Calculated with ^{1}H-NMR data: 2 TCC groups per 14 styrene groups = 12.5 mol% TCCs per aromatics.

Calculated with SEC data: average of 4.6 BQA per chain = 12.9 wt%.

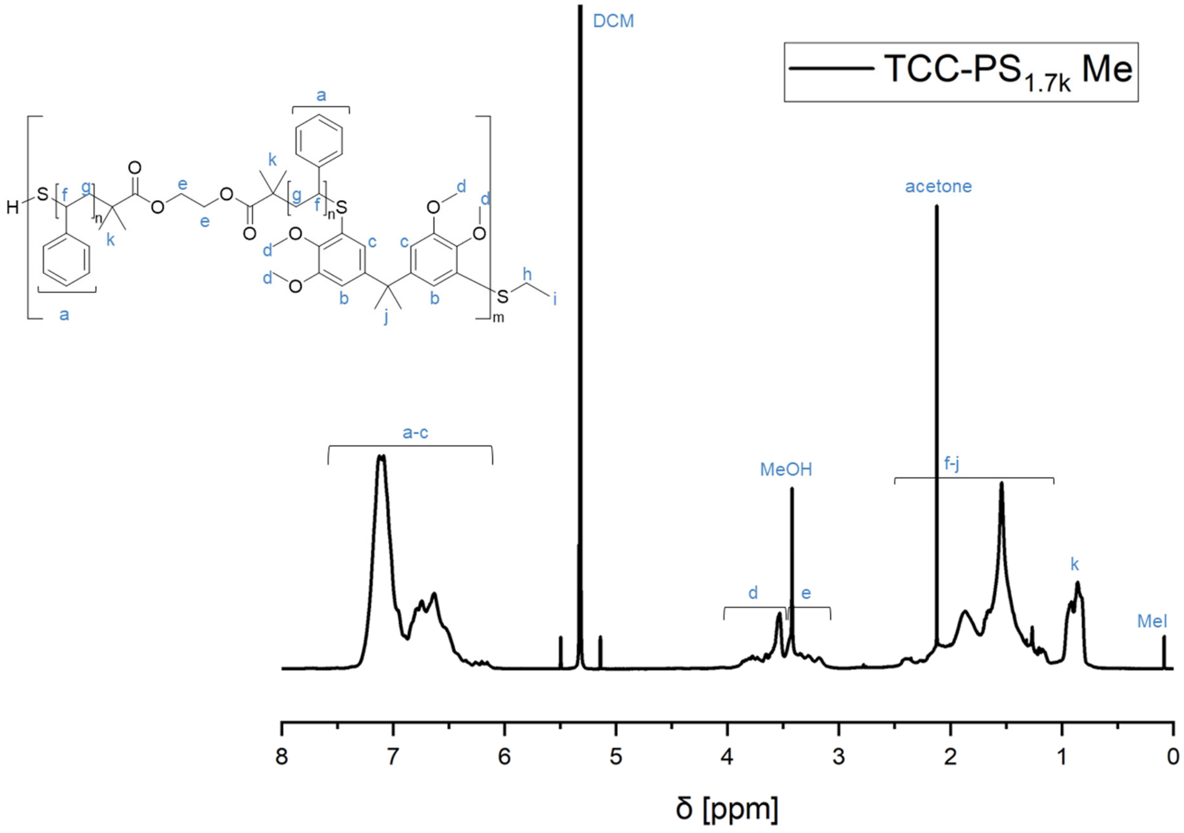

Figure S30. ^{1}H-NMR spectrum of $TCC-PS_{1.7k}$ after methylation of the catechol groups with MeI.

^{1}H-NMR: (500 MHz, CD_2Cl_2, δ in ppm):

7.23-6.13 (m, Ar-H), 3.97-3.49 (m, Ar-O-CH3), 3.38-3.12 (m, 4H, O-C-H), 2.46-1.10 (m, backbone), 0.94-0.82 (m, 12H, $-CH_3$).

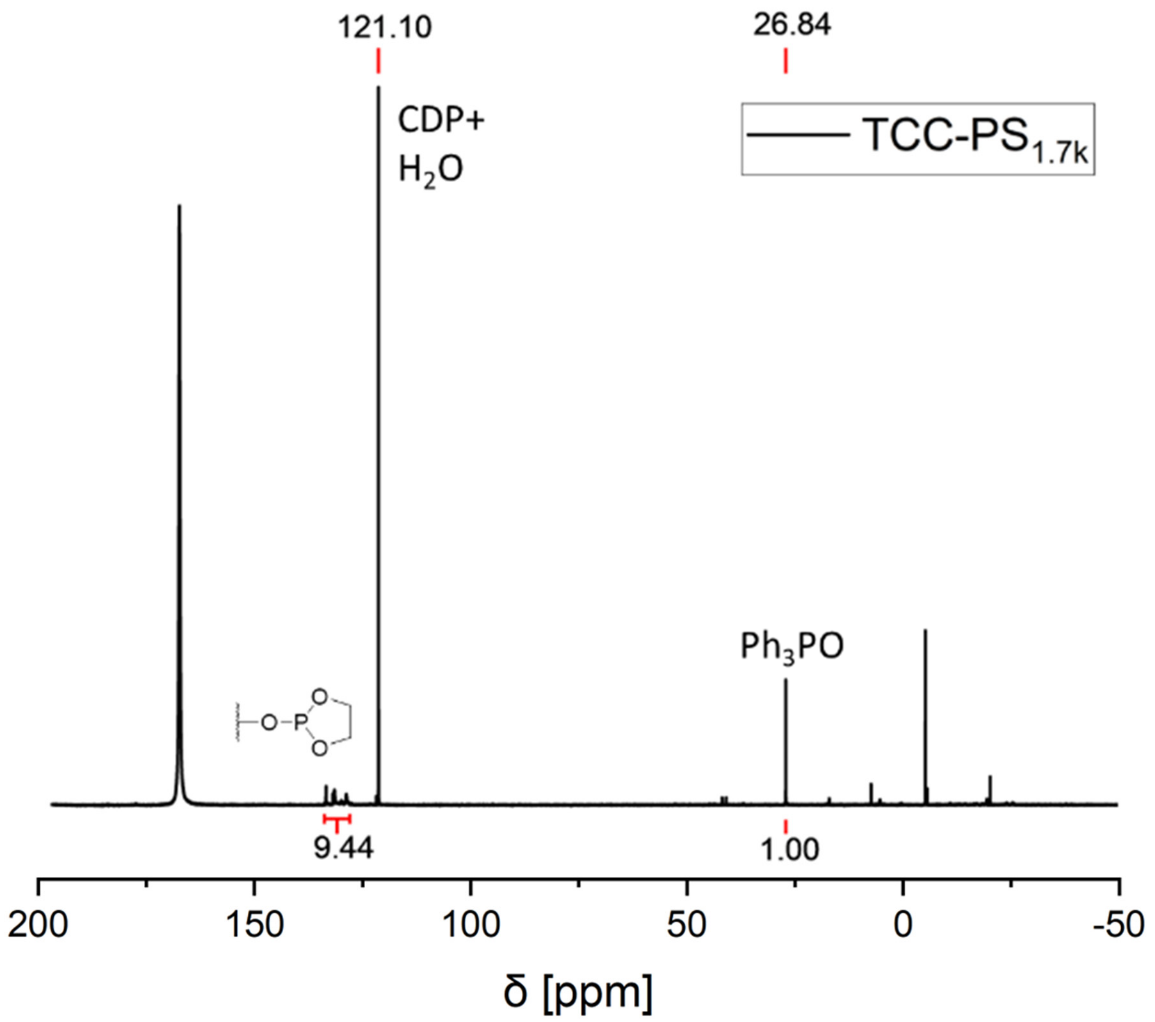

Figure S31. ^{31}P-NMR of TCC-PS$_{1.7k}$ derivatized with 2-chloro-2-oxo-1,3,2-dioxaphospholane (CDP) using Cr(acac)$_3$ and Ph$_3$PO as internal standard.

TCC-PS$_{3.6k}$ polymer

M_n = 13,930 g/mol

M_w = 33,730 g/mol

$Đ$ = 2.42

T_g = 88.5 °C

TGA: max. degradation rate at 379.1 °C, 5 wt% loss at 319.8 °C.

M (BQA) = 256 g/mol, M_n (TCC-PS$_{3.6k}$) = 13900 g/mol,

Measured via ^{31}P-NMR: average of 4.0 BQA per chain = 7.4 wt%.

Calculated with ^{1}H-NMR data: 2 TCC groups per 34 styrene groups = 5.6 mol% TCCs per aromatics.

Calculated: average of 3.6 BQA per chain = 6.6 wt%.

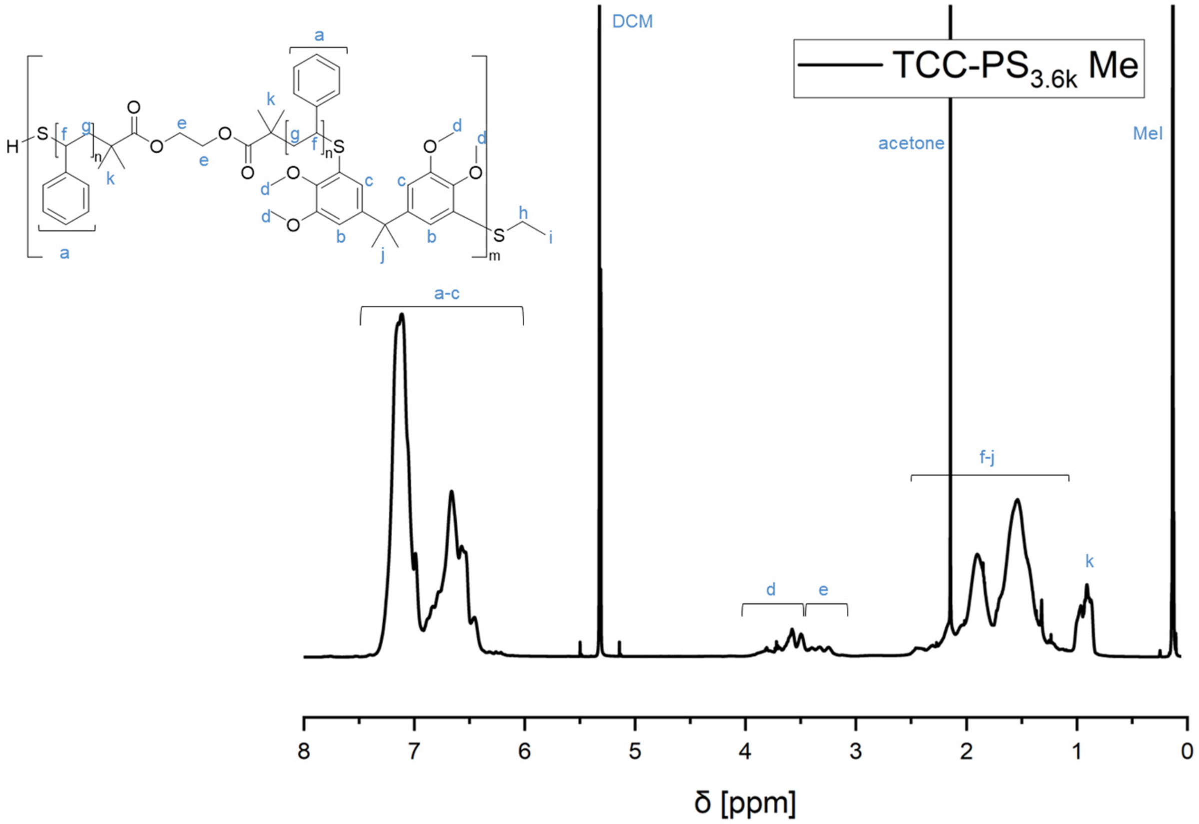

Figure S32. ^{1}H-NMR spectrum of TCC-$PS_{3.6k}$ after methylation of the catechol groups with MeI.

^{1}H-NMR: (500 MHz, CD_2Cl_2, δ in ppm):

7.37-6.26 (m, Ar-H), 3.99-3.66 (m, Ar-O-CH_3), 3.66-3.14 (m, 4H, O-C-H), 2.53-1.21 (m, backbone), 1.05-0.80 (m, 12H, -CH_3).

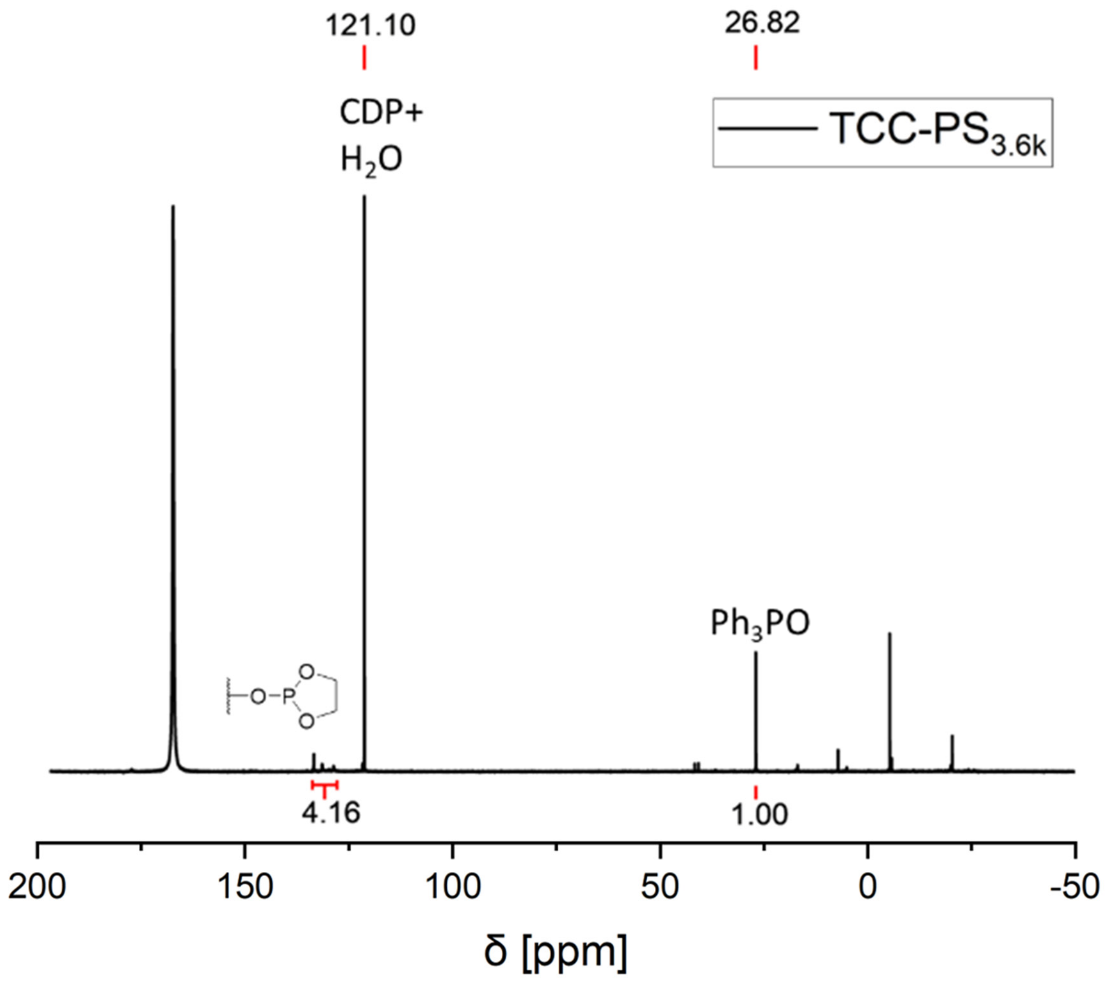

Figure S33. ^{31}P-NMR spectrum of TCC-PS$_{3.6k}$ derivatized with 2-chloro-2-oxo-1,3,2-dioxaphospholane (CDP) using Cr(acac)$_3$ and Ph$_3$PO as internal standard.

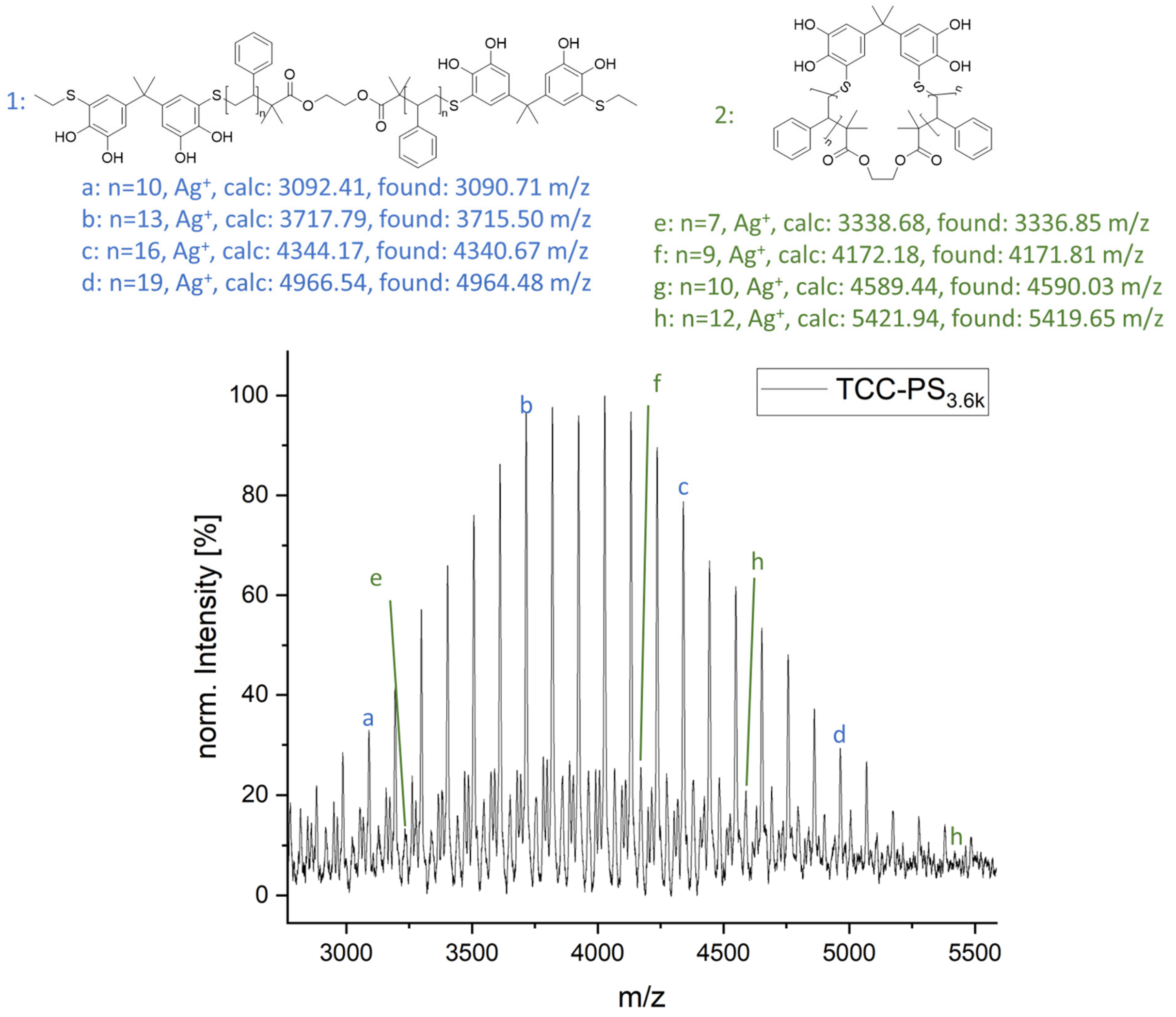

Figure S34. MALDI-ToF-MS spectrum of TCC-PS$_{3.6k}$ measured in linear positive mode with AgTFA. The linear structure (1) and a cyclic structure (2) could be assigned. The cyclic structure likely forms during the polyaddition reaction, no ethanethiol end groups are present.

MALDI-ToF-MS:

Structure 1 (linear): *m/z* 2986.3 (Ag^+, 28%), 3090.7 (33), 3194.3 (41), 3299.3 (57), 3403 (66), 3508 (76), 3611.5 (68), 3715.5 (96), 3820.4 (98), 3924.1 (96), 4028.5 (100), 4132.5 (97), 4236.6 (90), 4340.7 (79), 4445.1 (67), 4549.4 (62), 4653.1 (53), 4757.5 (48), 4861.8 (37), 4964.5 (29), 5069.3 (27), 5175.9 (17), 5276.6 (16), 5381.2 (14), 5483.9 (12).

Structure 2 (cyclic): *m/z* 2918.5 (Ag^+, 13%), 3024.9 (12), 3129.2 (14) 3233.7 (13), 3336.9 (13), 3441.9 (16), 3548.0 (19), 3652.0 (20), 3755.7 (20), 3861.2 (24), 3963.6 (25), 4067.3 (25), 4172.1 (26), 4274.5 (24), 4381.3 (23), 4484.2 (25), 4590.0 (23), 4692.5 (22), 4796.7 (18), 4903.4 (16), 5006.2 (17), 5110.8 (13), 5214.0 (11), 5316.5 (11), 5419.7 (9).

TCC-$PS_{6.3k}$ polymer

M_n = 22,930 g/mol

M_w = 55,780 g/mol

$Đ$ = 2.43

T_g = 91.9 °C

TGA: max. degradation rate at 395.0 °C, 5 wt% loss at 333.1 °C.

M (BQA) = 256 g/mol, M_n (TCC-$PS_{6.3k}$) = 22900 g/mol

Measured via ^{31}P-NMR: average of 3.2 BQA per chain = 3.6 wt%.

Calculated with ^{1}H-NMR data: 2 TCC groups per 58 styrene groups = 3.3 mol% TCCs per aromatics.

Calculated: average of 3.5 BQA per chain = 3.9 wt%.

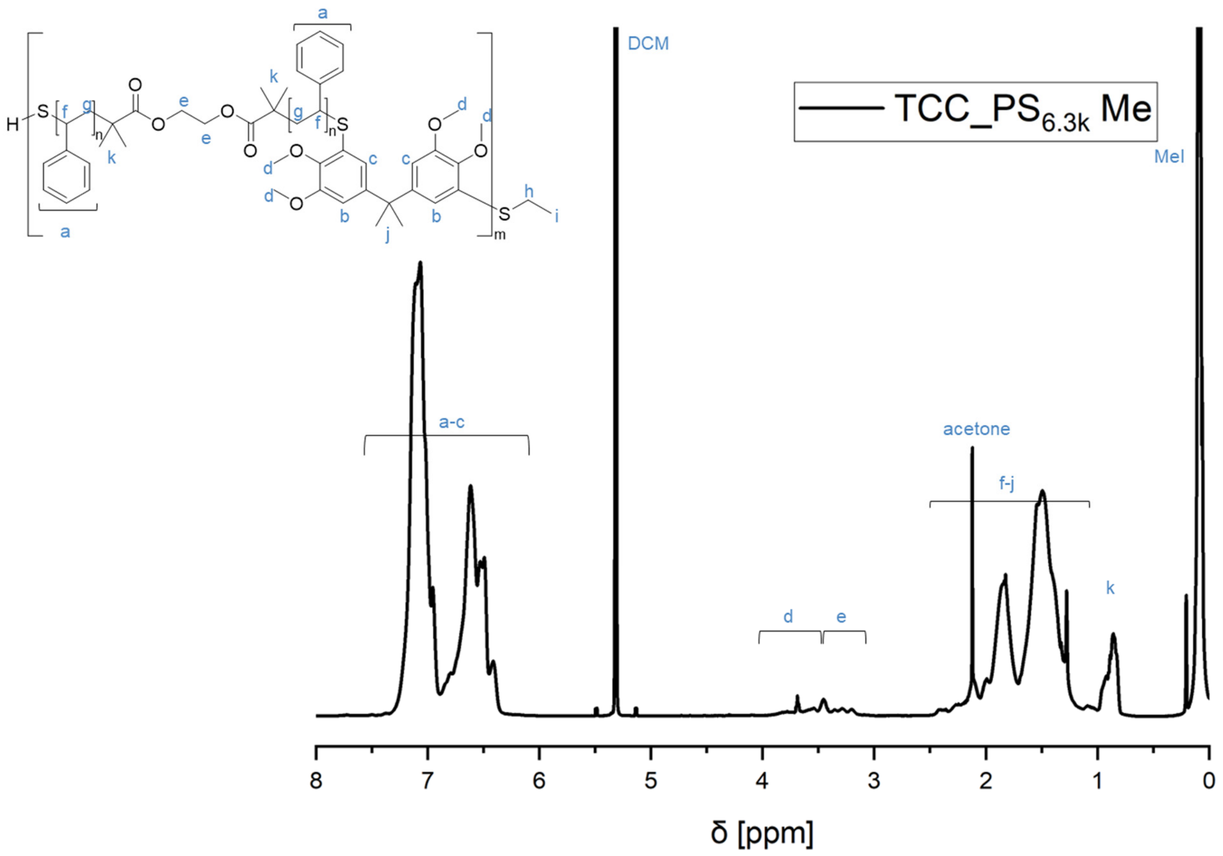

Figure S35. ^{1}H-NMR spectrum of TCC-$PS_{6.3k}$ after methylation of the catechol groups with MeI.

^{1}H-NMR: (500 MHz, CD_2Cl_2, δ in ppm):

7.39-6.29 (m, Ar-H), 4.02-3.61 (m, Ar-O-CH3), 3.61-3.10 (m, 4H, O-C-H), 2.48-1.14 (m, backbone), 1.00-0.67 (m, 12H, -CH_3).

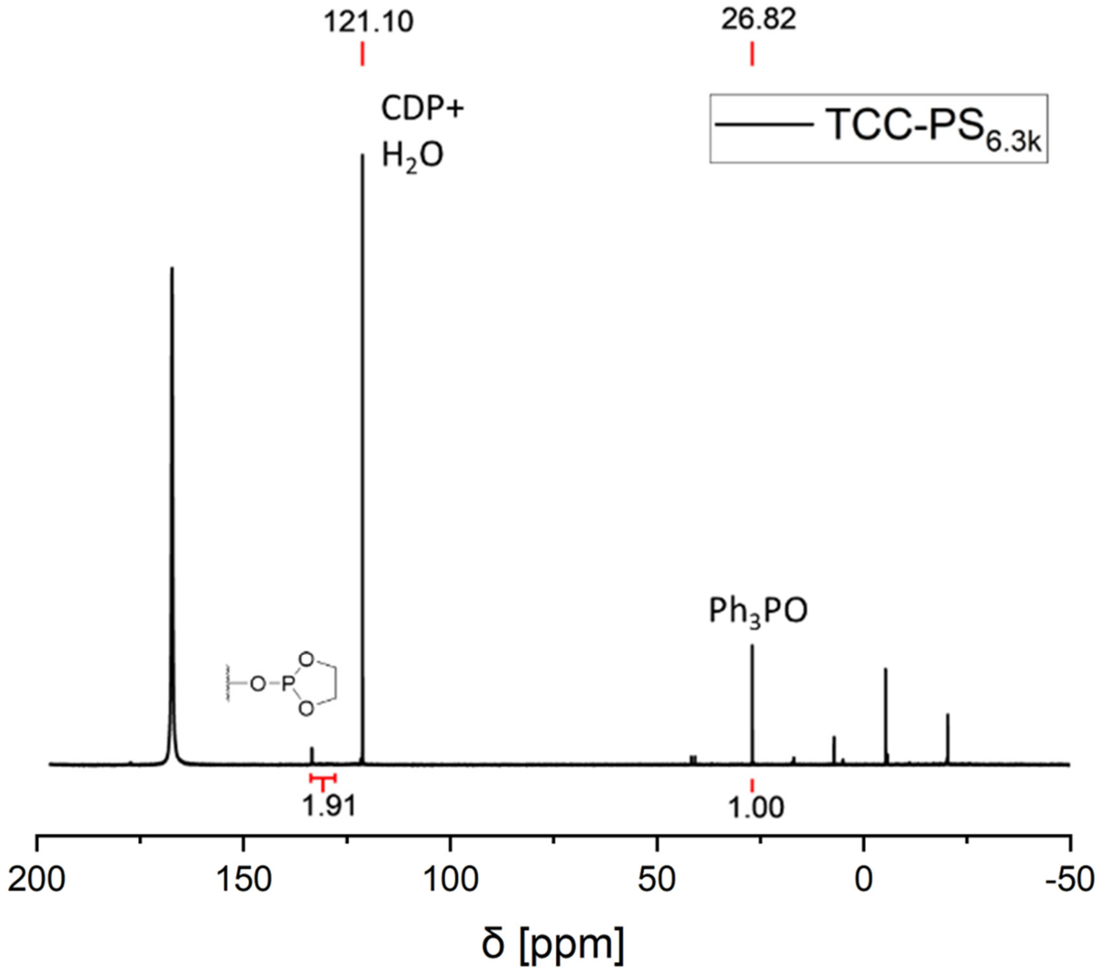

Figure S36. ^{31}P-NMR spectrum of TCC-PS$_{6.3k}$ derivatized with 2-chloro-2-oxo-1,3,2-dioxaphospholane (CDP) using Cr(acac)$_3$ and Ph_3PO as internal standard.

Further TCC-PS characterization

^{1}H-NMR

Qualitative proof of catechols via methylation of $PS_{1.7k}$

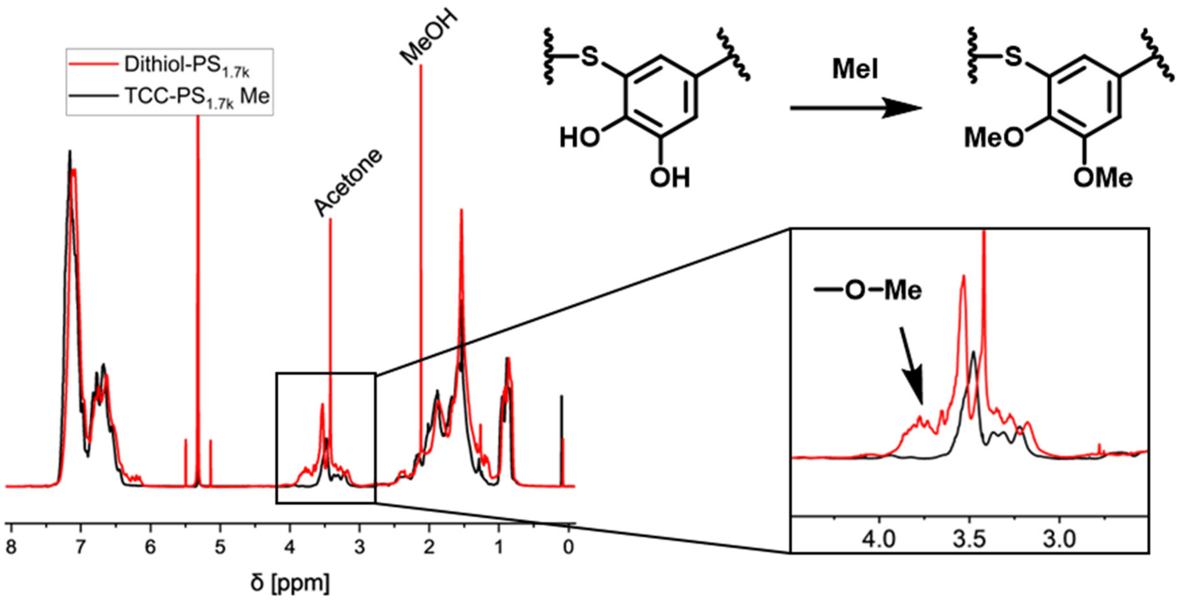

Figure S37: ^{1}H-NMR spectra of Dithiol-$PS_{1.7k}$ (black) and the methylated version of the TCC polymer, TCC-$PS_{1.7k}$ (red). The aromatic BQA signals are overlapped by the aromatic styrene signals, the same applies for the BQA methyl protons which cannot be assigned due to the polymeric backbone overlap. After methylation of the catecholic hydroxy groups, new methoxy signals appear from δ = 4.00 – 3.50 ppm, indicating the presence of methylated catechols. Due to overlap, they do not allow a quantification of TCC groups.

$FeCl_3$ test

Qualitative proof of catechols via $FeCl_3$ test:

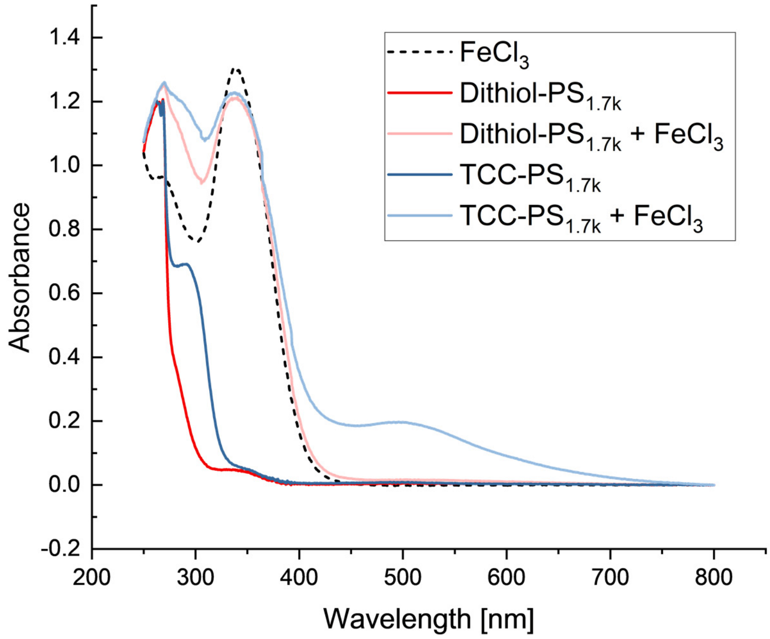

Figure S38: UV/Vis spectra of pure $FeCl_3$, and Dithiol-$PS_{1.7k}$/TCC-$PS_{1.7k}$ before and after addition of $FeCl_3$ solution in NMP. For the catechol bearing polystyrene, a change of color is observed along with the appearance of a new signal at around 500 nm which indicates the presence of catechols.

^{31}P-NMR measurements overview

Table S2. Overview of parameters used for calculation of the average amount of BQA per TCC-PS_x chain.

	M_n TCC-PS [g/mol]	*m* TCC-PS [mg]	*n* TCC-PS [mmol]	Integral OH	*n* OH [mmol]	OH per av. TCC-PS chain	Number of BQA per av. TCC-PS chain	Amount BQA per av. TCC-PS chain [wt%]
TCC-$PS_{1.7k}$	9100	42.3	0,00465	9.37	0.0937	20.15	5.04	14.3
TCC-$PS_{3.6k}$	13900	36.1	0,00260	4.20	0.0420	16.15	4.04	7.4
TCC-$PS_{6.3k}$	22900	34.9	0,00152	1.95	0.0195	12.83	3.20	3.6

Others:

FTIR [cm^{-1}]: 3726, 3628, 3620, 3603, 3082, 3069, 3061, 3030, 2997, 2978, 2968, 2934, 2922, 2883, 2872, 2854, 2357, 2345, 2330, 1730, 1601, 1493, 1475, 1452, 1366, 1230, 1217, 1205, 1124, 756, 696, 667.

IR measurements indicate the addition of a phenolic species, most likely the catechol groups from rearomatization of the BQA monomers (appearance of a new aromatic stretching vibration at 1508 cm^{-1}, a phenolic O-H bending vibration at 1365 cm^{-1} and new fingerprint bands at 1216 cm^{-1} and 1230 cm^{-1} match the vibration of the BQA-skeleton at 1221 cm^{-1} and 1239 cm^{-1}, respectively).[8-10]

Raman [cm^{-1}]: 3055, 2999, 2975, 2910, 2854, 1602, 1582, 1447, 1331, 1199, 1155, 1031, 1002, 908, 794, 759, 618.

Unfortunately, the TCC-polymer exhibits strong fluorescence in its Raman spectra, which could not be fully mitigated by experimental adjustments. The expected new band between 1080-1100 cm^{-1}, which would indicate the presence of an aromatic thiol or thioether, is completely obscured by bands present in all of the samples and becomes more elusive with increased fluorescence. Nonetheless, the appearance of new phenolic and aromatic bands strongly supports the proposed mechanism of polymerization via Michael-like addition and subsequent tautomerization and rearomatization steps.

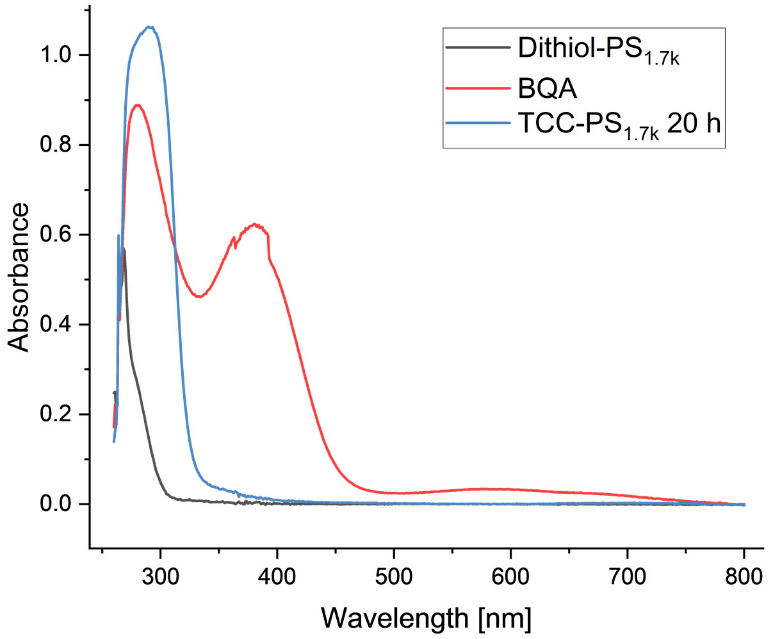

Figure S39. UV/Vis spectra of BQA, Dithiol-PS$_{1.7k}$ and TCC-PS$_{1.7k}$ in DMF show the disappearance of the characteristic BQA signal at around 380 nm after the polyaddition reaction.

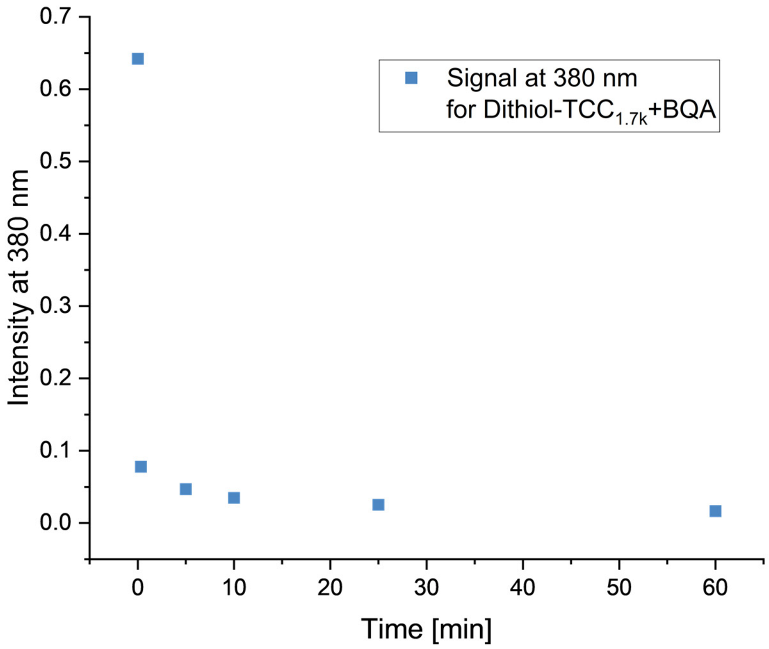

Figure S40. UV/Vis measured kinetics of the polyaddition reaction of Dithiol-PS$_{1.7k}$ at 380 nm to observe the decline of the BQA signal in die reaction process.

Total overview over synthesized polymers

Table S3. Length and distribution of synthesized polystyrene polymers measured via THF-SEC, rounded to the nearest hundred.

	Polymer	M_n [g/mol]	M_w [g/mol]	M_p [g/mol]	$Đ$
DiCTA-PS	PS$_{1.7k}$	2000	2200	2200	1.07
	PS$_{3.6k}$	3700	4000	4100	1.08
	PS$_{6.3k}$	6500	7100	7300	1.09
Dithiol-PS	PS$_{1.7k}$	1700	1880	1800	1.08
	PS$_{3.6k}$	3600	4200	3700	1.14
	PS$_{6.3k}$	6300	7200	6600	1.15
TCC-PS	PS$_{1.7k}$	9100	14700	11200	1.61
	PS$_{3.6k}$	13900	33700	26800	2.42
	PS$_{6.3k}$	22900	55800	43900	2.43

Reaction analysis

Reaction optimization

Stochiometric optimization

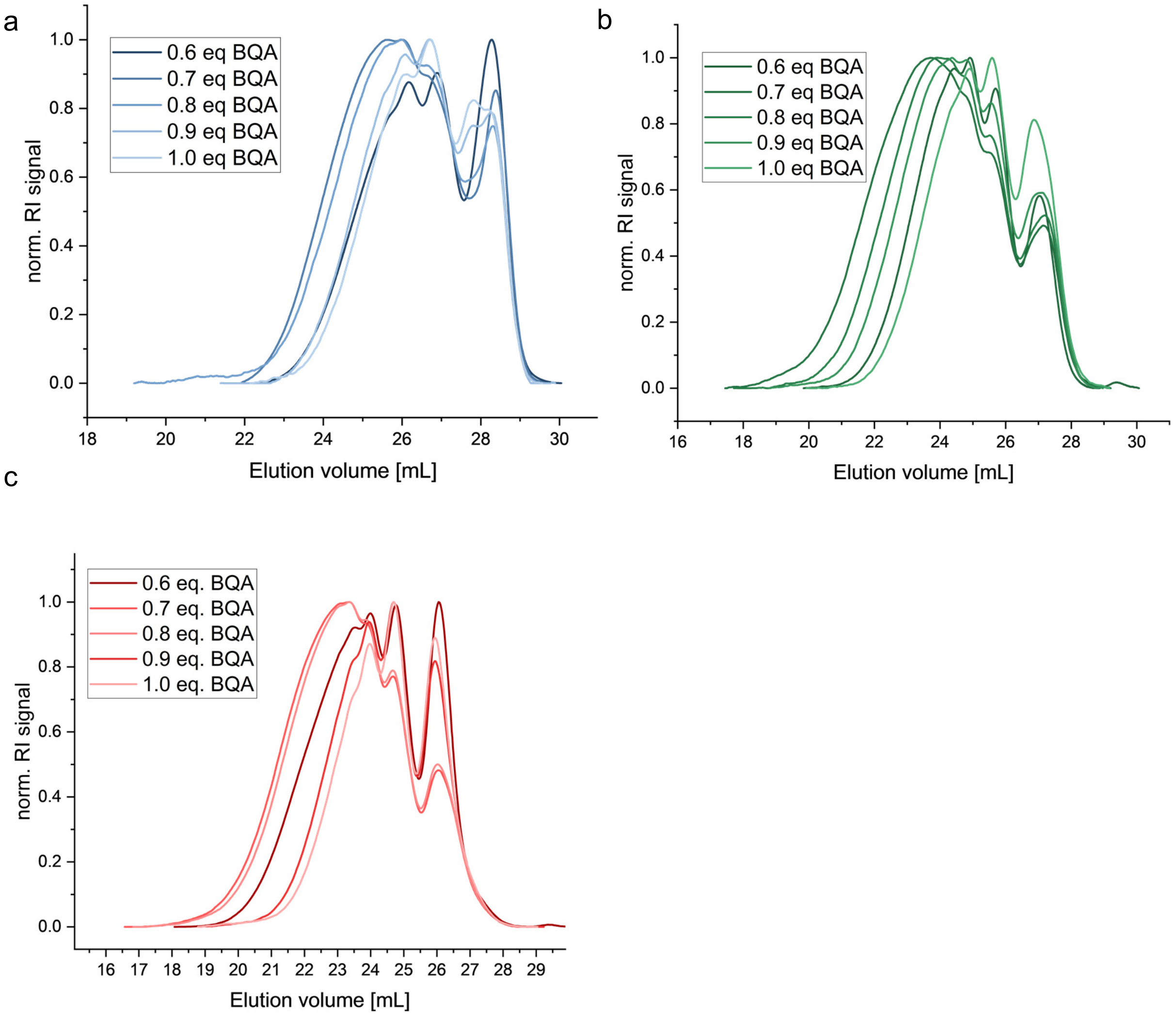

Figure S41. Dithiol-PS$_{1.7k}$ (a), Dithiol-PS$_{3.6k}$ (b) and Dithiol-PS$_{6.3k}$ (c) with 0.6-1.0 eq. of BQA, stirred for 18 h in DMF without precipitation. A thiol excess with 0.7-0.8 eq. BQA leads to overall highest molecular weights.

Table S4. Overview of SEC measured molecular weights and distributions of each TCC-PS$_x$ product.

	eq. BQA	M_n [g/mol]	M_w [g/mol]	*Đ*
TCC-PS$_{1.7k}$	0.6	3500	6700	1.92
	0.7	4400	9600	2.20
	0.8	4300	10.000	2.33
	0.9	3700	6800	1.86
	1.0	3500	6300	1.81
TCC-PS$_{1.7k}$	0.6	9400	18100	1.92
	0.7	11800	36700	3.10
	0.8	10500	27000	2.58
	0.9	9300	22000	2.36
	1.0	7200	14300	1.98
TCC-PS$_{1.7k}$	0.6	14300	32200	2.25
	0.7	18500	47700	2.58
	0.8	17800	44700	2.52
	0.9	12700	24000	1.90
	1.0	11800	22000	1.86

Temperature optimization

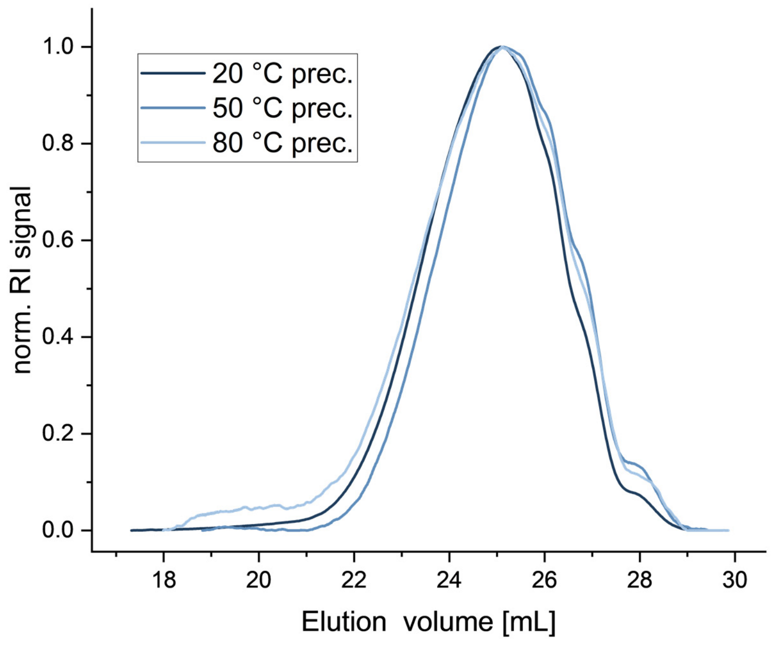

Figure S42: Dithiol-PS$_{1.7k}$ with 0.7 eq. BQA stirred over night at different temperatures and precipitated in MeOH. No temperature effect was observed.

Concentration optimization

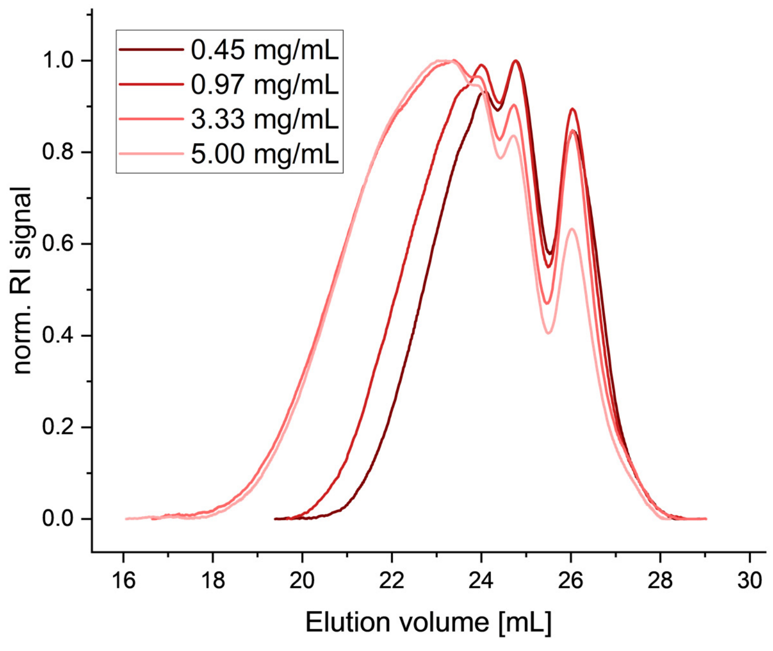

Figure S43. Dithiol-PS$_{6.3k}$ macromonomer at various BQA end concentrations.

A concentration of *c(BQA)* = 5.00 mg/mL resulted in the crude polyaddition product with the highest number average molar mass of M_n = 19,400 g/mol, compared to *c(BQA)* = 3.33 mg/mL, *c(BQA)* = 0.97 mg/mL and *c(BQA)* = 0.45 mg/mL with polymers of M_n = 17,600 g/mol, M_n = 13,100 g/mol and M_n = 11,600 g/mol, respectively. Especially for lower concentrations of reactants in the mixture, high molecular weights are not achieved, the dispersity for *c(BQA)* = 0.97 and 0.45 mg/mL is with $Đ$ = 2.08 and $Đ$ = 1.86 relatively low.

SEC polymerization kinetics of each Dithiol-PS$_x$ with BQA

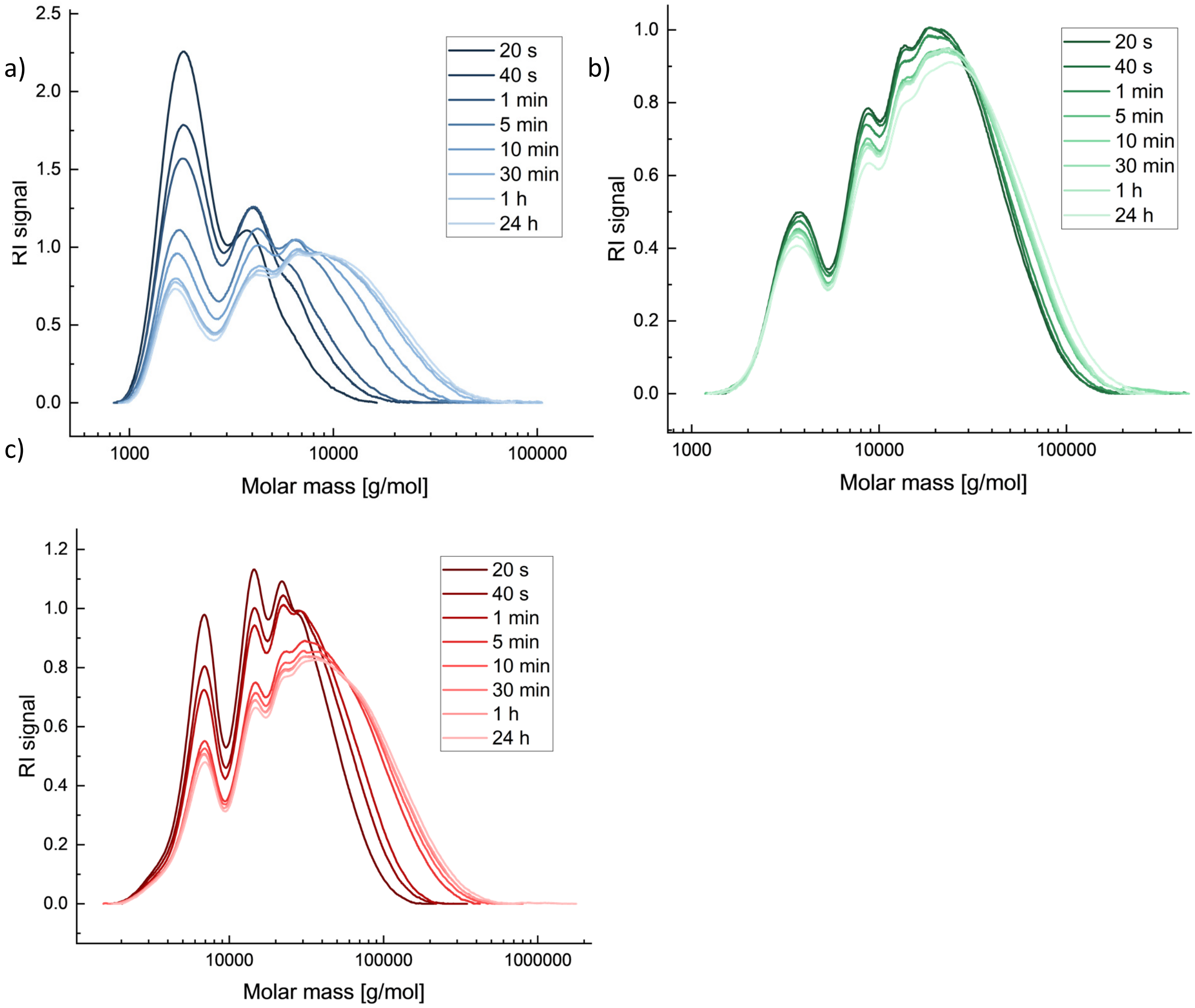

Figure S44. Polymerization kinetics of Dithiol-PS$_{1.7k}$ (a), Dthiol-PS$_{3.6k}$ (b) and Dithiol-PS$_{6.3k}$ (c) with 0.7 eq. BQA to form TCC-PS$_x$ without purification. The polymerization reaction takes places very rapidly and is mostly completed after approximately 1 h for the slowest reaction.

SEC – polymerization overview

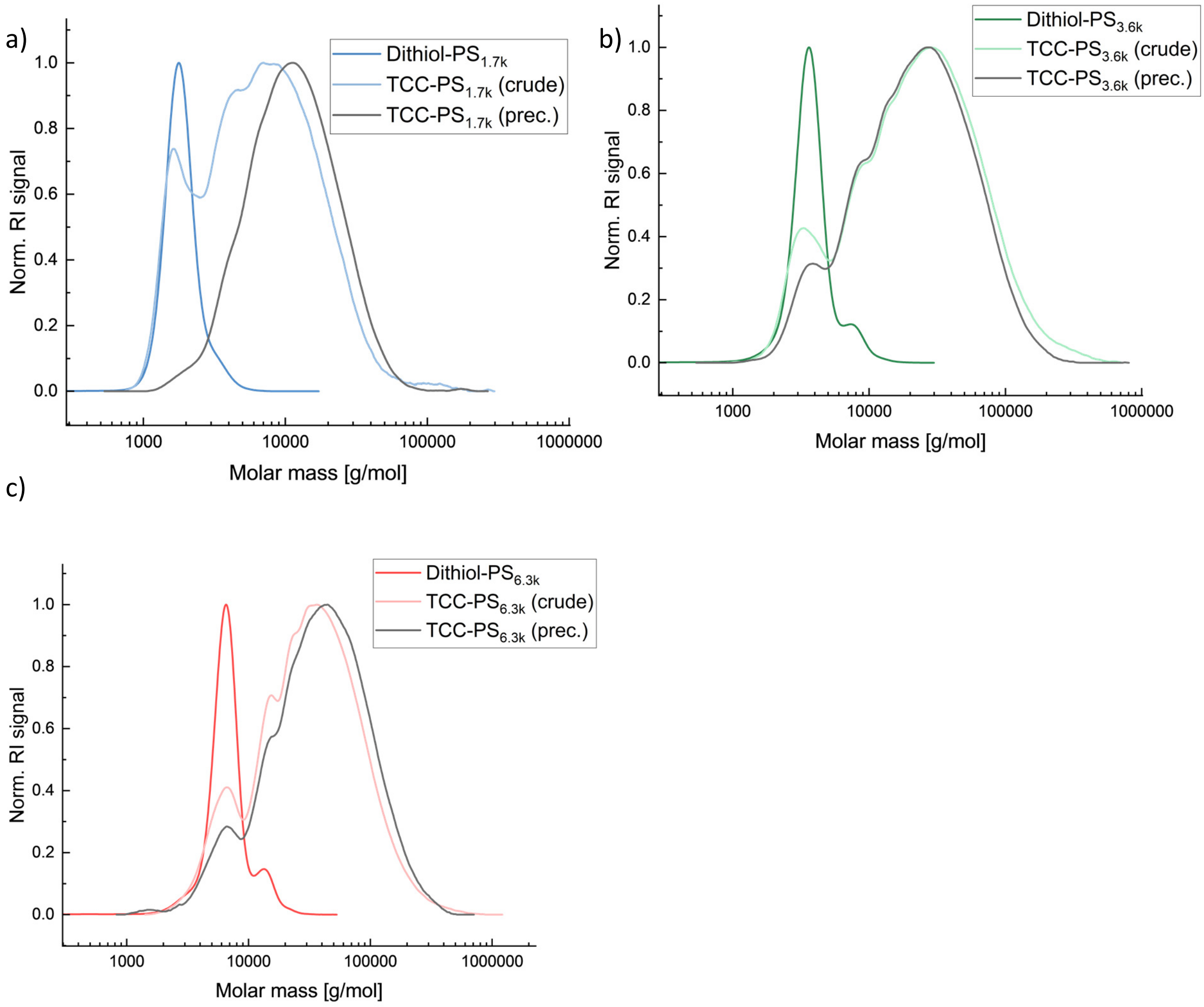

Figure S 45: Overview of PS$_{1.7k}$ (a), PS$_{3.6k}$ (b) and PS$_{6.3k}$ (c) as Dithiol-PS$_x$ and TCC-PS$_x$, after 18h of polyaddition reaction with BQA and TCC-PS$_x$ after precipitation twice in cold methanol (grey). For the TCC-PS$_{1.7k}$ polymer, the precipitation removes a high amount of polymer species with a low degree of polymerization and therefore increases the molecular weight drastically. The amount of small macromonomer species in the overall precipitated polymer is less than 1 %, calculated with the integral ratios of the SEC curves.

Synthesis of pure polystyrene as reference sample

20.0 g of styrene (0.192 mol, 388 eq) was destabilized over basic aluminum oxide. 120 mg of dibenzoyl peroxide (0.495 mmol, 1 eq) was added and the solution was degassed for 15 min. Subsequently, the mixture was heated to 90 °C and stirred for one hour. The polymer was dissolved in tetrahydrofuran and precipitated in cold methanol.

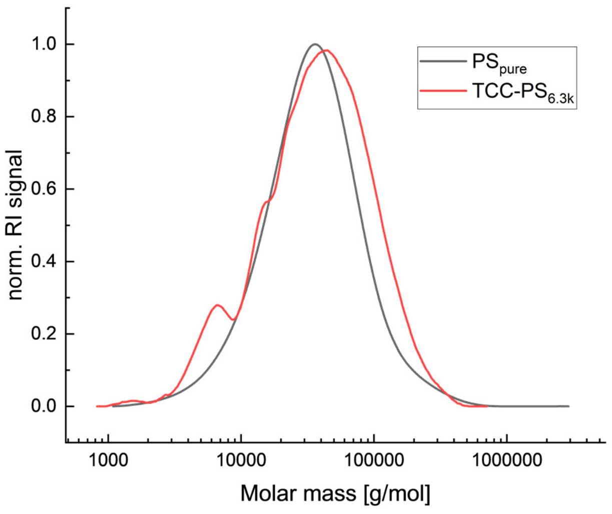

Figure S46. SEC spectra of TCC-PS$_{6.3k}$ and pure PS. They align in molar mass distribution.

PS$_{pure:}$

M_n = 23,000 g/mol

M_w = 49,000 g/mol

$Đ$ = 2.12

Adhesion tests

All tests were conducted according to the same procedure if not stated otherwise: The samples were solved in acetone with a concentration of 25 mg/100 µL. On each plate, an area of around 6 mm x 20 mm was covered with 25 µL solution. After waiting until the solvent was partially evaporated, two specimens were placed together and fixed with two fold back clips. The system was placed in the oven at 130 °C for 24 h. A lap-shear test was conducted to measure the adhesion force of the cured glue.

Shear test results

Pure PS

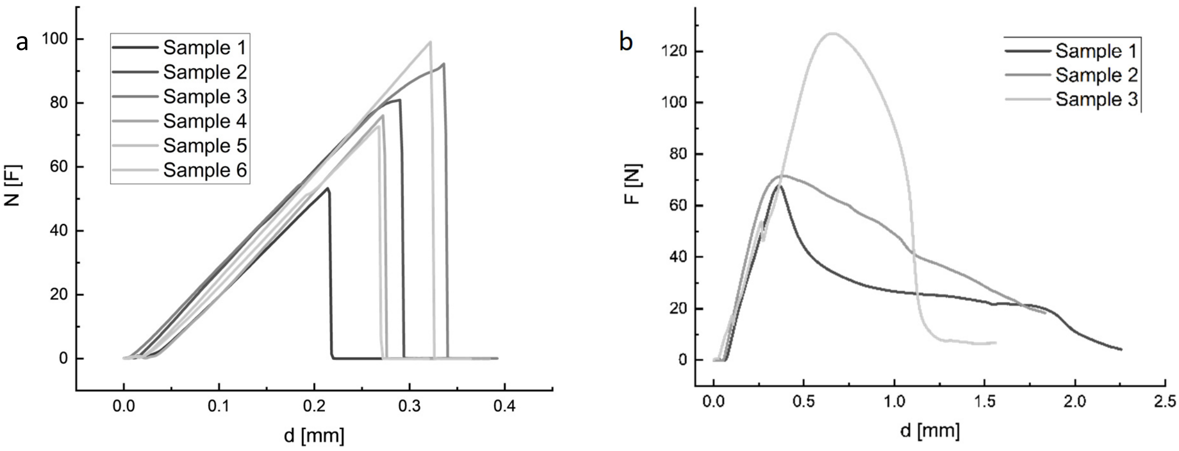

Figure S47: Pure polystyrene with a similar chain length and dispersity as TCC-PS$_{6.3k}$ was evaluated under the same conditions. Only weak adhesion was found in dry application (a). For wet application, the overlap had with a width of 20 mm and a length of 6.8 – 7.5 mm a slightly higher area than TCC-PS samples due to an easier breakage during handling (b).

Aluminum dry

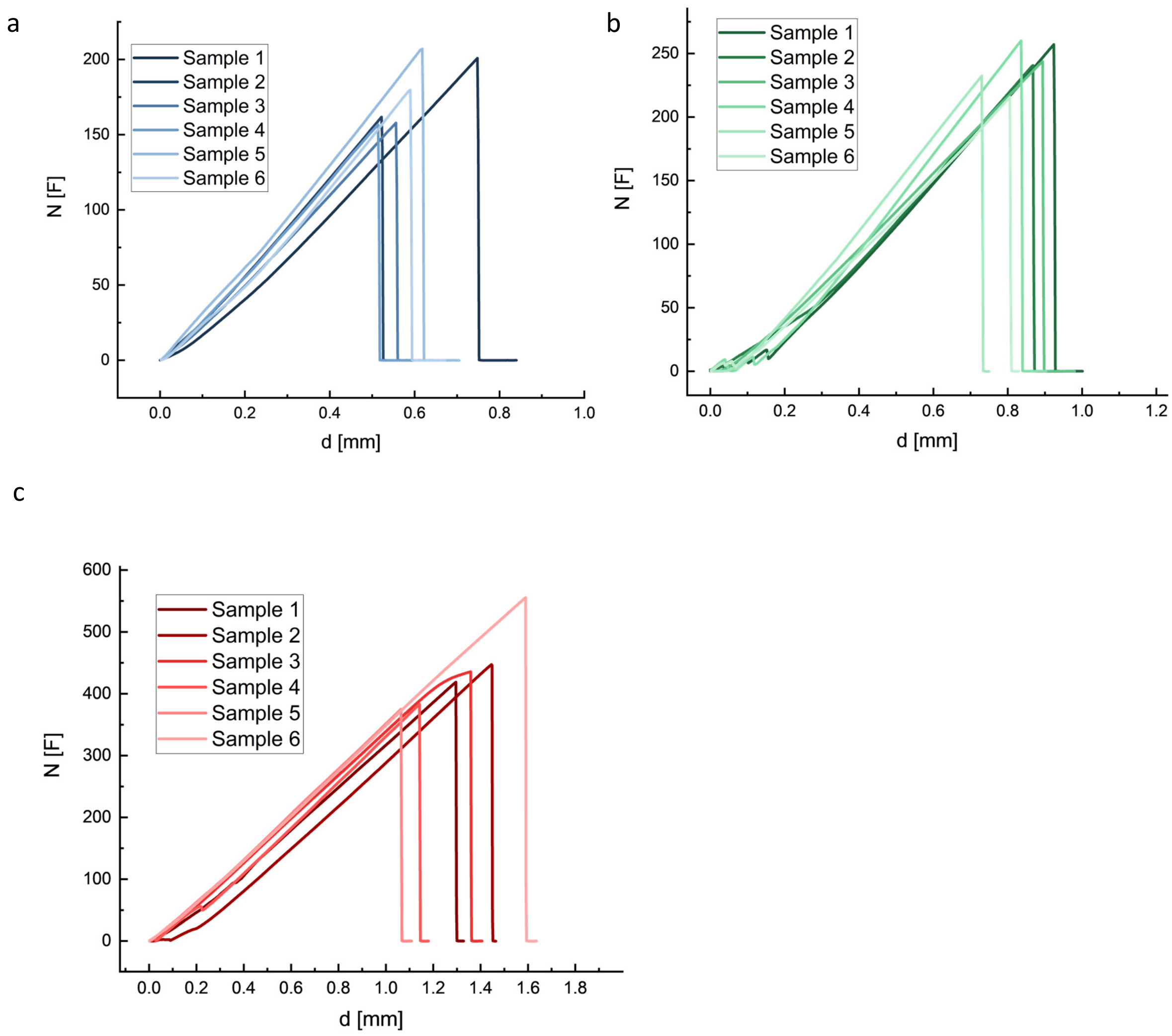

Figure S48. Shear test results of TCC-PS$_{1.7k}$(a), TCC-PS$_{3.6k}$ (b) and TCC-PS$_{6.3k}$ (c) glued dry on aluminum.

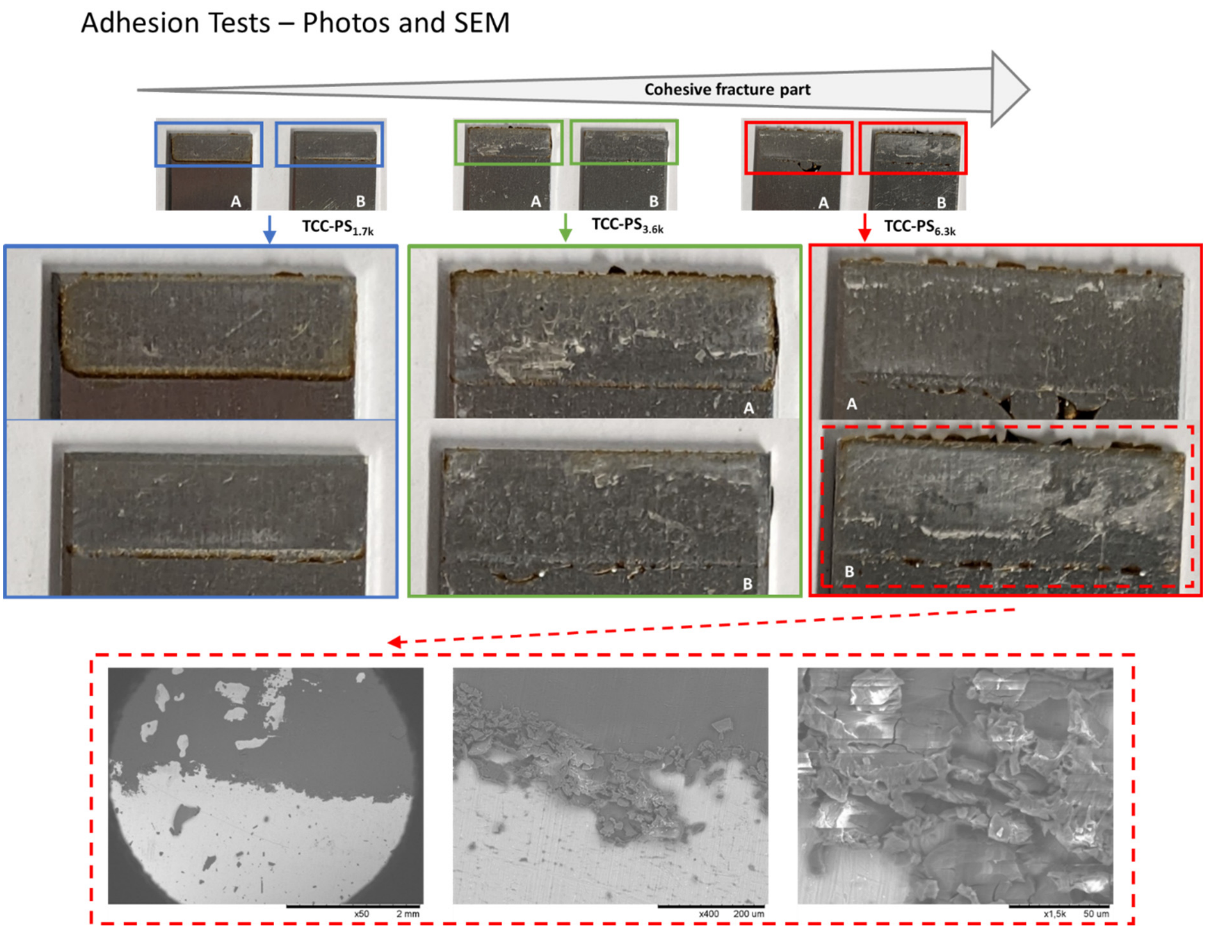

Figure S49. Adhesive/Cohesive fracture pattern in dependency of the PS block length. TCC-PS$_{1.7k}$ as a low adhesive material shows a purely adhesives fracture pattern. With increasing adhesion results, the cohesive part of the polymer decreases and results in a cohesive/adhesives fracture pattern. The fracture was observed in a SEM measurement and shows the mixed character.

Other materials

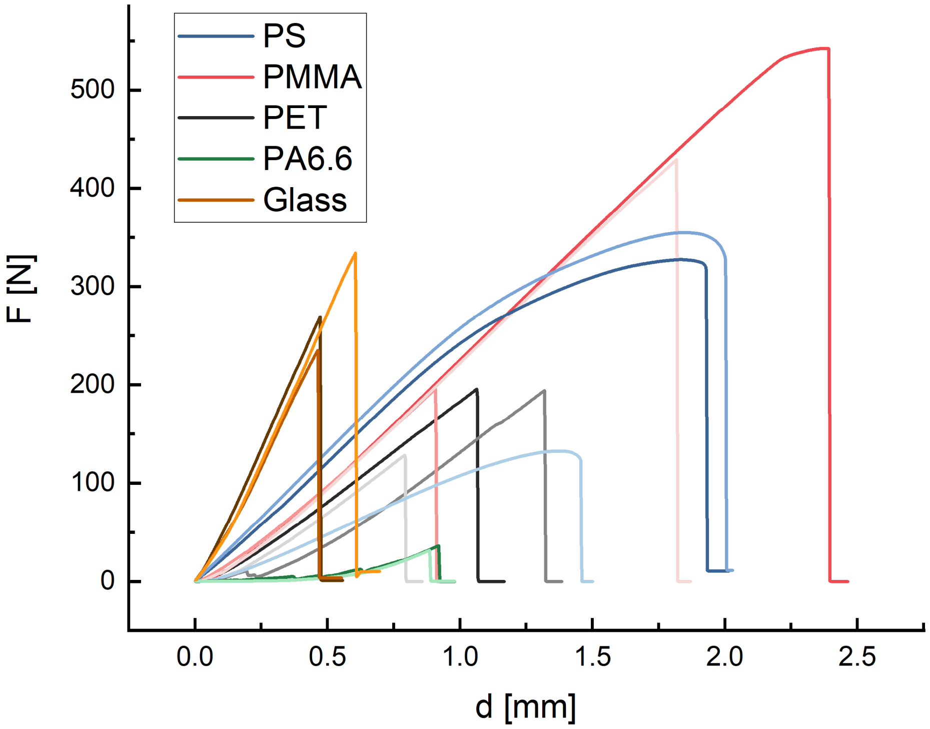

Figure S50. Besides aluminum, the TCC-PS$_{6.3k}$ was also evaluated on different materials under the same conditions but with a curing at 60 °C for 3 d due to softening of the specimen at higher temperatures. On polystyrene surfaces the overall highest average results were found.

Aluminum under water

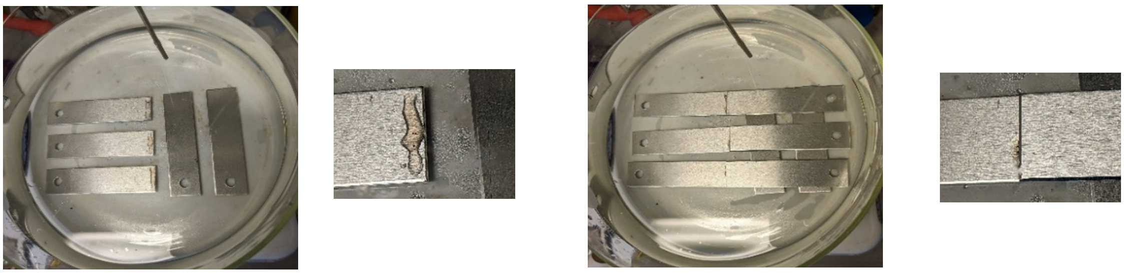

Figure S51. Picture of the underwater application of aluminum. The polymer was dissolved in chloroform, applied under water onto the aluminum specimen and covered with a second specimen.

Underwater tests were conducted in deionized water under similar conditions as described by Wilker *et al.* before.[11] The polymer was dissolved in chloroform (25 mg/100 µL) and then 25 µL were applied under water onto the aluminum specimen with an overlap of 4.4 - 6.2 mm x 20 mm. A second plate was then placed on top. The system was kept under water at 25 °C for 7 days so that the chloroform could diffuse out completely. The glued specimens were then removed from the water tank and dried in a tempered oven at room temperature for 3 days to reduce the effect of water as a plasticizer and to ensure a consistent and reproducible drying effect despite fluctuations in ambient temperature. A lap-shear test was conducted to measure the shear strength of the cured glue.

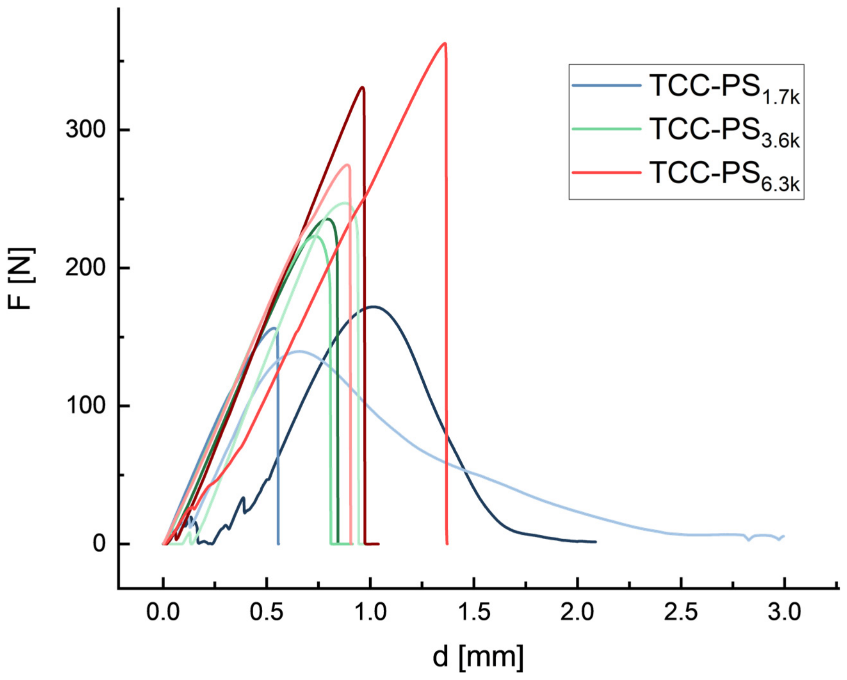

Figure S 52. Shear test results of the underwater applied glue after drying. The overlap of the two specimens had a width of 20 mm and a length between 4.4 - 6.2 mm.

References

1. Frigerio, M.; Santagostino, M.; Sputore, S., A User-Friendly Entry to 2-Iodoxybenzoic Acid (IBX). *J. Org. Chem.* **1999,** *64* (12), 4537-4538.
2. Krüger, J. M.; Börner, H. G., Accessing the Next Generation of Synthetic Mussel-Glue Polymers via Mussel-Inspired Polymerization. *Angew. Chem. Int.* **2021,** *60* (12), 6408-6413.
3. Lai, J. T.; Filla, D.; Shea, R., Functional Polymers from Novel Carboxyl-Terminated Trithiocarbonates as Highly Efficient RAFT Agents. *Macromolecules* **2002,** *35* (18), 6754-6756.
4. Zhou, C.; Qian, S.; Zhang, A.; Xu, L.; Zhu, J.; Cheng, Z.; Kang, E.-T.; Yao, F.; Fu, G. D., A well-defined amphiphilic polymer co-network from precise control of the end-functional groups of linear RAFT polymers. *RSC Adv.* **2014,** *4* (16), 8144-8156.
5. Vandenbergh, J.; Ramakers, G.; van Lokeren, L.; van Assche, G.; Junkers, T., Synthesis of degradable multi-segmented polymers via Michael-addition thiol–ene step-growth polymerization. *RSC Advances* **2015,** *5* (100), 81920-81932.
6. Wu, F.; Wang, H.; Zheng, X., Concentration-dependent frequency shifts of the C□S stretching modes in ethylene trithiocarbonate studied by Raman spectroscopy. *J. Raman Spectrosc.* **2015,** *46* (6), 591-596.
7. HORIBA Raman Data and Analysis – Raman bands. . https://static.horiba.com/fileadmin/Horiba/Technology/Measurement_Techniques/Molecular_Spectroscopy/Raman_Spectroscopy/Raman_Academy/Raman_Tutorial/Raman_bands.pdf (accessed August 31, 2023).
8. Hesse, M.; Meier, H.; Zeeh, B., *Spektroskopische Methoden in organischen Chemie*. 7th ed.; Georg Thieme Verlag: Stuttgart, 2005.
9. Aldrich, S. IR Spectrum Table. https://www.sigmaaldrich.com/DE/en/technical-documents/technical-article/analytical-chemistry/photometry-and-reflectometry/ir-spectrum-table (accessed August 31, 2023).
10. Technology", N. I. o. A. I. S. a. SDBS No. 1716 (4,4’-isopropylidenediphenol). https://sdbs.db.aist.go.jp (accessed August 31, 2023).
11. North, M. A.; Del Grosso, C. A.; Wilker, J. J., High Strength Underwater Bonding with Polymer Mimics of Mussel Adhesive Proteins. *ACS Appl. Mater. Interfaces* **2017,** *9* (8), 7866-7872.
